# Wealth-anchored category validation: A diagnostic for categorical orderings in fragile-state household surveys

**DOI:** 10.1371/journal.pone.0360752

**Published:** 2026-10-29

**Authors:** Gudi V. Chandra Sekhar

**Affiliations:** Department of Economics, College of Business and Economics, Gambella University, Gambella, Ethiopia; Akademia Jagiellońska w Toruniu: Akademia Jagiellonska, CZECHIA

## Abstract

Asset-based wealth indices and binary “improved” or “clean” service indicators both assume that the response options of a categorical survey variable are coded in the wealth order they imply. The assumption is rarely tested. I set out a procedure, Wealth-Anchored Category Validation (WACV), that tests it, and I am careful about what a failed test means. WACV ranks a variable’s categories by their average position on a wealth index built from the other indicators, so no variable is judged against itself, and attaches stratified cluster-bootstrap intervals in which the whole estimation chain, including the index itself, is re-estimated in every replication. It reports two things: whether the categories sit on the local wealth gradient in the order the coding assumes, and whether a binary someone has already built from the variable falls where that gradient actually steps. Divergence on either is a diagnostic signal with several possible explanations: a coding fault, an analytical recoding fault, a context-dependent classification, or noise. The procedure does not adjudicate between them from wealth data alone. Applied to five ordinal variables in three waves of the South Sudan High Frequency Survey, the ordering diagnostic finds no robust inversion in the main specification; equivalence tests and a simulation locate what that null can and cannot rule out. The cut-point diagnostic finds one severe anomaly: an improved-housing binary used in earlier analysis of these data places the two poorest dwelling types on the improved side, and sits 0.26 standard deviations below the households it excludes. That anomaly replicates in a survey wave not used to develop the method. A codebook audit shows the binary originates in analysis rather than in the survey instrument, which makes it an instructive case of analytical recoding rather than a defect in the data.

## 1 Introduction

Two instruments carry much of the weight in welfare measurement where consumption data are missing or distrusted. One is the asset-based wealth index, which compresses a set of ownership and amenity indicators into a single ranking of households [1–7]. The other is the binary service indicator, such as improved water, improved sanitation or clean cooking fuel, which collapses a categorical survey question into an adequate/inadequate split [8–10]. Both rest on a premise that is almost never examined on its own terms: that the response options of the underlying categorical variable have been coded in an order that corresponds to the welfare ordering the analyst intends to capture.

This paper takes that premise as an empirical question. The procedure I describe, Wealth-Anchored Category Validation (WACV), ranks the categories of a variable by their average position on a wealth index constructed from the other indicators, so that no variable is ever judged against itself. Where the resulting order diverges from the order the coding assumes, or where a binary drawn from the variable turns out to fall somewhere the wealth gradient does not step, the procedure flags the variable for investigation.

What follows from a flag is the point on which the paper turns, and it is worth stating plainly at the outset. A divergence between an assigned ordering and the local wealth ordering is not proof that the assigned ordering is wrong. Internationally standardised categories such as “clean cooking fuel” and “improved sanitation” describe technical, health and infrastructural properties. They were never claims about relative prosperity, and a category can sit high on a local wealth gradient without meeting them. Charcoal is the clearest instance: in urban South Sudan it is plausibly the fuel of comparatively well-off households, and it is not a clean fuel by any combustion or health standard. A wealth anomaly is a reason to look at a variable. It is not a verdict on it.

WACV therefore reports two diagnostics and adjudicates neither. The ordering diagnostic asks whether categories sit on the wealth gradient in the order the coding assumes. The cut-point diagnostic asks whether a binary indicator that someone has already constructed falls where the gradient actually steps. The second evaluates a cut; it never proposes one. No “validated improved set” is constructed anywhere in this paper, and no threshold relative to the sample mean is used.

The empirical application is to the South Sudan High Frequency Survey, a stratified two-stage survey of a fragile, largely rural, overwhelmingly poor population. Three results follow. First, in the main specification the ordering diagnostic finds no robust inversion in any of five ordinal variables (housing type, sanitation, drinking water, lighting and cooking fuel) once the design is honoured, the anchor’s own uncertainty is propagated, multiplicity is controlled, and categories too thin to support inference are set aside. A simulation study says what that null can and cannot rule out. Second, the cut-point diagnostic finds one severe anomaly: an improved-housing binary used in earlier analysis of these data assigns the improved label to tents and stick-walled tukuls, and the households it labels improved sit 0.26 standard deviations below those it excludes. That anomaly replicates, at almost identical magnitude, in a survey wave that played no part in developing the method. Third, an audit of the survey instrument and its codebook shows that this binary has no basis in the survey’s own coding; it was created during analysis. The case is therefore an example of analytical recoding gone wrong, not of a defective survey, and that distinction is one the paper now draws explicitly.

The contribution is methodological, and it is worth being precise about where it departs from existing practice. Work on validating asset indices asks whether the household ranking an index produces is robust to how the index is built, and whether it tracks consumption expenditure [11,12]; it takes the coding of each input variable as given. Category-scoring approaches, from the dummy-variable convention of the DHS wealth index [2] to polychoric principal components for ordinal inputs [13], estimate a score for each response option, but the polychoric variant assumes the assigned ordering is correct and the dummy variant leaves it untested, and in both the variable contributes to the very index against which its own categories would have to be judged. Robustness analysis of deprivation indicators varies the cut-off and reports how the headline rate moves [9]; it does not ask whether the categories on either side of the cut sit where the wealth gradient says they should. WACV asks that prior question, and differs from each of these in three respects: it tests the ordering of categories within a variable rather than the ranking of households across variables; it removes the variable under test from the anchor, so no variable is judged against itself; and it attaches design-based uncertainty to every pairwise comparison and to every cut, with the anchor re-estimated in every replication. WACV is cheap, requires no data beyond the survey itself, and belongs as a screening step in the wealth-index and multidimensional-poverty pipelines, where categorical orderings and binary cut-points are routinely imported from one survey context into another. The substantive lesson is narrower than the one the first version of this paper drew, and more defensible: an imported cut-point can run backwards against the local wealth gradient, and whether it does is an empirical matter worth checking before the indicator is trusted.

## 2 Materials and methods

### 2.1 What a divergence can mean

Because the interpretation of a flag is the crux of the method, this section fixes terminology before any estimation is described. Four distinct things can produce a divergence between an assigned categorical ordering and the local wealth ordering, and the literature on measurement error in asset indices does not consistently distinguish them [11,12,14,15]. Table 1 sets the four out.

**Table 1:** Four sources of divergence between an assigned ordering and the local wealth ordering.

Source	What has gone wrong	Where it lives	Can wealth data alone identify it?
Coding error	The survey instrument or its codebook assigns response options to categories incorrectly, or the recorded codes do not match the documented ones.	The survey	No; requires the questionnaire and codebook
Recoding error	The instrument is sound, but a derived variable built by the analyst maps the raw categories wrongly.	The analysis	No; requires the analysis code and its documentation
Context-dependent classification	Both instrument and derived variable are correct on their own terms; the global standard simply does not track prosperity in this population.	Neither; the standard is doing a different job	No; requires the technical or health definition
Sampling noise	Nothing is wrong; the apparent divergence is within the range the design’s uncertainty permits.	Nowhere	Yes; this is what the inference is for

Note. WACV separates the fourth from the first three by inference. Separating the first three from one another requires the survey documentation, the analysis code, or the technical standard, and the paper does that separation explicitly for the case it examines.

The practical consequence is that the procedure’s output is a flag plus a shortlist of explanations, and that resolving the flag is documentary work, not statistical work. In the application below, the housing anomaly is resolved as a recoding error by reading the questionnaire and the codebook; the cooking-fuel anomaly is resolved as a context-dependent classification by observing what the clean-fuel standard is for; and three variables produce no flag at all, which the inference section is careful not to call confirmation.

This also fixes the scope of the paper’s claims. The evidence here comes from one survey programme in one country, across three waves. It is not a comprehensive validation of any indicator definition, and nothing in it licenses a general claim about how well international service standards track wealth elsewhere.

### 2.2 The procedure

#### 2.2.1 The anchor.

Let a household i carry a vector of wealth indicators. For a categorical variable under test, every indicator derived from that variable is removed from the pool, and a survey-weighted first principal component is extracted from what remains [1,13]. The component is standardised to weighted mean zero and unit weighted standard deviation and oriented so that it correlates positively with the count of durable asset types owned, which fixes the sign of the index rather than leaving it to the arbitrary sign convention of the eigen-decomposition.

The component is a general wealth factor rather than a contrast between subsets of indicators. In Wave 3 the first component of the full eight-indicator pool has an eigenvalue of 2.72 and accounts for 34.0 percent of the total variance, against 15.2 percent for the second; across the five leave-one-out pools actually used, the first component accounts for between 32.7 and 37.6 percent. In the full pool and in every leave-one-out pool every loading is positive, ranging in the full pool from 0.22 for improved water to 0.45 for improved sanitation, with the durable-asset count at 0.42. S4 Table reports the eigenvalues, the share of variance explained and the loadings for the full pool, for each leave-one-out pool, for the asset-only pool and for the pre-revision pool of Sect 3.4.1. In the last of these the original housing binary loads at −0.28, the only loading of any size below zero in any pool (the conventional clean-fuel binary, which marks one household, loads at −0.02), which is the anomaly of Sect 3.1.2 seen from the other side.

The weighting matters and was not handled consistently in the first version of this work. Means, standard deviations and the correlation matrix from which the component is extracted are all computed with survey weights, as are the category means that later use the index. An unweighted anchor feeding weighted category means treats the design as relevant to one step and irrelevant to the previous one.

Leave-one-out removes mechanical circularity: a variable cannot help build the index against which it is judged. It does not make the exercise independent of the dataset on which the method was developed, and the paper does not claim that it does. Independence of that stronger kind is what Sects 3.2 and 3.3 are for.

#### 2.2.2 *Location adjustment.*

Wealth in this setting varies sharply between states and between urban and rural areas, and the composition of a category differs across those cells. The index is therefore residualised on urban/rural status and state by survey-weighted least squares, and category positions are computed on the residual. Four alternative specifications (state only, urban only, the full stratum interaction, and no adjustment) are reported in Sect 3.4.

#### 2.2.3 *The ordering diagnostic.*

For each category k, the weighted mean of the location-adjusted index is computed among households in that category. The categories are then compared pairwise. For every pair the difference in means is taken directly from the bootstrap distribution described below, and a confidence interval is reported for the difference itself. The non-overlap of two separate marginal intervals is not used as a test anywhere in this paper; it is not one.

A pair is recorded as a robust inversion when the category the coding places higher sits significantly lower on the wealth gradient, judged against the difference distribution. Because five variables generate a large family of pairwise comparisons, the false discovery rate across the entire family is controlled by the Benjamini–Hochberg procedure at q = 0.05 [16].

Two summary statistics accompany the pairwise results. The headline is a frequency-weighted discordance ratio: the share of category pairs, weighted by the product of the two categories’ population shares, whose wealth order contradicts the assumed order. This is reported alongside the weighted share of households sitting in a category involved in at least one robust inversion. Spearman’s rank correlation between the assumed and observed orderings is retained but demoted to a secondary statistic, because it weights a two-household category as heavily as a nine-hundred-household one and can therefore stay high while a populous inversion is present.

#### 2.2.4 *The cut-point diagnostic.*

Binary indicators are the form in which categorical variables usually reach applied work, and a binary can be misplaced even when the ordering behind it is sound. For a binary defined by a set S of categories, the diagnostic computes the weighted difference in location-adjusted wealth between households inside S and those outside it, with a bootstrap interval. A negative difference means the indicator is upside down: the households it labels adequate sit lower on the wealth gradient than the ones it excludes.

This diagnostic evaluates a cut that someone else has drawn. It does not propose a replacement, and it involves no threshold defined relative to the sample. That is a deliberate change from the first version of this work, in which categories whose adjusted mean exceeded the sample mean of zero were assembled into a “validated improved set.” That rule is indefensible in a population where roughly four households in five live below the international extreme-poverty line: being above the local average is not evidence of adequacy in any technical sense, and in these data the rule would have classified an open-water drinking source as improved. It has been removed rather than repaired.

#### 2.2.5 *The precision floor.*

Several categories in surveys of this kind contain a handful of households. A category with one household yields a bootstrap interval whose endpoints coincide. That is a point, not an interval, and it is the opposite of the signal that thin evidence should produce. A single rule is therefore fixed in advance and applied to every variable, every wave and every robustness run: a category is always reported, but is classified, and admitted to the pairwise family, only if it contains at least 30 households in at least 5 enumeration areas. Sect 3.4 reports what happens at three alternative floors.

#### 2.2.6 *Inference.*

Uncertainty comes from a stratified cluster bootstrap that mirrors the survey design [17]. Within each of the design strata, that stratum’s enumeration areas are resampled with replacement; households travel with their area. Sampling weights are carried through the resampling as multipliers: in each replication a household’s survey weight is multiplied by the number of times its enumeration area was drawn, so an area drawn twice counts twice and an area not drawn counts for nothing, and the weighted standardisation, principal component, residualisation and category means of that replication all use the product. The weights themselves are not re-estimated; the design that generated them is taken as fixed. Two features of the implementation deserve emphasis because both were wrong in the first version of this work.

First, enumeration areas are identified by the pair (stratum, area), not by the raw area identifier. In these files the area identifier is unique only within stratum: each of its 26 values recurs across three to seven different strata. Factorising it alone collapses a design of 156 enumeration areas into 26 pseudo-clusters, each pooling households drawn from several states, and resamples those.

Second, the entire estimation chain is re-estimated inside every replication: the standardisation, the principal component, the location residualisation and the category means. Fixing the index outside the loop and resampling only pre-computed aggregates treats the anchor as known when it is estimated, and understates uncertainty in every quantity that depends on it. The main results of Sects 3.1 and 3.2, and the alternative anchors of Sect 3.4.1, use 2,000 replications. The precision-floor and tolerance sensitivities of Sects 3.4.2 and 3.4.3 use 1,500; the 42-run robustness suite of Sect 3.4.1 uses 600 per run; and the simulation of Sect 3.3 uses 300 per synthetic dataset. Each count is fixed in the deposited run script, and the deposited outputs reproduce every number in this paper to the last decimal when it is re-run.

#### 2.2.7 *Reporting a negative result.*

Where no robust inversion is found, the paper reports that no robust evidence of an inversion was detected. It does not report that the coding has been cleared, confirmed or validated. The distinction is not pedantic: a variable can fail to produce a flag because its coding is sound, or because the categories concerned are too thin or the design too coarse for any inversion to have shown up.

Two devices are used to give the negative results content. Adjacent pairs are subjected to two one-sided tests [18] against a tolerance of 0.20 standard deviations, so that a pair whose difference interval lies wholly inside that band can be described as equivalent within tolerance rather than merely undetected. The tolerance needs a word of justification, because no external standard says what counts as a negligible difference in position on a wealth index. A fifth of a standard deviation is the conventional boundary below which a standardised difference is described as small [19]. It is also close to the resolution of the design in these data: the smallest adjacent step between categories above the precision floor that the main specification resolves as different from zero is 0.16 standard deviations, between stick-walled and mud-walled tukuls, so a tolerance much wider than 0.20 would begin to declare negligible differences that the design itself reports as real, which is what Sect 3.4.3 shows happening at 0.30. The value was fixed as a constant in the analysis code before the revised estimates were produced and was not derived from them; it was not, however, registered outside this study, and Sect 3.4.3 reports the neighbouring values as a check made afterwards. For that reason the equivalence tests are offered as a complementary sensitivity analysis rather than as the primary bound on the null. The primary bound comes from the simulation in Sect 3.3, which supplies power curves stating what size of inversion the design would have found at each category frequency. A negative result accompanied by neither device is reported as inconclusive, and several are.

### 2.3 Data

#### 2.3.1 *The survey and its design.*

The South Sudan High Frequency Survey was conducted by the World Bank with South Sudan’s National Bureau of Statistics, funded by the UK Department for International Development [20,21]. The main analysis uses Wave 3, collected between September 2016 and March 2017 across seven of the country’s ten former states: Western Equatoria, Central Equatoria, Eastern Equatoria, Northern Bahr-el-Ghazal, Western Bahr-el-Ghazal, Warrap and Lakes. The remaining three states could not be surveyed for security reasons.

The design is stratified and two-stage. Strata are formed by state crossed with urban/rural status, giving 14 strata. Within each stratum, enumeration areas are the primary sampling units and were drawn with probability proportional to size, estimated from the 2008 Census; within each selected area a listing was conducted and 12 households drawn at random. Sampling weights are the inverse probability of selection, decomposed into the area’s selection probability and the household’s selection probability within the area, and scaled to the number of households per stratum from the Census. Enumeration areas were replaced where security made fieldwork unfeasible, and households were replaced after three unsuccessful visits. The analysis sample contains 1,828–1,839 households depending on the variable under test, spread over 156 enumeration areas in 14 strata.

Waves 1 and 2 are used for the out-of-sample application in Sect 3.2. Wave 1 was collected between February and September 2015 and contains 3,550 households in 300 enumeration areas across 12 strata. Wave 2 was collected between February and June 2016, revisited the urban households of Wave 1 and added areas in four states, and contains 1,189 households in 101 enumeration areas.

#### 2.3.2 *Variables.*

Five ordinal variables are tested: dwelling structure, toilet facility, main drinking-water source, main lighting source and main cooking-fuel source. Four of these reach the analyst through derived variables supplied in the survey’s own clean output, which collapse the raw questionnaire responses into ordered categories. Dwelling structure is the exception: it is a plain ten-category question with no derived counterpart and no improved/unimproved split of any kind in the survey materials.

The anchor pool holds eight indicators: improved housing, improved water, improved sanitation, electricity for lighting, clean cooking, a count of durable asset types owned, room density, and land ownership. Every indicator is constructed in one place in the deposited code and nowhere else. For each variable under test, all indicators derived from that variable are removed from the pool.

#### 2.3.3 *Provenance of the improved-housing binary.*

Because the housing case is the paper’s principal application, and because a flag cannot be resolved without knowing where the classification came from, the survey documentation was examined directly. The finding is unambiguous and it changes how the case must be described.

The questionnaire item records the structure a household inhabits in ten labelled categories: tent, stick tukul, mud tukul, flat or apartment, villa, one-floor mud house, one-floor brick or concrete house, wooden house, multi-storey house, and incomplete structure. The survey defines no improved/unimproved split over these categories. The World Bank’s derived variables cover water, sanitation, lighting and cooking; there is no derived housing variable. Both the original improved set and the revised set used in earlier analysis of these data were therefore created during analysis, not supplied by the survey.

One detail of the coding is worth recording because it suggests a mechanism. The category codes run 1, 3, 4, 5, 6, 7, 8, 9, 10, 11; there is no code 2. A rule expressed as “types 1–3” therefore selects tents and stick tukuls, and nothing else: 478 households in the full Wave 3 file, 25.9 percent unweighted, and the two poorest dwelling types in the survey. (In the analysis sample used in Sect 3.1, which drops households with a missing anchor indicator, the same set holds 475 households and a survey-weighted 29.2 percent.) Many household survey codebooks list the most substantial dwelling first; this one lists the least substantial first. A rule imported from a survey with the opposite convention would select precisely this set. Under the terminology of Sect 2.1 this is a recoding error, located in the analysis rather than in the survey, and the paper describes it that way throughout.

#### 2.3.4 *Ethics statement.*

This study is a secondary analysis of de-identified, publicly archived household survey microdata obtained from the World Bank Microdata Library. The author collected no data from human participants and had no contact with respondents. The datasets are distributed as anonymised Public Use Datasets from which direct identifiers have been removed by the data producer. Because the research involved no interaction with human subjects and no access to identifiable private information, no institutional review board approval was required, and none was sought; Gambella University’s research ethics committee does not require review for secondary analysis of anonymised public-use data. Informed consent was obtained from participants by the original data collectors, the World Bank and South Sudan’s National Bureau of Statistics, at the time of the original surveys. The author accessed the data under the World Bank Microdata Library’s standard terms for public use datasets, which require agreement not to attempt re-identification of any respondent.

## 3 Results

### 3.1 The two diagnostics in Wave 3

#### 3.1.1 *The ordering diagnostic.*

Table 2 reports the ordering diagnostic for all five variables in Wave 3. No robust inversion survives in any of them. Across the five variables, 21 pairwise comparisons are admitted by the precision floor, and none is a significant inversion after control of the false discovery rate. Every pairwise comparison, whether or not it clears the floor, is listed with its difference, interval and adjusted p-value in S2 Table.

**Table 2:** Ordering diagnostic, Wave 3, main anchor.

Variable	N	Categories	Classifiable	Pairs tested	Robust inversions	Weighted discordance	Spearman ρ
Dwelling structure	1,838	10	4	6	0	0.012	0.84
Sanitation	1,828	4	2	1	0	0.003	0.80
Drinking water	1,839	4	3	3	0	0.016	0.80
Lighting	1,838	5	5	10	0	0.290	0.90
Cooking fuel	1,839	4	2	1	0	0.002	−0.20

Note. 2,000 stratified cluster-bootstrap replications; 156 enumeration areas within 14 strata; the full estimation chain re-estimated in every replication; Benjamini–Hochberg control at q = 0.05 across the whole family of pairwise comparisons. Spearman’s ρ is reported for comparability with earlier work and is not used to draw any conclusion.

Fig 1 shows the same result category by category. The point to take from it is the colour: in dwelling structure, every category the design can classify falls in ascending order, and every category out of order sits below the precision floor.

**Fig 1:**
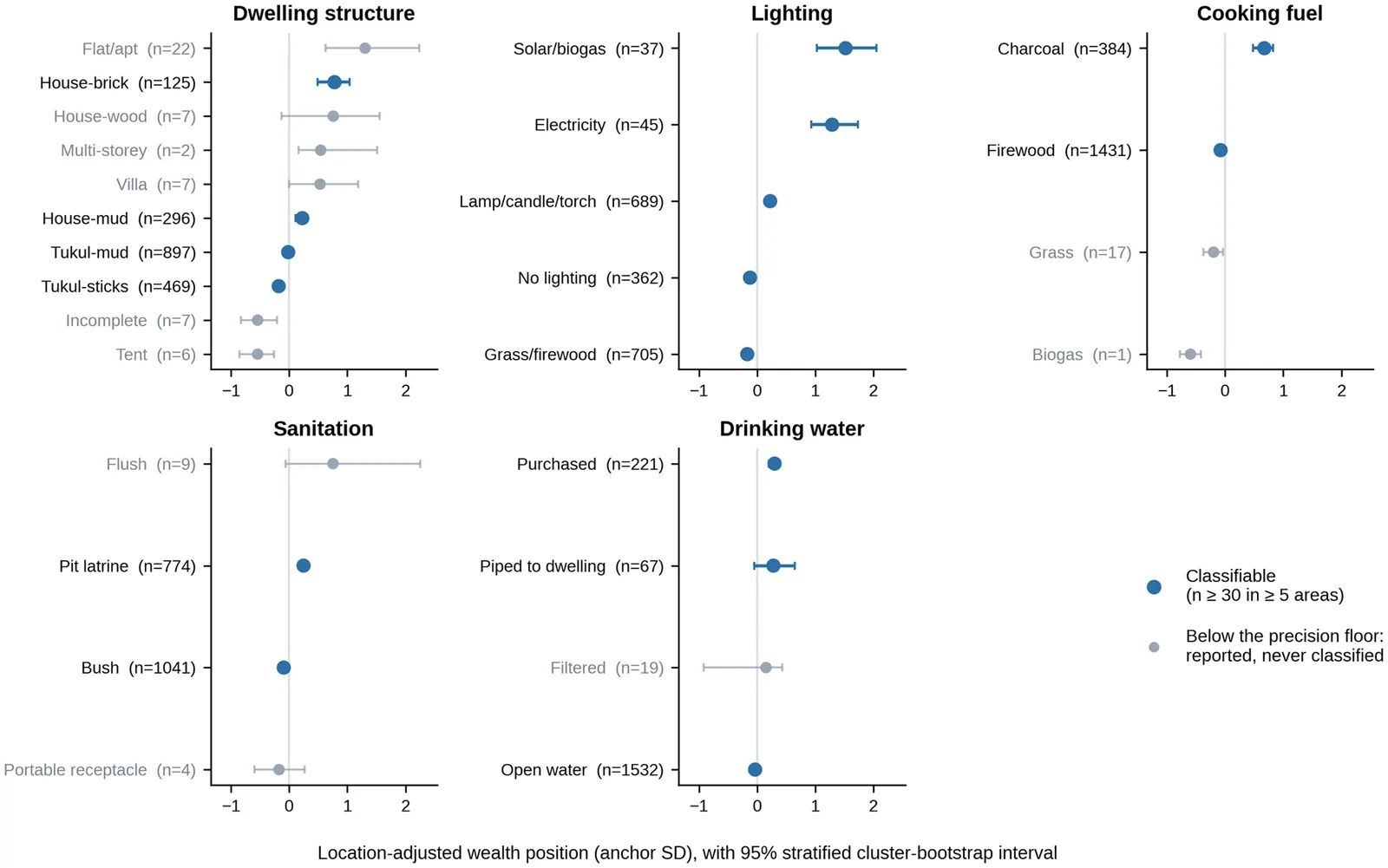
Location-adjusted wealth position of each response category, by variable, Wave 3. Markers give the weighted mean position of each category on the anchor, in standard-deviation units, with 95 percent stratified cluster-bootstrap intervals from 2,000 replications in which the entire estimation chain is re-estimated. Categories are ordered by observed wealth position, not by their assigned codes. Dark markers denote categories that clear the pre-specified precision floor of 30 households in at least 5 enumeration areas and are therefore admitted to the pairwise comparisons; pale markers denote categories that are reported but never classified. N = 1,828 to 1,839 depending on the variable.

Two features of Table 2 need comment. First, the dwelling-structure ordering is monotonic among the categories large enough to classify: stick tukul at −0.18, mud tukul at −0.02, mud house at +0.23, brick house at +0.78. The apparent disorder in the full ten-category ranking is carried entirely by categories holding between two and twenty-two households (flat or apartment, villa, wooden house, multi-storey house, tent and incomplete structure), for which the design supports no conclusion. Second, cooking fuel returns a Spearman ρ of −0.20, which looks alarming and means very little: it is computed over four categories, two of which contain seventeen and one households respectively. The frequency-weighted discordance ratio for the same variable is 0.002. This is exactly the contrast that motivates demoting ρ.

#### 3.1.2 *The cut-point diagnostic.*

Table 3 turns to the binaries. Five of the seven cuts examined place their labelled side higher on the wealth gradient than the households they exclude. One places it lower, and one cannot be estimated at all.

**Table 3:** Cut-point diagnostic, Wave 3, main anchor.

Binary indicator	n inside	Weighted share	Wealth gap (SD)	95% CI	Verdict
Improved housing, original analytical coding (types 1–3)	475	29.2%	−0.263	[−0.33, −0.17]	Inversion: labelled side sits lower
Improved housing, revised coding (types 5, 8, 9, 10)	156	4.4%	+0.881	[+0.60, +1.14]	Labelled side sits higher
Clean cooking fuel, conventional definition (gas, electricity, biogas)	1	0.0%	–	–	Not estimable
Cooking-fuel cut placed one category lower, at charcoal	385	10.4%	+0.749	[+0.53, +0.91]	Labelled side sits higher
Improved drinking water (derived variable)	307	11.0%	+0.324	[+0.22, +0.42]	Labelled side sits higher
Improved sanitation (derived variable)	783	27.2%	+0.345	[+0.24, +0.43]	Labelled side sits higher
Electricity for lighting (derived variable)	82	2.5%	+1.453	[+1.07, +1.84]	Labelled side sits higher

Note. The wealth gap is the weighted difference in location-adjusted wealth between households the binary labels adequate and all others, in anchor standard-deviation units. Estimates are withheld where the precision floor is not met. The verdict records only the sign and precision of the gap: it states where the labelled households sit on the local wealth gradient, and it does not certify that any binary’s boundary is technically, environmentally or health-wise correct.

The original improved-housing binary is inverted, decisively. The households it labels improved sit 0.26 standard deviations below those it excludes, with an interval nowhere near zero. The reason is visible in the composition: the labelled set consists of tents and stick tukuls. The revised coding used in later analysis of these data places its labelled side 0.88 standard deviations higher, which is what one would expect of a set consisting of flats, brick houses, wooden houses and multi-storey houses. The diagnostic, however, certifies that set no more than it certifies any other, and Sect 4 returns to what it does and does not license.

The conventional clean-fuel binary is the more instructive case. In Wave 3 it captures a single household. It is not that the indicator marks a small share of the population; it is that no estimate of its position is possible at the precision this paper requires of itself, and the honest report is that the cut cannot be evaluated. Meanwhile the one large step in the cooking-fuel variable sits a category lower. Moving the cut to charcoal produces a gap of +0.75 standard deviations, precisely estimated.

It bears repeating that this is not an argument that charcoal is a clean fuel. It is not, and the clean-fuel standard is not attempting to measure prosperity. The finding is that in this population the cooking-fuel variable’s information about relative wealth is concentrated at a boundary the clean-fuel indicator does not use, so an analyst who reaches for that indicator as a proxy for household living standards will capture almost nothing. Under the terminology of Sect 2.1 this is a context-dependent classification, and the appropriate response is to stop using the indicator as a wealth proxy, not to redefine clean cooking.

#### 3.1.3 *Where the gradient steps.*

The adjacent-step profile makes the point in Table 3 general. Along the assumed ordering of each variable, most adjacent steps are indistinguishable from zero and a few are large. In lighting, the step from grass and firewood to lamp, candle or torch is +0.39 and the step from there to electricity is +1.07, while the step from electricity to solar or biogas (+0.23) cannot be distinguished from zero and the step from no lighting to grass and firewood (−0.05) is equivalent within tolerance. In cooking fuel, the single step from firewood to charcoal is +0.75 and carries the variable’s entire wealth signal. In drinking water, the step from purchased to piped supply is −0.02 and is simply inconclusive.

This is a useful output in its own right, within a limit that should be stated. An analyst choosing where to cut a categorical variable for a wealth-related purpose, such as an input to a relative wealth index, can read the steps directly rather than importing a boundary drawn for a different purpose in a different setting. What the steps cannot do is fix a normative boundary. That a large step separates charcoal from firewood says where the cooking-fuel variable carries its wealth information; it says nothing about whether charcoal is clean, and an indicator whose purpose is to measure adequacy, improvement or safety must take its boundary from the technical or health standard that defines those terms, not from the gradient.

### 3.2 Out-of-sample application

The method was developed after an anomaly was noticed in Wave 3, and that same anomaly cannot then serve as confirmation that the method works. The estimator was therefore applied, without modification, to two waves that played no part in its development.

Two constraints are recorded rather than worked around. Wave 1 does not contain the dwelling-structure question, so the housing tests run on Wave 2 only. Wave 2’s public file carries neither a stratum variable nor an urban indicator, so its bootstrap is stratified by state and its location adjustment uses state alone. Table 4 reports the cut-point diagnostic for every binary in both waves.

**Table 4:** Cut-point diagnostic out of sample.

Wave	Binary indicator	n inside	Wealth gap (SD)	95% CI	Verdict
2	Improved housing, original analytical coding	241	−0.277	[−0.41, −0.10]	Inversion: labelled side sits lower
2	Improved housing, revised coding	178	+0.713	[+0.54, +0.87]	Labelled side sits higher
1	Clean cooking fuel, conventional definition	0	–	–	Not estimable
2	Clean cooking fuel, conventional definition	3	–	–	Not estimable
1	Cooking-fuel cut at charcoal	947	+0.512	[+0.31, +0.68]	Labelled side sits higher
2	Cooking-fuel cut at charcoal	668	+0.663	[+0.49, +0.77]	Labelled side sits higher
1	Improved sanitation	1,623	+0.404	[+0.26, +0.52]	Labelled side sits higher
2	Improved sanitation	859	+0.620	[+0.42, +0.74]	Labelled side sits higher
1	Electricity for lighting	205	+0.983	[+0.71, +1.19]	Labelled side sits higher
2	Electricity for lighting	99	+1.033	[+0.69, +1.39]	Labelled side sits higher
1	Improved drinking water	342	+0.394	[−0.13, +0.66]	Inconclusive
2	Improved drinking water	95	+0.189	[−0.20, +0.56]	Inconclusive

Note. Wave 1 does not contain the dwelling-structure question, so the two housing rows appear for Wave 2 only. Wave 2’s public file carries neither a stratum variable nor an urban indicator, so its bootstrap is stratified by state and its location adjustment uses state alone; the Wave 2 rows are therefore not an identical replication of the Wave 3 procedure. Verdicts have the meaning given in the note to Table 3.

The housing inversion replicates. Wave 2 returns −0.277 standard deviations, against −0.263 in Wave 3: the same sign and effectively the same magnitude, in a wave the method never saw. The failure of the conventional clean-fuel binary replicates too, and more starkly: it captures no households at all in Wave 1 and three in Wave 2. The water cut is inconclusive in both waves, which is a limitation of the data rather than a finding about the indicator.

Fig 2 assembles the cut-point diagnostic for every binary, in every wave in which it can be evaluated. One indicator sits alone to the left of zero, and it does so in both of its waves.

**Fig 2:**
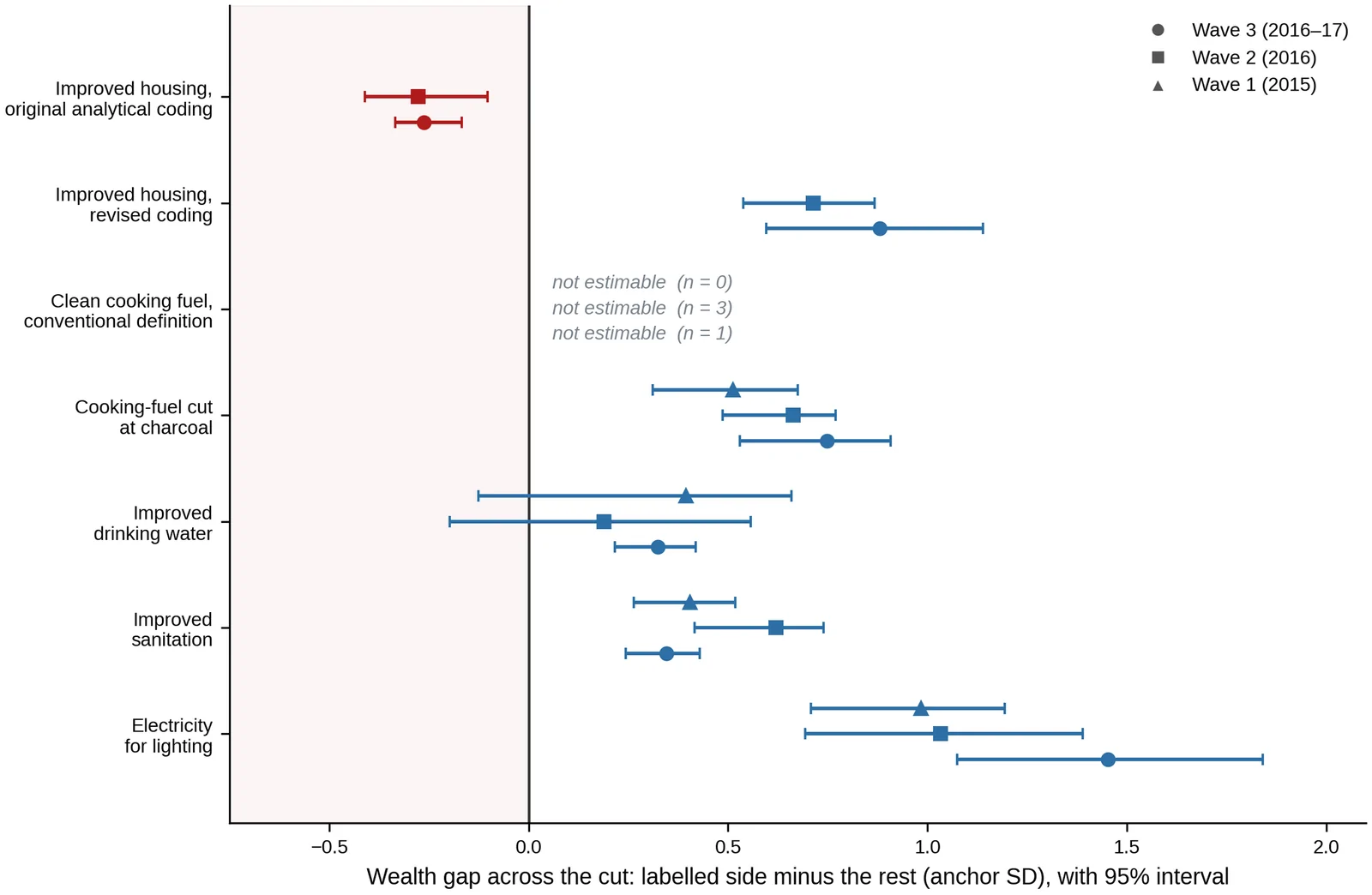
A binary whose labelled side sits left of zero is upside down. Each marker gives the weighted difference in location-adjusted wealth between the households a binary indicator labels adequate and all other households, in anchor standard-deviation units, with a 95 percent stratified cluster-bootstrap interval. A binary whose interval lies to the left of zero is inverted: the households it labels adequate sit lower on the wealth gradient than those it excludes. The improved-housing binary built during earlier analysis of these data is the only such case, and it appears in both waves in which it can be evaluated. Circles, squares and triangles mark Waves 3, 2 and 1 respectively, and the shaded region to the left of zero marks the inverted side. The conventional clean-fuel binary cannot be estimated in any wave, capturing between zero and three households. Wave 1 does not contain the dwelling-structure question.

The ordering diagnostic agrees across waves everywhere except one pair (S3 Table lists every out-of-sample comparison), and that exception is reported here rather than smoothed away. In Wave 2 the lighting variable shows one robust inversion: households reporting no lighting at all sit above those using grass or firewood (−0.20 against −0.78, adjusted p = 0.008), where the coding places them below. The same pair runs in the same direction in Wave 3 (−0.12 against −0.17), but the gap there is small enough to be declared equivalent within tolerance. Consistent direction across waves, differing magnitude, differing verdict. Sect 3.4 shows the same pair crossing the threshold in Wave 3 under several perturbations of the survey-indicator anchor, and reversing sign under the consumption anchor. The safest reading is that these data do not resolve the pair, and that a purpose-built instrument would be needed to do so. The case also fixes how a user of WACV should treat a finding of this kind. A flag that appears under some anchors, waves or specifications and not others is a candidate for investigation, not an inversion; the report should list the specifications under which it appears and those under which it does not, and it licenses no recoding. The Wave 2 lighting pair is reported as a robust inversion within that wave, because that is what the pre-specified rule returns there, and as unresolved overall, because the same rule returns the opposite verdict in Wave 3 and under the consumption anchor; the overall verdict is the one a user should act on.

### 3.3 Simulation

An out-of-sample wave establishes that the procedure behaves consistently. It cannot establish that the procedure recovers an inversion that is genuinely present, because in real data the truth is unknown. For that, a synthetic population was constructed in which the ordering is known by construction and an inversion of known size is planted between a known pair of categories.

The population reproduces the survey’s design features: 14 strata, clustered enumeration areas of twelve households, unequal sampling weights, and anchor indicators generated as noisy functions of a latent wealth variable together with an urban location effect that the residualisation is meant to remove. The grid varies the sample size, the population share of the category carrying the planted inversion, and the size of the inversion in standard-deviation units. Each of the 32 cells was replicated 150 times with 300 bootstrap replications, for 4,800 simulated datasets in total. Because a large planted swap can also invert that category against others, those additional genuine inversions are scored as true rather than false; scoring them as false positives would flatter the procedure’s specificity. Table 5 reports the full grid.

**Table 5:** Simulation: detection of planted category inversions.

N	Category share	Mean category n	False positives at Δ = 0	Power at Δ = 0.2	Δ = 0.4	Δ = 0.8	Δ at 80% power
1,800	2%	36	0.045	0.20	0.52	0.98	0.65
1,800	5%	90	0.040	0.31	0.79	1.00	0.41
1,800	10%	180	0.020	0.53	0.97	1.00	0.32
1,800	25%	450	0.033	0.61	0.99	1.00	0.30
3,550	2%	71	0.020	0.29	0.79	1.00	0.41
3,550	5%	177	0.033	0.57	0.97	1.00	0.32
3,550	10%	356	0.027	0.77	1.00	1.00	0.23
3,550	25%	886	0.060	0.91	1.00	1.00	0.17

Note. Δ is the size of the planted inversion in anchor standard-deviation units. The false-positive column reports how often the planted pair itself is flagged when its displacement is zero. Specificity is computed over every other testable pair, whose order is concordant by construction; it was 1.000 in every cell, so no such pair was flagged in any of the 4,800 datasets. The final column interpolates the smallest inversion the design recovers in four runs out of five.

Fig 3 traces the resulting power curves.

**Fig 3:**
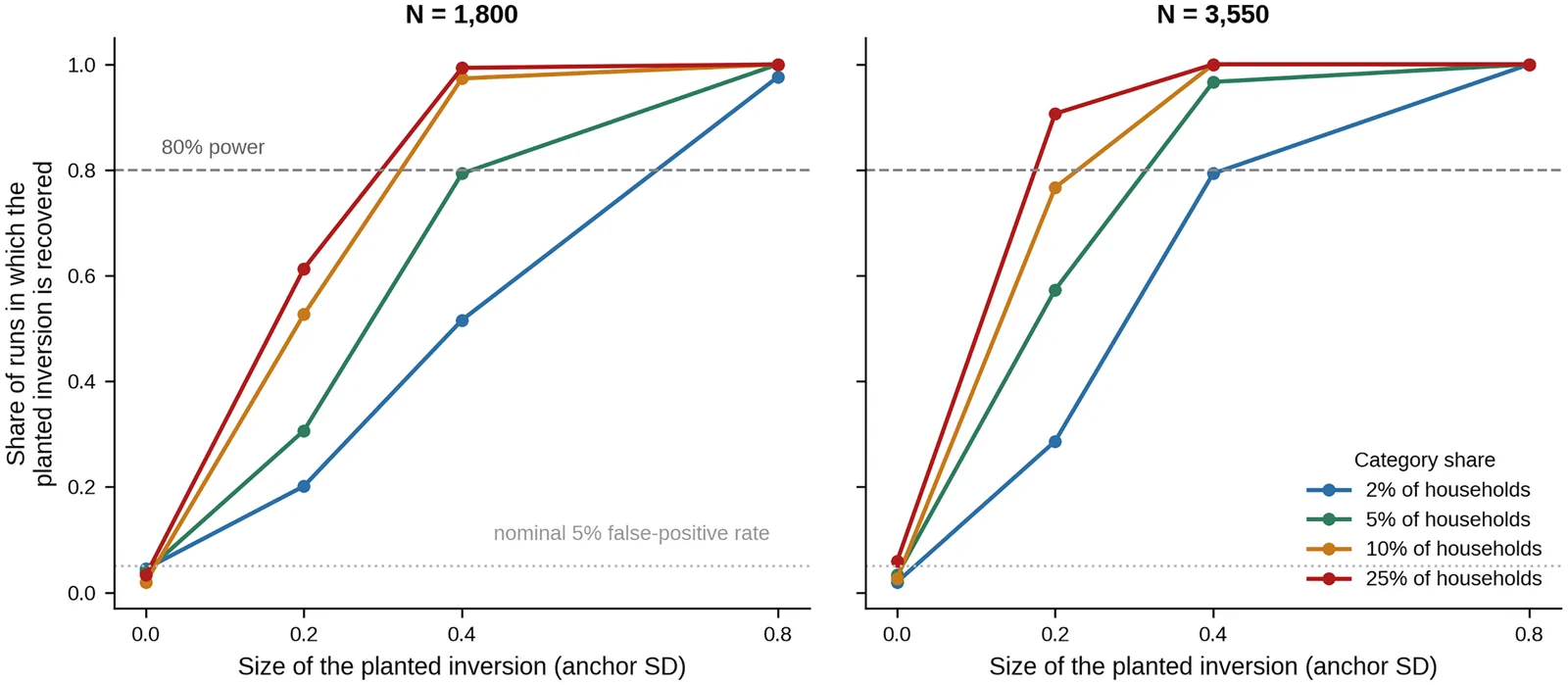
Power to recover a planted category inversion, by sample size, category frequency and inversion size. Each point is the share of 150 simulated datasets in which the procedure recovers an inversion planted between a known pair of categories, after Benjamini–Hochberg control across the pairwise family. The dashed line marks 80 percent power; the dotted line marks the nominal 5 percent false-positive rate, against which the points at zero inversion size should be read. Specificity, computed over every testable pair other than the planted one, was 1.000 in every cell: across all 4,800 simulated datasets, no such pair was ever flagged.

Three things follow. The procedure is correctly calibrated: with no inversion planted it flags the target pair between 2.0 and 6.0 percent of the time across the eight cells, against a nominal 5 percent, and the pooled rate over all cells is 3.5 percent. It is highly specific over the remaining pairs: across 4,800 datasets it never once flagged a pair other than the planted one. And its power depends, as one would expect, on how many households sit in the categories being compared. At a sample the size of Wave 3, an inversion of roughly 0.30 standard deviations is recovered four times in five for a category holding a quarter of households, rising to about 0.65 standard deviations for a category holding two percent.

This is what allows the null results of Sect 3.1 to be stated as something more than an absence of evidence. At Wave 3’s size, an ordering inversion smaller than about a third of a standard deviation in the common categories, or two-thirds in the thin ones, would plausibly have escaped detection. Inversions larger than that would most likely not have. The negative result is bounded, and the bound is reported rather than asserted.

### 3.4 Robustness and analysis choices

#### 3.4.1 *Anchor and specification.*

Forty-two robustness runs were carried out and are reported in full in S1 Table: each of the eight anchor indicators dropped in turn; every pair of them dropped in turn; five location-adjustment specifications; and an iterated second pass in which any anchor indicator whose source variable showed a robust inversion on the first pass would be rebuilt from the observed wealth ordering and the analysis re-run.

Thirty-seven of the forty-two reproduce every baseline verdict exactly. All eight drop-one runs pass. All five location specifications pass, including no location adjustment at all. The iterated second pass is a no-op, because no variable showed a robust inversion on the first pass and so no anchor indicator required rebuilding; this is reported as a fact about this application rather than as a check that happened to pass. The five drop-two runs that depart from the baseline all do so in the same way: the lighting pair discussed in Sect 3.2 crosses the significance threshold when the anchor is reduced to six indicators. No other verdict moves anywhere in the suite. Under the consumption anchor introduced below, that same pair runs the other way, with grass and firewood above no lighting by 0.23 standard deviations (adjusted p = 0.004), which is the order the coding assumes; this is the strongest reason not to read the Wave 2 result as a feature of the population.

Three further anchors address the concern that a pool of service indicators drawn from the same survey may share a common local structure that reinforces itself. The first uses only durable assets, room density and land ownership, and so contains no normatively coded service indicator at all. The second, the pre-revision anchor, substitutes the original housing coding and the conventional clean-fuel coding for the revised versions, so that no indicator adjusted in the light of these data enters the pool. The third leaves the asset and service block altogether: it is the logarithm of the survey’s own per-capita daily consumption aggregate, standardised and location-adjusted in the same way as the composite index, and it is the external measure of well-being these files contain. Because it is a single variable rather than a composite, leave-one-out is vacuous for it; and because consumption is measured with recall error over a short reference period, its correlation with the durable-asset count is only 0.38, so it is a noisier yardstick than the other three.

All three reproduce the ordering verdicts of Table 2: no robust inversion in any variable on any anchor. Table 6 reports the cut-point diagnostic under each. The asset-only and pre-revision anchors reproduce Table 3 in every row, and the housing inversion is if anything sharper under them, at −0.33 and −0.30 standard deviations. Under the consumption anchor the gaps are attenuated, as a noisier anchor implies, and the attenuation is close to uniform: the revised housing binary, the charcoal cut, the sanitation binary and the electricity binary all fall to between 43 and 52 percent of their main-anchor values, and only the drinking-water gap, at 0.31 against 0.32, is left where it was. The original housing binary keeps its sign, at −0.10, but its interval reaches zero, so under the reporting rule of Sect 2.2.7 it is inconclusive on this anchor rather than an inversion. The direction of the anomaly is therefore consistent across all four anchors; its statistical clarity is not, and that is stated here rather than left to be found. Two readings of the loss are possible, and the pattern favours the first. If consumption is simply a noisier measure of the same latent wealth, every gap measured against it should shrink by a similar proportion, and the original housing binary, at 39 percent of its main-anchor value, sits at the lower edge of the band the other binaries occupy rather than apart from it. If instead the housing result were sensitive to how the anchor is constructed, the housing gaps would move while the others held, which is not what Table 6 shows. The consumption anchor’s low correlation with the durable-asset count, noted above, points the same way. Measurement noise in the anchor is therefore the more plausible explanation, but the two cannot be separated with one consumption measure, and the precision of the housing inversion under this anchor is lost either way.

**Table 6:** Cut-point diagnostic under four anchors, Wave 3.

Binary indicator	Main anchor	Asset-only anchor	Pre-revision anchor	Consumption anchor
Improved housing, original analytical coding	−0.26 [−0.33, −0.17]	−0.33 [−0.46, −0.19]	−0.30 [−0.38, −0.19]	−0.10 [−0.24, + 0.01]
Improved housing, revised coding	+0.88 [+0.60, + 1.14]	+0.58 [+0.35, + 0.78]	+0.84 [+0.58, + 1.09]	+0.43 [+0.30, + 0.56]
Clean cooking fuel, conventional definition	–	–	–	–
Cooking-fuel cut at charcoal	+0.75 [+0.53, + 0.91]	+0.59 [+0.30, + 0.81]	+0.70 [+0.46, + 0.86]	+0.39 [+0.20, + 0.54]
Improved drinking water	+0.32 [+0.22, + 0.42]	+0.21 [+0.08, + 0.32]	+0.25 [+0.13, + 0.36]	+0.31 [+0.20, + 0.41]
Improved sanitation	+0.35 [+0.24, + 0.43]	+0.25 [+0.17, + 0.32]	+0.29 [+0.20, + 0.36]	+0.17 [+0.10, + 0.23]
Electricity for lighting	+1.45 [+1.07, + 1.84]	+1.25 [+0.99, + 1.53]	+1.07 [+0.83, + 1.30]	+0.63 [+0.44, + 0.82]

Note. Each cell is the weighted difference in location-adjusted wealth between households the binary labels adequate and all others, in the standard-deviation units of the anchor named in the column, with a 95 percent stratified cluster-bootstrap interval from 2,000 replications. The conventional clean-fuel binary captures one household and is not estimable on any anchor. The consumption anchor is the standardised logarithm of per-capita daily consumption; it is available in Wave 3 only. The pre-revision anchor is labelled precorrection in the deposited code.

#### 3.4.2 *The precision floor.*

The floor of 30 households and 5 enumeration areas is the one analysis choice that could plausibly drive the null in Table 2, since it excludes the categories in which the apparent housing disorder sits. Table 7 reports what happens at three alternatives.

**Table 7:** Sensitivity to the precision floor, Wave 3, all five variables.

Floor	Categories classifiable	Pairs tested	Robust inversions
n ≥ 50 and 8 areas	14	14	0
n ≥ 30 and 5 areas (adopted)	16	21	0
n ≥ 20 and 3 areas	17	25	0
No floor	27	73	4

Note. The adopted floor is the middle case. Lowering it to 20 households admits the flat-or-apartment category, which contains 22, and changes no verdict.

The null is unchanged at every floor except no floor at all. Lowering the threshold to 20 households admits the flat-or-apartment category, the largest of the categories the adopted floor excludes, and still produces no robust inversion. The four inversions that appear when the floor is removed entirely rest on categories containing between one and seven households, including one containing a single household whose bootstrap interval collapses to a point. The choice of floor is therefore not what produces the result.

#### 3.4.3 *The equivalence tolerance.*

The tolerance of 0.20 standard deviations governs which non-findings may be described as equivalent rather than inconclusive. It was fixed before the revised estimates were run, for the reasons given in Sect 2.2.7, and this section reports what the neighbouring values would have done; the check was made after the results were known and is presented as a sensitivity analysis, not as the ground on which the value was chosen. At 0.10 no pair qualifies. At 0.30 two qualify, but one of them is also significantly different from zero, so that a reader would be told simultaneously that a difference exists and that it is negligible. At 0.20 exactly one pair qualifies and the two verdicts remain disjoint. The pre-specified value therefore also happens to be the largest that keeps the reporting coherent, and it is applied uniformly.

## 4 Discussion

### 4.1 What the application shows

In this survey no robust evidence of an ordering inversion is found in any of the five variables tested in the main specification, within the limits the equivalence tests and the power analysis make explicit. That is the main empirical result, and it is not a disappointing one: a diagnostic that flagged everything would be worthless, and the value of the exercise lies in its being able to return a null that is bounded by a power calculation rather than merely asserted.

The failure is at a cut-point rather than in an ordering, and it is severe. An improved-housing binary constructed during analysis of these data assigns the improved label to the two poorest dwelling types in the survey, and the resulting indicator points the wrong way by a quarter of a standard deviation in two independent waves and under three of the four anchors tried; the fourth, consumption, keeps the sign but not the precision. Any regression using it as a control, any wealth index containing it, and any deprivation count built on it would be affected [9,10], and in the direction that makes housing appear to be negatively associated with wealth.

The provenance audit determines how this should be read. Because the survey defines no improved-housing split at all, the error belongs to the analysis rather than to the data: a recoding error in the terms of Sect 2.1. That makes it a smaller claim about survey quality and a larger one about analytical practice: the coding was plausible, undocumented, never checked against the data, and wrong, and none of those four properties is unusual.

### 4.2 What the paper does not claim

The revised housing coding is not certified here. The cut-point diagnostic reports that its labelled side sits higher on the gradient; it does not establish that its boundary is the right one, and an alternative set including villa, a category the revised set omits even though it sits at +0.53 on the wealth gradient, would also place its labelled side higher. Choosing between them requires a substantive criterion about dwelling construction, not a wealth comparison, and this paper does not supply one.

Nor does the paper claim that any international service definition is incorrect. The clean-fuel result says the indicator carries almost no information about relative wealth in this population, which is a statement about its usefulness as a proxy, not about its validity as a measure of what it actually measures. The same limit applies to every binary in Table 3. WACV locates a category on the local wealth gradient and nothing more; it does not establish, and cannot, whether a category is technically, environmentally or health-wise improved. Those are questions for the standard that defines the term, and charcoal, which sits high on this gradient and is not a clean fuel, is the standing reminder of the difference.

### 4.3 Limitations

Five are worth stating. The evidence is from one survey programme in one country. Three waves show that the procedure can be applied and that its principal finding replicates; they cannot establish how common recoding errors of this kind are across fragile-state surveys, and application to other survey programmes and countries is the natural next step. The out-of-sample waves are also not an identical replication setting: Wave 2’s public file carries neither a stratum variable nor an urban indicator, so its bootstrap is stratified by state alone and its location adjustment uses state alone, and Wave 1 lacks the dwelling-structure question altogether; cross-wave consistency should be read with those differences in mind. The anchor is a wealth index built from indicators collected in the same instrument, so a systematic distortion affecting many indicators at once would not be detected by any leave-one-out design; the asset-only anchor mitigates this, and the consumption anchor steps outside the asset and service block altogether, but consumption is the noisiest of the four yardsticks, and two other candidate external measures, education and employment, were not tried, because neither is a measure of living standards in the sense the wealth index is meant to capture. The precision floor means that in a survey of this size the procedure is effectively silent about categories holding fewer than about thirty households, which in practice includes most of the categories at the top of a dwelling-structure distribution in a very poor country. And the cut-point diagnostic requires a binary to exist before it can be evaluated; it offers nothing to an analyst constructing one from scratch, beyond the adjacent-step profile of Sect 3.1.3.

### 4.4 Practical recommendation

Before a categorical variable is used to build a wealth index, or before a binary derived from one enters a deprivation count, three questions are worth asking, and all three can be answered with the data already in hand. Did this coding come from the instrument, from the codebook, or from an analyst? Do the categories sit on the local wealth gradient in the order the coding assumes, with the design honoured and the anchor’s own uncertainty carried through? And does the binary’s boundary fall where the gradient actually steps? The procedure set out here answers the second and third in a few minutes of computation. The first, which is the one that resolved the case examined in this paper, requires only that somebody read the codebook. Two further rules follow from the application. A minimum category size should be fixed before the procedure is run, applied to every variable and every specification, and reported with the results; the floor of 30 households in 5 enumeration areas used here is a reasonable default for a survey of this size, and Sect 3.4.2 shows how to report the sensitivity to it. And a flag that appears under some anchors, waves or specifications but not others should be reported as unresolved, with the specifications under which it appears listed, rather than as an inversion. It is a reason to look, and never a licence to recode.

## 5 Conclusion

Categorical orderings and the binaries built on them are load-bearing in welfare measurement and are seldom tested. Wealth-Anchored Category Validation tests them against the wealth gradient implied by the rest of a survey, and reports what it finds as a diagnostic rather than a verdict. Applied to three waves of the South Sudan High Frequency Survey, it finds no robust evidence of ordering inversions in the main specification, within the limits made explicit by the equivalence tests and power analysis, and finds one binary, constructed during analysis rather than supplied by the survey, that points the wrong way in every wave in which it can be evaluated. The general lesson is not that survey categories are unreliable. It is that a cut-point imported from one setting into another is an empirical claim about that setting, and can be checked before it is trusted.

## Supporting information

S1 Table Robustness suite, Wave 3. Number of robust inversions per variable under the baseline and 42 alternative specifications: each anchor indicator dropped in turn, every pair dropped in turn, five location-adjustment specifications, and the iterated second pass.
DOCX

S2 Table All pairwise category comparisons, Wave 3, under the main, asset-only, pre-revision and consumption anchors. For each pair: households in each category, precision-floor status, difference in location-adjusted wealth, 95 percent stratified cluster-bootstrap interval, bootstrap p-value, Benjamini–Hochberg adjusted p-value, and the equivalence and robust-inversion flags.
DOCX

S3 Table All pairwise category comparisons in Waves 1 and 2, main anchor, with the same columns as S2 Table. DOCX

S4 Table Principal-components anchor, Wave 3. Eigenvalues, share of variance explained by the first and second components, and first-component loadings for the full eight-indicator pool, for each of the five leave-one-out pools used in the main analysis, for the asset-only pool and for the pre-revision pool.
DOCX
