# Supplementary material for: Wealth-anchored category validation: A diagnostic for categorical orderings in fragile-state household surveys
Source: PLoS One. 2026 Oct 29;21(10):e0360752. doi: 10.1371/journal.pone.0360752 (PMC13718975; doi:10.1371/journal.pone.0360752)
Supplement: Supplementary file 1 [file pone.0360752.s001.docx]

**S1 Table. Robustness suite, Wave 3: number of robust inversions per variable under the baseline and 42 alternative specifications.**

Each row is one full re-run of the ordering diagnostic for all five variables (600 stratified cluster-bootstrap replications per run, Benjamini–Hochberg control at q = 0.05 across the whole family of pairwise comparisons, precision floor of 30 households in 5 enumeration areas). Cells give the number of robust inversions detected. Drop-one and drop-two rows remove the named indicator(s) from the main anchor pool; location rows vary the residualisation design; the iterated row rebuilds any anchor indicator whose source variable showed a robust inversion on the first pass (none did). The five drop-two runs showing a value of 1 under Lighting all concern the same pair, no lighting versus grass and firewood, discussed in Sections 6 and 8.1 of the main text.

| **Exercise** | **Specification** | **Dwelling** | **Sanitation** | **Water** | **Lighting** | **Cooking** |
| --- | --- | --- | --- | --- | --- | --- |
| baseline | main anchor, urban+state | 0 | 0 | 0 | 0 | 0 |
| drop-one | without I_housing | 0 | 0 | 0 | 0 | 0 |
| drop-one | without I_water | 0 | 0 | 0 | 0 | 0 |
| drop-one | without I_sanitation | 0 | 0 | 0 | 0 | 0 |
| drop-one | without I_electricity | 0 | 0 | 0 | 0 | 0 |
| drop-one | without I_cooking | 0 | 0 | 0 | 0 | 0 |
| drop-one | without I_assets | 0 | 0 | 0 | 0 | 0 |
| drop-one | without I_rooms | 0 | 0 | 0 | 0 | 0 |
| drop-one | without I_land | 0 | 0 | 0 | 0 | 0 |
| drop-two | without I_housing + I_water | 0 | 0 | 0 | 0 | 0 |
| drop-two | without I_housing + I_sanitation | 0 | 0 | 0 | 1 | 0 |
| drop-two | without I_housing + I_electricity | 0 | 0 | 0 | 0 | 0 |
| drop-two | without I_housing + I_cooking | 0 | 0 | 0 | 0 | 0 |
| drop-two | without I_housing + I_assets | 0 | 0 | 0 | 0 | 0 |
| drop-two | without I_housing + I_rooms | 0 | 0 | 0 | 0 | 0 |
| drop-two | without I_housing + I_land | 0 | 0 | 0 | 1 | 0 |
| drop-two | without I_water + I_sanitation | 0 | 0 | 0 | 0 | 0 |
| drop-two | without I_water + I_electricity | 0 | 0 | 0 | 0 | 0 |
| drop-two | without I_water + I_cooking | 0 | 0 | 0 | 0 | 0 |
| drop-two | without I_water + I_assets | 0 | 0 | 0 | 0 | 0 |
| drop-two | without I_water + I_rooms | 0 | 0 | 0 | 0 | 0 |
| drop-two | without I_water + I_land | 0 | 0 | 0 | 0 | 0 |
| drop-two | without I_sanitation + I_electricity | 0 | 0 | 0 | 0 | 0 |
| drop-two | without I_sanitation + I_cooking | 0 | 0 | 0 | 0 | 0 |
| drop-two | without I_sanitation + I_assets | 0 | 0 | 0 | 0 | 0 |
| drop-two | without I_sanitation + I_rooms | 0 | 0 | 0 | 1 | 0 |
| drop-two | without I_sanitation + I_land | 0 | 0 | 0 | 1 | 0 |
| drop-two | without I_electricity + I_cooking | 0 | 0 | 0 | 0 | 0 |
| drop-two | without I_electricity + I_assets | 0 | 0 | 0 | 0 | 0 |
| drop-two | without I_electricity + I_rooms | 0 | 0 | 0 | 0 | 0 |
| drop-two | without I_electricity + I_land | 0 | 0 | 0 | 0 | 0 |
| drop-two | without I_cooking + I_assets | 0 | 0 | 0 | 0 | 0 |
| drop-two | without I_cooking + I_rooms | 0 | 0 | 0 | 0 | 0 |
| drop-two | without I_cooking + I_land | 0 | 0 | 0 | 0 | 0 |
| drop-two | without I_assets + I_rooms | 0 | 0 | 0 | 0 | 0 |
| drop-two | without I_assets + I_land | 0 | 0 | 0 | 0 | 0 |
| drop-two | without I_rooms + I_land | 0 | 0 | 0 | 1 | 0 |
| location | urban+state | 0 | 0 | 0 | 0 | 0 |
| location | state | 0 | 0 | 0 | 0 | 0 |
| location | urban | 0 | 0 | 0 | 0 | 0 |
| location | stratum | 0 | 0 | 0 | 0 | 0 |
| location | none | 0 | 0 | 0 | 0 | 0 |
| iterated | second pass (no-op) | 0 | 0 | 0 | 0 | 0 |
