# Supplementary material for: Wealth-anchored category validation: A diagnostic for categorical orderings in fragile-state household surveys
Source: PLoS One. 2026 Oct 29;21(10):e0360752. doi: 10.1371/journal.pone.0360752 (PMC13718975; doi:10.1371/journal.pone.0360752)
Supplement: Supplementary file 2 [file pone.0360752.s002.docx]

**S2 Table. All pairwise category comparisons, Wave 3, under the main, asset-only, pre-revision and consumption anchors.**

Difference is the weighted mean of the location-adjusted anchor in the category the coding places higher minus that in the category it places lower, in anchor standard-deviation units, taken directly from the bootstrap distribution of the difference (2,000 stratified cluster-bootstrap replications, the whole estimation chain re-estimated in each). A negative difference with an adjusted p below 0.05 is a robust inversion. Adjusted p is Benjamini–Hochberg across the whole family of pairs within an anchor; it is blank for pairs below the precision floor, which are reported but never classified. Equivalent means both one-sided tests against a tolerance of 0.20 standard deviations reject. The consumption anchor is the standardised logarithm of per-capita daily consumption (Section 8.1). The pre-revision anchor substitutes the original housing coding and the conventional clean-fuel coding for the revised versions; it is labelled precorrection in the deposited code.

| **Anchor** | **Variable** | **Lower category (assigned)** | **Higher category (assigned)** | **n lower** | **n higher** | **Both above floor** | **Difference (SD)** | **95% CI** | **p** | **Adjusted p** | **Equivalent** | **Robust inversion** |
| --- | --- | --- | --- | --- | --- | --- | --- | --- | --- | --- | --- | --- |
| Main | Dwelling structure | Tent | Tukul-sticks | 6 | 469 | no | 0.364 | [0.086, 0.694] | 0.022 | – | no | no |
| Main | Dwelling structure | Tent | Tukul-mud | 6 | 897 | no | 0.527 | [0.243, 0.844] | 0.002 | – | no | no |
| Main | Dwelling structure | Tent | Incomplete | 6 | 7 | no | 0.001 | [−0.405, 0.493] | 0.921 | – | no | no |
| Main | Dwelling structure | Tent | House-mud | 6 | 296 | no | 0.769 | [0.459, 1.099] | 0.001 | – | no | no |
| Main | Dwelling structure | Tent | Villa | 6 | 7 | no | 1.075 | [0.458, 1.791] | 0.002 | – | no | no |
| Main | Dwelling structure | Tent | Flat/apt | 6 | 22 | no | 1.850 | [1.188, 2.703] | 0.001 | – | no | no |
| Main | Dwelling structure | Tent | House-brick | 6 | 125 | no | 1.324 | [0.919, 1.702] | 0.001 | – | no | no |
| Main | Dwelling structure | Tent | House-wood | 6 | 7 | no | 1.303 | [0.376, 2.117] | 0.007 | – | no | no |
| Main | Dwelling structure | Tent | Multi-storey | 6 | 2 | no | 1.085 | [0.600, 2.112] | 0.001 | – | no | no |
| Main | Dwelling structure | Tukul-sticks | Tukul-mud | 469 | 897 | yes | 0.164 | [0.071, 0.242] | 0.001 | 0.001 | no | no |
| Main | Dwelling structure | Tukul-sticks | Incomplete | 469 | 7 | no | −0.363 | [−0.668, −0.020] | 0.039 | – | no | no |
| Main | Dwelling structure | Tukul-sticks | House-mud | 469 | 296 | yes | 0.406 | [0.244, 0.523] | 0.001 | 0.001 | no | no |
| Main | Dwelling structure | Tukul-sticks | Villa | 469 | 7 | no | 0.711 | [0.151, 1.351] | 0.014 | – | no | no |
| Main | Dwelling structure | Tukul-sticks | Flat/apt | 469 | 22 | no | 1.486 | [0.786, 2.407] | 0.001 | – | no | no |
| Main | Dwelling structure | Tukul-sticks | House-brick | 469 | 125 | yes | 0.960 | [0.637, 1.225] | 0.001 | 0.001 | no | no |
| Main | Dwelling structure | Tukul-sticks | House-wood | 469 | 7 | no | 0.939 | [0.066, 1.745] | 0.039 | – | no | no |
| Main | Dwelling structure | Tukul-sticks | Multi-storey | 469 | 2 | no | 0.722 | [0.336, 1.699] | 0.001 | – | no | no |
| Main | Dwelling structure | Tukul-mud | Incomplete | 897 | 7 | no | −0.526 | [−0.812, −0.191] | 0.004 | – | no | no |
| Main | Dwelling structure | Tukul-mud | House-mud | 897 | 296 | yes | 0.242 | [0.108, 0.343] | 0.001 | 0.001 | no | no |
| Main | Dwelling structure | Tukul-mud | Villa | 897 | 7 | no | 0.548 | [0.015, 1.209] | 0.051 | – | no | no |
| Main | Dwelling structure | Tukul-mud | Flat/apt | 897 | 22 | no | 1.323 | [0.641, 2.248] | 0.001 | – | no | no |
| Main | Dwelling structure | Tukul-mud | House-brick | 897 | 125 | yes | 0.797 | [0.481, 1.071] | 0.001 | 0.001 | no | no |
| Main | Dwelling structure | Tukul-mud | House-wood | 897 | 7 | no | 0.776 | [−0.131, 1.555] | 0.079 | – | no | no |
| Main | Dwelling structure | Tukul-mud | Multi-storey | 897 | 2 | no | 0.558 | [0.183, 1.530] | 0.003 | – | no | no |
| Main | Dwelling structure | Incomplete | House-mud | 7 | 296 | no | 0.768 | [0.384, 1.053] | 0.001 | – | no | no |
| Main | Dwelling structure | Incomplete | Villa | 7 | 7 | no | 1.074 | [0.458, 1.721] | 0.001 | – | no | no |
| Main | Dwelling structure | Incomplete | Flat/apt | 7 | 22 | no | 1.849 | [1.071, 2.792] | 0.001 | – | no | no |
| Main | Dwelling structure | Incomplete | House-brick | 7 | 125 | no | 1.323 | [0.831, 1.678] | 0.001 | – | no | no |
| Main | Dwelling structure | Incomplete | House-wood | 7 | 7 | no | 1.302 | [0.298, 2.047] | 0.019 | – | no | no |
| Main | Dwelling structure | Incomplete | Multi-storey | 7 | 2 | no | 1.084 | [0.551, 2.085] | 0.001 | – | no | no |
| Main | Dwelling structure | House-mud | Villa | 296 | 7 | no | 0.305 | [−0.245, 0.982] | 0.210 | – | no | no |
| Main | Dwelling structure | House-mud | Flat/apt | 296 | 22 | no | 1.081 | [0.407, 2.038] | 0.001 | – | no | no |
| Main | Dwelling structure | House-mud | House-brick | 296 | 125 | yes | 0.555 | [0.254, 0.837] | 0.001 | 0.001 | no | no |
| Main | Dwelling structure | House-mud | House-wood | 296 | 7 | no | 0.534 | [−0.354, 1.349] | 0.144 | – | no | no |
| Main | Dwelling structure | House-mud | Multi-storey | 296 | 2 | no | 0.316 | [−0.059, 1.310] | 0.097 | – | no | no |
| Main | Dwelling structure | Villa | Flat/apt | 7 | 22 | no | 0.775 | [−0.176, 1.921] | 0.113 | – | no | no |
| Main | Dwelling structure | Villa | House-brick | 7 | 125 | no | 0.249 | [−0.503, 0.848] | 0.413 | – | no | no |
| Main | Dwelling structure | Villa | House-wood | 7 | 7 | no | 0.228 | [−0.877, 1.163] | 0.778 | – | no | no |
| Main | Dwelling structure | Villa | Multi-storey | 7 | 2 | no | 0.011 | [−0.738, 1.248] | 0.871 | – | no | no |
| Main | Dwelling structure | Flat/apt | House-brick | 22 | 125 | no | −0.526 | [−1.494, 0.160] | 0.126 | – | no | no |
| Main | Dwelling structure | Flat/apt | House-wood | 22 | 7 | no | −0.547 | [−1.888, 0.541] | 0.247 | – | no | no |
| Main | Dwelling structure | Flat/apt | Multi-storey | 22 | 2 | no | −0.765 | [−1.737, 0.483] | 0.277 | – | no | no |
| Main | Dwelling structure | House-brick | House-wood | 125 | 7 | no | −0.021 | [−0.904, 0.798] | 0.770 | – | no | no |
| Main | Dwelling structure | House-brick | Multi-storey | 125 | 2 | no | −0.239 | [−0.591, 0.750] | 0.632 | – | no | no |
| Main | Dwelling structure | House-wood | Multi-storey | 7 | 2 | no | −0.218 | [−1.040, 1.184] | 0.912 | – | no | no |
| Main | Sanitation | Bush | Portable receptacle | 1041 | 4 | no | −0.081 | [−0.503, 0.358] | 0.645 | – | no | no |
| Main | Sanitation | Bush | Pit latrine | 1041 | 774 | yes | 0.341 | [0.239, 0.424] | 0.001 | 0.001 | no | no |
| Main | Sanitation | Bush | Flush | 1041 | 9 | no | 0.846 | [0.021, 2.354] | 0.042 | – | no | no |
| Main | Sanitation | Portable receptacle | Pit latrine | 4 | 774 | no | 0.422 | [−0.043, 0.850] | 0.079 | – | no | no |
| Main | Sanitation | Portable receptacle | Flush | 4 | 9 | no | 0.928 | [0.041, 2.484] | 0.039 | – | no | no |
| Main | Sanitation | Pit latrine | Flush | 774 | 9 | no | 0.506 | [−0.296, 2.001] | 0.216 | – | no | no |
| Main | Drinking water | Open water | Filtered | 1532 | 19 | no | 0.189 | [−0.891, 0.462] | 0.458 | – | no | no |
| Main | Drinking water | Open water | Purchased | 1532 | 221 | yes | 0.335 | [0.223, 0.433] | 0.001 | 0.001 | no | no |
| Main | Drinking water | Open water | Piped to dwelling | 1532 | 67 | yes | 0.315 | [−0.022, 0.685] | 0.078 | 0.091 | no | no |
| Main | Drinking water | Filtered | Purchased | 19 | 221 | no | 0.146 | [−0.160, 1.192] | 0.331 | – | no | no |
| Main | Drinking water | Filtered | Piped to dwelling | 19 | 67 | no | 0.126 | [−0.336, 1.232] | 0.586 | – | no | no |
| Main | Drinking water | Purchased | Piped to dwelling | 221 | 67 | yes | −0.020 | [−0.374, 0.367] | 0.858 | 0.858 | no | no |
| Main | Lighting | No lighting | Grass/firewood | 362 | 705 | yes | −0.048 | [−0.131, 0.039] | 0.315 | 0.348 | yes | no |
| Main | Lighting | No lighting | Lamp/candle/torch | 362 | 689 | yes | 0.346 | [0.241, 0.447] | 0.001 | 0.001 | no | no |
| Main | Lighting | No lighting | Electricity | 362 | 45 | yes | 1.411 | [1.036, 1.864] | 0.001 | 0.001 | no | no |
| Main | Lighting | No lighting | Solar/biogas | 362 | 37 | yes | 1.643 | [1.140, 2.185] | 0.001 | 0.001 | no | no |
| Main | Lighting | Grass/firewood | Lamp/candle/torch | 705 | 689 | yes | 0.394 | [0.257, 0.516] | 0.001 | 0.001 | no | no |
| Main | Lighting | Grass/firewood | Electricity | 705 | 45 | yes | 1.460 | [1.084, 1.897] | 0.001 | 0.001 | no | no |
| Main | Lighting | Grass/firewood | Solar/biogas | 705 | 37 | yes | 1.691 | [1.200, 2.211] | 0.001 | 0.001 | no | no |
| Main | Lighting | Lamp/candle/torch | Electricity | 689 | 45 | yes | 1.066 | [0.715, 1.532] | 0.001 | 0.001 | no | no |
| Main | Lighting | Lamp/candle/torch | Solar/biogas | 689 | 37 | yes | 1.297 | [0.775, 1.850] | 0.001 | 0.001 | no | no |
| Main | Lighting | Electricity | Solar/biogas | 45 | 37 | yes | 0.232 | [−0.365, 0.720] | 0.428 | 0.449 | no | no |
| Main | Cooking fuel | Grass | Firewood | 17 | 1431 | no | 0.121 | [−0.044, 0.290] | 0.128 | – | no | no |
| Main | Cooking fuel | Grass | Charcoal | 17 | 384 | no | 0.873 | [0.588, 1.107] | 0.001 | – | no | no |
| Main | Cooking fuel | Grass | Biogas | 17 | 1 | no | −0.397 | [−0.632, −0.151] | 0.003 | – | no | no |
| Main | Cooking fuel | Firewood | Charcoal | 1431 | 384 | yes | 0.752 | [0.529, 0.910] | 0.001 | 0.001 | no | no |
| Main | Cooking fuel | Firewood | Biogas | 1431 | 1 | no | −0.518 | [−0.713, −0.332] | 0.002 | – | no | no |
| Main | Cooking fuel | Charcoal | Biogas | 384 | 1 | no | −1.270 | [−1.515, −0.990] | 0.002 | – | no | no |
| Asset-only | Dwelling structure | Tent | Tukul-sticks | 6 | 469 | no | 0.491 | [0.098, 1.224] | 0.007 | – | no | no |
| Asset-only | Dwelling structure | Tent | Tukul-mud | 6 | 897 | no | 0.764 | [0.355, 1.528] | 0.001 | – | no | no |
| Asset-only | Dwelling structure | Tent | Incomplete | 6 | 7 | no | −0.138 | [−0.641, 1.105] | 0.960 | – | no | no |
| Asset-only | Dwelling structure | Tent | House-mud | 6 | 296 | no | 0.885 | [0.468, 1.637] | 0.001 | – | no | no |
| Asset-only | Dwelling structure | Tent | Villa | 6 | 7 | no | 1.409 | [0.521, 2.751] | 0.004 | – | no | no |
| Asset-only | Dwelling structure | Tent | Flat/apt | 6 | 22 | no | 1.465 | [0.878, 2.472] | 0.001 | – | no | no |
| Asset-only | Dwelling structure | Tent | House-brick | 6 | 125 | no | 1.258 | [0.778, 2.044] | 0.001 | – | no | no |
| Asset-only | Dwelling structure | Tent | House-wood | 6 | 7 | no | 1.169 | [0.407, 2.388] | 0.002 | – | no | no |
| Asset-only | Dwelling structure | Tent | Multi-storey | 6 | 2 | no | 1.141 | [0.478, 3.281] | 0.001 | – | no | no |
| Asset-only | Dwelling structure | Tukul-sticks | Tukul-mud | 469 | 897 | yes | 0.273 | [0.125, 0.411] | 0.001 | 0.001 | no | no |
| Asset-only | Dwelling structure | Tukul-sticks | Incomplete | 469 | 7 | no | −0.629 | [−0.981, 0.200] | 0.152 | – | no | no |
| Asset-only | Dwelling structure | Tukul-sticks | House-mud | 469 | 296 | yes | 0.394 | [0.245, 0.520] | 0.001 | 0.001 | no | no |
| Asset-only | Dwelling structure | Tukul-sticks | Villa | 469 | 7 | no | 0.919 | [0.095, 2.039] | 0.031 | – | no | no |
| Asset-only | Dwelling structure | Tukul-sticks | Flat/apt | 469 | 22 | no | 0.975 | [0.502, 1.582] | 0.001 | – | no | no |
| Asset-only | Dwelling structure | Tukul-sticks | House-brick | 469 | 125 | yes | 0.767 | [0.466, 1.005] | 0.001 | 0.001 | no | no |
| Asset-only | Dwelling structure | Tukul-sticks | House-wood | 469 | 7 | no | 0.678 | [−0.002, 1.709] | 0.052 | – | no | no |
| Asset-only | Dwelling structure | Tukul-sticks | Multi-storey | 469 | 2 | no | 0.651 | [0.171, 2.383] | 0.003 | – | no | no |
| Asset-only | Dwelling structure | Tukul-mud | Incomplete | 897 | 7 | no | −0.902 | [−1.184, −0.062] | 0.042 | – | no | no |
| Asset-only | Dwelling structure | Tukul-mud | House-mud | 897 | 296 | yes | 0.121 | [−0.004, 0.219] | 0.054 | 0.067 | no | no |
| Asset-only | Dwelling structure | Tukul-mud | Villa | 897 | 7 | no | 0.645 | [−0.155, 1.760] | 0.099 | – | no | no |
| Asset-only | Dwelling structure | Tukul-mud | Flat/apt | 897 | 22 | no | 0.701 | [0.234, 1.320] | 0.004 | – | no | no |
| Asset-only | Dwelling structure | Tukul-mud | House-brick | 897 | 125 | yes | 0.494 | [0.228, 0.723] | 0.001 | 0.001 | no | no |
| Asset-only | Dwelling structure | Tukul-mud | House-wood | 897 | 7 | no | 0.405 | [−0.196, 1.395] | 0.333 | – | no | no |
| Asset-only | Dwelling structure | Tukul-mud | Multi-storey | 897 | 2 | no | 0.378 | [−0.121, 2.099] | 0.178 | – | no | no |
| Asset-only | Dwelling structure | Incomplete | House-mud | 7 | 296 | no | 1.023 | [0.160, 1.325] | 0.035 | – | no | no |
| Asset-only | Dwelling structure | Incomplete | Villa | 7 | 7 | no | 1.548 | [0.366, 2.540] | 0.016 | – | no | no |
| Asset-only | Dwelling structure | Incomplete | Flat/apt | 7 | 22 | no | 1.604 | [0.631, 2.252] | 0.001 | – | no | no |
| Asset-only | Dwelling structure | Incomplete | House-brick | 7 | 125 | no | 1.396 | [0.473, 1.746] | 0.006 | – | no | no |
| Asset-only | Dwelling structure | Incomplete | House-wood | 7 | 7 | no | 1.307 | [0.169, 2.245] | 0.024 | – | no | no |
| Asset-only | Dwelling structure | Incomplete | Multi-storey | 7 | 2 | no | 1.280 | [0.250, 3.076] | 0.021 | – | no | no |
| Asset-only | Dwelling structure | House-mud | Villa | 296 | 7 | no | 0.524 | [−0.296, 1.633] | 0.172 | – | no | no |
| Asset-only | Dwelling structure | House-mud | Flat/apt | 296 | 22 | no | 0.580 | [0.129, 1.197] | 0.011 | – | no | no |
| Asset-only | Dwelling structure | House-mud | House-brick | 296 | 125 | yes | 0.373 | [0.103, 0.619] | 0.010 | 0.013 | no | no |
| Asset-only | Dwelling structure | House-mud | House-wood | 296 | 7 | no | 0.284 | [−0.342, 1.305] | 0.548 | – | no | no |
| Asset-only | Dwelling structure | House-mud | Multi-storey | 296 | 2 | no | 0.257 | [−0.242, 2.011] | 0.426 | – | no | no |
| Asset-only | Dwelling structure | Villa | Flat/apt | 7 | 22 | no | 0.056 | [−1.185, 1.107] | 0.905 | – | no | no |
| Asset-only | Dwelling structure | Villa | House-brick | 7 | 125 | no | −0.152 | [−1.308, 0.654] | 0.686 | – | no | no |
| Asset-only | Dwelling structure | Villa | House-wood | 7 | 7 | no | −0.240 | [−1.502, 1.013] | 0.668 | – | no | no |
| Asset-only | Dwelling structure | Villa | Multi-storey | 7 | 2 | no | −0.268 | [−1.430, 1.888] | 0.836 | – | no | no |
| Asset-only | Dwelling structure | Flat/apt | House-brick | 22 | 125 | no | −0.208 | [−0.907, 0.281] | 0.421 | – | no | no |
| Asset-only | Dwelling structure | Flat/apt | House-wood | 22 | 7 | no | −0.296 | [−1.265, 0.893] | 0.572 | – | no | no |
| Asset-only | Dwelling structure | Flat/apt | Multi-storey | 22 | 2 | no | −0.324 | [−1.178, 1.639] | 0.656 | – | no | no |
| Asset-only | Dwelling structure | House-brick | House-wood | 125 | 7 | no | −0.089 | [−0.742, 0.934] | 0.822 | – | no | no |
| Asset-only | Dwelling structure | House-brick | Multi-storey | 125 | 2 | no | −0.116 | [−0.668, 1.657] | 0.873 | – | no | no |
| Asset-only | Dwelling structure | House-wood | Multi-storey | 7 | 2 | no | −0.027 | [−1.153, 1.895] | 0.817 | – | no | no |
| Asset-only | Sanitation | Bush | Portable receptacle | 1041 | 4 | no | −0.366 | [−0.661, 0.074] | 0.106 | – | no | no |
| Asset-only | Sanitation | Bush | Pit latrine | 1041 | 774 | yes | 0.248 | [0.164, 0.315] | 0.001 | 0.001 | no | no |
| Asset-only | Sanitation | Bush | Flush | 1041 | 9 | no | 0.519 | [0.037, 1.265] | 0.036 | – | no | no |
| Asset-only | Sanitation | Portable receptacle | Pit latrine | 4 | 774 | no | 0.614 | [0.168, 0.914] | 0.003 | – | no | no |
| Asset-only | Sanitation | Portable receptacle | Flush | 4 | 9 | no | 0.885 | [0.286, 1.718] | 0.004 | – | no | no |
| Asset-only | Sanitation | Pit latrine | Flush | 774 | 9 | no | 0.271 | [−0.202, 1.027] | 0.262 | – | no | no |
| Asset-only | Drinking water | Open water | Filtered | 1532 | 19 | no | 0.414 | [−0.191, 0.792] | 0.236 | – | no | no |
| Asset-only | Drinking water | Open water | Purchased | 1532 | 221 | yes | 0.220 | [0.072, 0.341] | 0.004 | 0.006 | no | no |
| Asset-only | Drinking water | Open water | Piped to dwelling | 1532 | 67 | yes | 0.121 | [−0.160, 0.386] | 0.427 | 0.472 | no | no |
| Asset-only | Drinking water | Filtered | Purchased | 19 | 221 | no | −0.194 | [−0.604, 0.422] | 0.633 | – | no | no |
| Asset-only | Drinking water | Filtered | Piped to dwelling | 19 | 67 | no | −0.293 | [−0.768, 0.414] | 0.459 | – | no | no |
| Asset-only | Drinking water | Purchased | Piped to dwelling | 221 | 67 | yes | −0.099 | [−0.399, 0.214] | 0.520 | 0.546 | no | no |
| Asset-only | Lighting | No lighting | Grass/firewood | 362 | 705 | yes | 0.015 | [−0.073, 0.103] | 0.685 | 0.685 | yes | no |
| Asset-only | Lighting | No lighting | Lamp/candle/torch | 362 | 689 | yes | 0.420 | [0.295, 0.530] | 0.001 | 0.001 | no | no |
| Asset-only | Lighting | No lighting | Electricity | 362 | 45 | yes | 1.295 | [1.005, 1.642] | 0.001 | 0.001 | no | no |
| Asset-only | Lighting | No lighting | Solar/biogas | 362 | 37 | yes | 1.462 | [1.144, 1.815] | 0.001 | 0.001 | no | no |
| Asset-only | Lighting | Grass/firewood | Lamp/candle/torch | 705 | 689 | yes | 0.406 | [0.264, 0.526] | 0.001 | 0.001 | no | no |
| Asset-only | Lighting | Grass/firewood | Electricity | 705 | 45 | yes | 1.280 | [0.993, 1.620] | 0.001 | 0.001 | no | no |
| Asset-only | Lighting | Grass/firewood | Solar/biogas | 705 | 37 | yes | 1.448 | [1.129, 1.776] | 0.001 | 0.001 | no | no |
| Asset-only | Lighting | Lamp/candle/torch | Electricity | 689 | 45 | yes | 0.875 | [0.600, 1.224] | 0.001 | 0.001 | no | no |
| Asset-only | Lighting | Lamp/candle/torch | Solar/biogas | 689 | 37 | yes | 1.042 | [0.715, 1.430] | 0.001 | 0.001 | no | no |
| Asset-only | Lighting | Electricity | Solar/biogas | 45 | 37 | yes | 0.167 | [−0.227, 0.488] | 0.408 | 0.472 | no | no |
| Asset-only | Cooking fuel | Grass | Firewood | 17 | 1431 | no | 0.172 | [−0.150, 0.438] | 0.237 | – | no | no |
| Asset-only | Cooking fuel | Grass | Charcoal | 17 | 384 | no | 0.760 | [0.320, 1.139] | 0.005 | – | no | no |
| Asset-only | Cooking fuel | Grass | Biogas | 17 | 1 | no | −0.424 | [−0.764, −0.129] | 0.008 | – | no | no |
| Asset-only | Cooking fuel | Firewood | Charcoal | 1431 | 384 | yes | 0.588 | [0.295, 0.808] | 0.001 | 0.001 | no | no |
| Asset-only | Cooking fuel | Firewood | Biogas | 1431 | 1 | no | −0.596 | [−0.746, −0.450] | 0.002 | – | no | no |
| Asset-only | Cooking fuel | Charcoal | Biogas | 384 | 1 | no | −1.183 | [−1.428, −0.870] | 0.002 | – | no | no |
| Pre-revision | Dwelling structure | Tent | Tukul-sticks | 6 | 469 | no | 0.392 | [0.145, 0.742] | 0.003 | – | no | no |
| Pre-revision | Dwelling structure | Tent | Tukul-mud | 6 | 897 | no | 0.601 | [0.354, 0.939] | 0.001 | – | no | no |
| Pre-revision | Dwelling structure | Tent | Incomplete | 6 | 7 | no | 0.029 | [−0.311, 0.664] | 0.679 | – | no | no |
| Pre-revision | Dwelling structure | Tent | House-mud | 6 | 296 | no | 0.802 | [0.522, 1.159] | 0.001 | – | no | no |
| Pre-revision | Dwelling structure | Tent | Villa | 6 | 7 | no | 1.193 | [0.474, 1.930] | 0.002 | – | no | no |
| Pre-revision | Dwelling structure | Tent | Flat/apt | 6 | 22 | no | 1.803 | [1.154, 2.658] | 0.001 | – | no | no |
| Pre-revision | Dwelling structure | Tent | House-brick | 6 | 125 | no | 1.337 | [0.978, 1.747] | 0.001 | – | no | no |
| Pre-revision | Dwelling structure | Tent | House-wood | 6 | 7 | no | 1.487 | [0.683, 2.180] | 0.001 | – | no | no |
| Pre-revision | Dwelling structure | Tent | Multi-storey | 6 | 2 | no | 0.807 | [0.328, 2.141] | 0.002 | – | no | no |
| Pre-revision | Dwelling structure | Tukul-sticks | Tukul-mud | 469 | 897 | yes | 0.208 | [0.098, 0.297] | 0.001 | 0.001 | no | no |
| Pre-revision | Dwelling structure | Tukul-sticks | Incomplete | 469 | 7 | no | −0.363 | [−0.628, 0.114] | 0.162 | – | no | no |
| Pre-revision | Dwelling structure | Tukul-sticks | House-mud | 469 | 296 | yes | 0.409 | [0.251, 0.539] | 0.001 | 0.001 | no | no |
| Pre-revision | Dwelling structure | Tukul-sticks | Villa | 469 | 7 | no | 0.801 | [0.164, 1.449] | 0.025 | – | no | no |
| Pre-revision | Dwelling structure | Tukul-sticks | Flat/apt | 469 | 22 | no | 1.410 | [0.733, 2.296] | 0.001 | – | no | no |
| Pre-revision | Dwelling structure | Tukul-sticks | House-brick | 469 | 125 | yes | 0.945 | [0.630, 1.209] | 0.001 | 0.001 | no | no |
| Pre-revision | Dwelling structure | Tukul-sticks | House-wood | 469 | 7 | no | 1.095 | [0.303, 1.769] | 0.004 | – | no | no |
| Pre-revision | Dwelling structure | Tukul-sticks | Multi-storey | 469 | 2 | no | 0.414 | [−0.007, 1.653] | 0.061 | – | no | no |
| Pre-revision | Dwelling structure | Tukul-mud | Incomplete | 897 | 7 | no | −0.571 | [−0.795, −0.109] | 0.015 | – | no | no |
| Pre-revision | Dwelling structure | Tukul-mud | House-mud | 897 | 296 | yes | 0.201 | [0.082, 0.301] | 0.001 | 0.001 | no | no |
| Pre-revision | Dwelling structure | Tukul-mud | Villa | 897 | 7 | no | 0.593 | [−0.045, 1.249] | 0.063 | – | no | no |
| Pre-revision | Dwelling structure | Tukul-mud | Flat/apt | 897 | 22 | no | 1.202 | [0.551, 2.091] | 0.001 | – | no | no |
| Pre-revision | Dwelling structure | Tukul-mud | House-brick | 897 | 125 | yes | 0.737 | [0.434, 1.007] | 0.001 | 0.001 | no | no |
| Pre-revision | Dwelling structure | Tukul-mud | House-wood | 897 | 7 | no | 0.887 | [0.056, 1.546] | 0.028 | – | no | no |
| Pre-revision | Dwelling structure | Tukul-mud | Multi-storey | 897 | 2 | no | 0.206 | [−0.207, 1.444] | 0.380 | – | no | no |
| Pre-revision | Dwelling structure | Incomplete | House-mud | 7 | 296 | no | 0.772 | [0.271, 1.010] | 0.001 | – | no | no |
| Pre-revision | Dwelling structure | Incomplete | Villa | 7 | 7 | no | 1.164 | [0.345, 1.763] | 0.007 | – | no | no |
| Pre-revision | Dwelling structure | Incomplete | Flat/apt | 7 | 22 | no | 1.773 | [0.958, 2.651] | 0.001 | – | no | no |
| Pre-revision | Dwelling structure | Incomplete | House-brick | 7 | 125 | no | 1.308 | [0.742, 1.632] | 0.001 | – | no | no |
| Pre-revision | Dwelling structure | Incomplete | House-wood | 7 | 7 | no | 1.458 | [0.537, 2.065] | 0.003 | – | no | no |
| Pre-revision | Dwelling structure | Incomplete | Multi-storey | 7 | 2 | no | 0.777 | [0.128, 2.057] | 0.023 | – | no | no |
| Pre-revision | Dwelling structure | House-mud | Villa | 296 | 7 | no | 0.391 | [−0.254, 1.060] | 0.169 | – | no | no |
| Pre-revision | Dwelling structure | House-mud | Flat/apt | 296 | 22 | no | 1.001 | [0.376, 1.913] | 0.001 | – | no | no |
| Pre-revision | Dwelling structure | House-mud | House-brick | 296 | 125 | yes | 0.536 | [0.237, 0.819] | 0.002 | 0.002 | no | no |
| Pre-revision | Dwelling structure | House-mud | House-wood | 296 | 7 | no | 0.686 | [−0.122, 1.368] | 0.084 | – | no | no |
| Pre-revision | Dwelling structure | House-mud | Multi-storey | 296 | 2 | no | 0.005 | [−0.427, 1.259] | 0.933 | – | no | no |
| Pre-revision | Dwelling structure | Villa | Flat/apt | 7 | 22 | no | 0.609 | [−0.339, 1.754] | 0.214 | – | no | no |
| Pre-revision | Dwelling structure | Villa | House-brick | 7 | 125 | no | 0.144 | [−0.596, 0.806] | 0.670 | – | no | no |
| Pre-revision | Dwelling structure | Villa | House-wood | 7 | 7 | no | 0.294 | [−0.694, 1.187] | 0.636 | – | no | no |
| Pre-revision | Dwelling structure | Villa | Multi-storey | 7 | 2 | no | −0.387 | [−1.180, 1.214] | 0.657 | – | no | no |
| Pre-revision | Dwelling structure | Flat/apt | House-brick | 22 | 125 | no | −0.465 | [−1.392, 0.204] | 0.172 | – | no | no |
| Pre-revision | Dwelling structure | Flat/apt | House-wood | 22 | 7 | no | −0.315 | [−1.561, 0.685] | 0.424 | – | no | no |
| Pre-revision | Dwelling structure | Flat/apt | Multi-storey | 22 | 2 | no | −0.996 | [−1.989, 0.542] | 0.325 | – | no | no |
| Pre-revision | Dwelling structure | House-brick | House-wood | 125 | 7 | no | 0.150 | [−0.642, 0.830] | 0.783 | – | no | no |
| Pre-revision | Dwelling structure | House-brick | Multi-storey | 125 | 2 | no | −0.531 | [−0.966, 0.745] | 0.532 | – | no | no |
| Pre-revision | Dwelling structure | House-wood | Multi-storey | 7 | 2 | no | −0.681 | [−1.409, 0.864] | 0.516 | – | no | no |
| Pre-revision | Sanitation | Bush | Portable receptacle | 1041 | 4 | no | −0.341 | [−0.843, 0.064] | 0.129 | – | no | no |
| Pre-revision | Sanitation | Bush | Pit latrine | 1041 | 774 | yes | 0.293 | [0.204, 0.361] | 0.001 | 0.001 | no | no |
| Pre-revision | Sanitation | Bush | Flush | 1041 | 9 | no | 0.240 | [−0.449, 1.758] | 0.537 | – | no | no |
| Pre-revision | Sanitation | Portable receptacle | Pit latrine | 4 | 774 | no | 0.634 | [0.209, 1.125] | 0.004 | – | no | no |
| Pre-revision | Sanitation | Portable receptacle | Flush | 4 | 9 | no | 0.581 | [−0.205, 2.267] | 0.172 | – | no | no |
| Pre-revision | Sanitation | Pit latrine | Flush | 774 | 9 | no | −0.053 | [−0.732, 1.506] | 0.934 | – | no | no |
| Pre-revision | Drinking water | Open water | Filtered | 1532 | 19 | no | 0.450 | [−0.808, 0.827] | 0.288 | – | no | no |
| Pre-revision | Drinking water | Open water | Purchased | 1532 | 221 | yes | 0.256 | [0.121, 0.368] | 0.001 | 0.001 | no | no |
| Pre-revision | Drinking water | Open water | Piped to dwelling | 1532 | 67 | yes | 0.186 | [−0.075, 0.464] | 0.195 | 0.227 | no | no |
| Pre-revision | Drinking water | Filtered | Purchased | 19 | 221 | no | −0.194 | [−0.599, 1.051] | 0.592 | – | no | no |
| Pre-revision | Drinking water | Filtered | Piped to dwelling | 19 | 67 | no | −0.264 | [−0.759, 1.001] | 0.533 | – | no | no |
| Pre-revision | Drinking water | Purchased | Piped to dwelling | 221 | 67 | yes | −0.070 | [−0.349, 0.242] | 0.641 | 0.673 | no | no |
| Pre-revision | Lighting | No lighting | Grass/firewood | 362 | 705 | yes | 0.025 | [−0.073, 0.130] | 0.595 | 0.657 | yes | no |
| Pre-revision | Lighting | No lighting | Lamp/candle/torch | 362 | 689 | yes | 0.396 | [0.267, 0.510] | 0.001 | 0.001 | no | no |
| Pre-revision | Lighting | No lighting | Electricity | 362 | 45 | yes | 1.165 | [0.885, 1.469] | 0.001 | 0.001 | no | no |
| Pre-revision | Lighting | No lighting | Solar/biogas | 362 | 37 | yes | 1.249 | [0.931, 1.546] | 0.001 | 0.001 | no | no |
| Pre-revision | Lighting | Grass/firewood | Lamp/candle/torch | 705 | 689 | yes | 0.371 | [0.231, 0.494] | 0.001 | 0.001 | no | no |
| Pre-revision | Lighting | Grass/firewood | Electricity | 705 | 45 | yes | 1.140 | [0.879, 1.424] | 0.001 | 0.001 | no | no |
| Pre-revision | Lighting | Grass/firewood | Solar/biogas | 705 | 37 | yes | 1.224 | [0.909, 1.508] | 0.001 | 0.001 | no | no |
| Pre-revision | Lighting | Lamp/candle/torch | Electricity | 689 | 45 | yes | 0.770 | [0.516, 1.080] | 0.001 | 0.001 | no | no |
| Pre-revision | Lighting | Lamp/candle/torch | Solar/biogas | 689 | 37 | yes | 0.853 | [0.506, 1.194] | 0.001 | 0.001 | no | no |
| Pre-revision | Lighting | Electricity | Solar/biogas | 45 | 37 | yes | 0.083 | [−0.304, 0.387] | 0.743 | 0.743 | no | no |
| Pre-revision | Cooking fuel | Grass | Firewood | 17 | 1431 | no | 0.054 | [−0.123, 0.216] | 0.474 | – | yes | no |
| Pre-revision | Cooking fuel | Grass | Charcoal | 17 | 384 | no | 0.754 | [0.445, 0.985] | 0.001 | – | no | no |
| Pre-revision | Cooking fuel | Grass | Biogas | 17 | 1 | no | −0.674 | [−0.917, −0.420] | 0.002 | – | no | no |
| Pre-revision | Cooking fuel | Firewood | Charcoal | 1431 | 384 | yes | 0.700 | [0.463, 0.858] | 0.001 | 0.001 | no | no |
| Pre-revision | Cooking fuel | Firewood | Biogas | 1431 | 1 | no | −0.729 | [−0.907, −0.553] | 0.002 | – | no | no |
| Pre-revision | Cooking fuel | Charcoal | Biogas | 384 | 1 | no | −1.429 | [−1.654, −1.154] | 0.002 | – | no | no |
| Consumption | Dwelling structure | Tent | Tukul-sticks | 6 | 472 | no | 0.037 | [−0.439, 0.635] | 0.969 | – | no | no |
| Consumption | Dwelling structure | Tent | Tukul-mud | 6 | 901 | no | 0.095 | [−0.364, 0.728] | 0.727 | – | no | no |
| Consumption | Dwelling structure | Tent | Incomplete | 6 | 7 | no | 0.107 | [−0.880, 2.039] | 0.821 | – | no | no |
| Consumption | Dwelling structure | Tent | House-mud | 6 | 296 | no | 0.201 | [−0.278, 0.809] | 0.400 | – | no | no |
| Consumption | Dwelling structure | Tent | Villa | 6 | 7 | no | 0.032 | [−0.826, 0.882] | 0.997 | – | no | no |
| Consumption | Dwelling structure | Tent | Flat/apt | 6 | 22 | no | 0.321 | [−0.115, 0.922] | 0.119 | – | no | no |
| Consumption | Dwelling structure | Tent | House-brick | 6 | 125 | no | 0.545 | [0.038, 1.158] | 0.039 | – | no | no |
| Consumption | Dwelling structure | Tent | House-wood | 6 | 7 | no | 0.607 | [−0.480, 1.272] | 0.178 | – | no | no |
| Consumption | Dwelling structure | Tent | Multi-storey | 6 | 2 | no | 0.592 | [−0.018, 1.721] | 0.054 | – | no | no |
| Consumption | Dwelling structure | Tukul-sticks | Tukul-mud | 472 | 901 | yes | 0.058 | [−0.062, 0.202] | 0.340 | 0.357 | yes | no |
| Consumption | Dwelling structure | Tukul-sticks | Incomplete | 472 | 7 | no | 0.070 | [−0.755, 1.931] | 0.798 | – | no | no |
| Consumption | Dwelling structure | Tukul-sticks | House-mud | 472 | 296 | yes | 0.164 | [0.014, 0.319] | 0.032 | 0.040 | no | no |
| Consumption | Dwelling structure | Tukul-sticks | Villa | 472 | 7 | no | −0.005 | [−0.773, 0.676] | 0.971 | – | no | no |
| Consumption | Dwelling structure | Tukul-sticks | Flat/apt | 472 | 22 | no | 0.284 | [0.031, 0.625] | 0.031 | – | no | no |
| Consumption | Dwelling structure | Tukul-sticks | House-brick | 472 | 125 | yes | 0.508 | [0.330, 0.684] | 0.001 | 0.002 | no | no |
| Consumption | Dwelling structure | Tukul-sticks | House-wood | 472 | 7 | no | 0.570 | [−0.430, 0.944] | 0.132 | – | no | no |
| Consumption | Dwelling structure | Tukul-sticks | Multi-storey | 472 | 2 | no | 0.555 | [0.216, 1.433] | 0.001 | – | no | no |
| Consumption | Dwelling structure | Tukul-mud | Incomplete | 901 | 7 | no | 0.012 | [−0.798, 1.841] | 0.910 | – | no | no |
| Consumption | Dwelling structure | Tukul-mud | House-mud | 901 | 296 | yes | 0.106 | [−0.015, 0.208] | 0.091 | 0.106 | yes | no |
| Consumption | Dwelling structure | Tukul-mud | Villa | 901 | 7 | no | −0.063 | [−0.821, 0.586] | 0.849 | – | no | no |
| Consumption | Dwelling structure | Tukul-mud | Flat/apt | 901 | 22 | no | 0.226 | [−0.023, 0.571] | 0.076 | – | no | no |
| Consumption | Dwelling structure | Tukul-mud | House-brick | 901 | 125 | yes | 0.449 | [0.281, 0.609] | 0.001 | 0.002 | no | no |
| Consumption | Dwelling structure | Tukul-mud | House-wood | 901 | 7 | no | 0.511 | [−0.547, 0.881] | 0.174 | – | no | no |
| Consumption | Dwelling structure | Tukul-mud | Multi-storey | 901 | 2 | no | 0.497 | [0.157, 1.375] | 0.001 | – | no | no |
| Consumption | Dwelling structure | Incomplete | House-mud | 7 | 296 | no | 0.094 | [−1.770, 0.906] | 0.877 | – | no | no |
| Consumption | Dwelling structure | Incomplete | Villa | 7 | 7 | no | −0.075 | [−2.118, 1.021] | 0.811 | – | no | no |
| Consumption | Dwelling structure | Incomplete | Flat/apt | 7 | 22 | no | 0.214 | [−1.625, 1.130] | 0.756 | – | no | no |
| Consumption | Dwelling structure | Incomplete | House-brick | 7 | 125 | no | 0.437 | [−1.402, 1.272] | 0.587 | – | no | no |
| Consumption | Dwelling structure | Incomplete | House-wood | 7 | 7 | no | 0.499 | [−1.516, 1.425] | 0.692 | – | no | no |
| Consumption | Dwelling structure | Incomplete | Multi-storey | 7 | 2 | no | 0.485 | [−1.295, 1.897] | 0.538 | – | no | no |
| Consumption | Dwelling structure | House-mud | Villa | 296 | 7 | no | −0.169 | [−0.944, 0.528] | 0.652 | – | no | no |
| Consumption | Dwelling structure | House-mud | Flat/apt | 296 | 22 | no | 0.120 | [−0.138, 0.475] | 0.386 | – | no | no |
| Consumption | Dwelling structure | House-mud | House-brick | 296 | 125 | yes | 0.344 | [0.157, 0.532] | 0.003 | 0.004 | no | no |
| Consumption | Dwelling structure | House-mud | House-wood | 296 | 7 | no | 0.406 | [−0.646, 0.771] | 0.243 | – | no | no |
| Consumption | Dwelling structure | House-mud | Multi-storey | 296 | 2 | no | 0.391 | [0.043, 1.258] | 0.031 | – | no | no |
| Consumption | Dwelling structure | Villa | Flat/apt | 7 | 22 | no | 0.289 | [−0.428, 1.145] | 0.437 | – | no | no |
| Consumption | Dwelling structure | Villa | House-brick | 7 | 125 | no | 0.512 | [−0.173, 1.295] | 0.182 | – | no | no |
| Consumption | Dwelling structure | Villa | House-wood | 7 | 7 | no | 0.574 | [−0.582, 1.407] | 0.259 | – | no | no |
| Consumption | Dwelling structure | Villa | Multi-storey | 7 | 2 | no | 0.560 | [−0.199, 1.887] | 0.154 | – | no | no |
| Consumption | Dwelling structure | Flat/apt | House-brick | 22 | 125 | no | 0.224 | [−0.149, 0.503] | 0.220 | – | no | no |
| Consumption | Dwelling structure | Flat/apt | House-wood | 22 | 7 | no | 0.286 | [−0.814, 0.679] | 0.412 | – | no | no |
| Consumption | Dwelling structure | Flat/apt | Multi-storey | 22 | 2 | no | 0.271 | [−0.150, 1.198] | 0.215 | – | no | no |
| Consumption | Dwelling structure | House-brick | House-wood | 125 | 7 | no | 0.062 | [−0.975, 0.502] | 0.875 | – | no | no |
| Consumption | Dwelling structure | House-brick | Multi-storey | 125 | 2 | no | 0.048 | [−0.326, 0.938] | 0.722 | – | no | no |
| Consumption | Dwelling structure | House-wood | Multi-storey | 7 | 2 | no | −0.014 | [−0.540, 1.443] | 0.851 | – | no | no |
| Consumption | Sanitation | Bush | Portable receptacle | 1047 | 4 | no | 0.827 | [−0.277, 1.874] | 0.151 | – | no | no |
| Consumption | Sanitation | Bush | Pit latrine | 1047 | 775 | yes | 0.163 | [0.095, 0.226] | 0.001 | 0.002 | no | no |
| Consumption | Sanitation | Bush | Flush | 1047 | 9 | no | 0.742 | [0.249, 1.238] | 0.003 | – | no | no |
| Consumption | Sanitation | Portable receptacle | Pit latrine | 4 | 775 | no | −0.664 | [−1.708, 0.434] | 0.213 | – | no | no |
| Consumption | Sanitation | Portable receptacle | Flush | 4 | 9 | no | −0.085 | [−1.254, 1.114] | 0.830 | – | no | no |
| Consumption | Sanitation | Pit latrine | Flush | 775 | 9 | no | 0.578 | [0.095, 1.081] | 0.021 | – | no | no |
| Consumption | Drinking water | Open water | Filtered | 1539 | 19 | no | −0.069 | [−0.992, 0.278] | 0.645 | – | no | no |
| Consumption | Drinking water | Open water | Purchased | 1539 | 221 | yes | 0.285 | [0.159, 0.418] | 0.001 | 0.002 | no | no |
| Consumption | Drinking water | Open water | Piped to dwelling | 1539 | 67 | yes | 0.510 | [0.272, 0.745] | 0.001 | 0.002 | no | no |
| Consumption | Drinking water | Filtered | Purchased | 19 | 221 | no | 0.354 | [−0.033, 1.268] | 0.070 | – | no | no |
| Consumption | Drinking water | Filtered | Piped to dwelling | 19 | 67 | no | 0.579 | [0.136, 1.575] | 0.020 | – | no | no |
| Consumption | Drinking water | Purchased | Piped to dwelling | 221 | 67 | yes | 0.224 | [−0.055, 0.488] | 0.105 | 0.116 | no | no |
| Consumption | Lighting | No lighting | Grass/firewood | 364 | 708 | yes | 0.227 | [0.094, 0.376] | 0.003 | 0.004 | no | no |
| Consumption | Lighting | No lighting | Lamp/candle/torch | 364 | 691 | yes | 0.605 | [0.432, 0.768] | 0.001 | 0.002 | no | no |
| Consumption | Lighting | No lighting | Electricity | 364 | 45 | yes | 0.992 | [0.692, 1.312] | 0.001 | 0.002 | no | no |
| Consumption | Lighting | No lighting | Solar/biogas | 364 | 37 | yes | 0.878 | [0.636, 1.101] | 0.001 | 0.002 | no | no |
| Consumption | Lighting | Grass/firewood | Lamp/candle/torch | 708 | 691 | yes | 0.378 | [0.243, 0.483] | 0.001 | 0.002 | no | no |
| Consumption | Lighting | Grass/firewood | Electricity | 708 | 45 | yes | 0.765 | [0.463, 1.092] | 0.001 | 0.002 | no | no |
| Consumption | Lighting | Grass/firewood | Solar/biogas | 708 | 37 | yes | 0.651 | [0.418, 0.849] | 0.001 | 0.002 | no | no |
| Consumption | Lighting | Lamp/candle/torch | Electricity | 691 | 45 | yes | 0.387 | [0.093, 0.731] | 0.005 | 0.007 | no | no |
| Consumption | Lighting | Lamp/candle/torch | Solar/biogas | 691 | 37 | yes | 0.273 | [0.047, 0.501] | 0.017 | 0.022 | no | no |
| Consumption | Lighting | Electricity | Solar/biogas | 45 | 37 | yes | −0.114 | [−0.455, 0.208] | 0.454 | 0.454 | no | no |
| Consumption | Cooking fuel | Grass | Firewood | 17 | 1437 | no | 0.672 | [−0.260, 1.217] | 0.130 | – | no | no |
| Consumption | Cooking fuel | Grass | Charcoal | 17 | 385 | no | 1.051 | [0.167, 1.600] | 0.023 | – | no | no |
| Consumption | Cooking fuel | Grass | Biogas | 17 | 1 | no | 1.380 | [0.483, 1.920] | 0.005 | – | no | no |
| Consumption | Cooking fuel | Firewood | Charcoal | 1437 | 385 | yes | 0.379 | [0.188, 0.532] | 0.001 | 0.002 | no | no |
| Consumption | Cooking fuel | Firewood | Biogas | 1437 | 1 | no | 0.709 | [0.535, 0.875] | 0.002 | – | no | no |
| Consumption | Cooking fuel | Charcoal | Biogas | 385 | 1 | no | 0.329 | [0.147, 0.570] | 0.002 | – | no | no |
