# Supplementary material for: Wealth-anchored category validation: A diagnostic for categorical orderings in fragile-state household surveys
Source: PLoS One. 2026 Oct 29;21(10):e0360752. doi: 10.1371/journal.pone.0360752 (PMC13718975; doi:10.1371/journal.pone.0360752)
Supplement: Supplementary file 3 [file pone.0360752.s003.docx]

**S3 Table. All pairwise category comparisons in Waves 1 and 2, main anchor.**

Difference is the weighted mean of the location-adjusted anchor in the category the coding places higher minus that in the category it places lower, in anchor standard-deviation units, taken directly from the bootstrap distribution of the difference (2,000 stratified cluster-bootstrap replications, the whole estimation chain re-estimated in each). A negative difference with an adjusted p below 0.05 is a robust inversion. Adjusted p is Benjamini–Hochberg across the whole family of pairs within an anchor; it is blank for pairs below the precision floor, which are reported but never classified. Equivalent means both one-sided tests against a tolerance of 0.20 standard deviations reject. Wave 1 does not contain the dwelling-structure question. Wave 2's public file carries neither a stratum variable nor an urban indicator, so its bootstrap is stratified by state and its location adjustment uses state alone.

| **Wave** | **Anchor** | **Variable** | **Lower category (assigned)** | **Higher category (assigned)** | **n lower** | **n higher** | **Both above floor** | **Difference (SD)** | **95% CI** | **p** | **Adjusted p** | **Equivalent** | **Robust inversion** |
| --- | --- | --- | --- | --- | --- | --- | --- | --- | --- | --- | --- | --- | --- |
| 1 | Main | Sanitation | Bush | Portable receptacle | 1648 | 7 | no | 0.499 | [−0.244, 0.941] | 0.261 | – | no | no |
| 1 | Main | Sanitation | Bush | Pit latrine | 1648 | 1598 | yes | 0.404 | [0.262, 0.518] | 0.001 | 0.002 | no | no |
| 1 | Main | Sanitation | Bush | Flush | 1648 | 25 | no | 0.477 | [0.073, 1.005] | 0.022 | – | no | no |
| 1 | Main | Sanitation | Portable receptacle | Pit latrine | 7 | 1598 | no | −0.094 | [−0.521, 0.602] | 0.932 | – | no | no |
| 1 | Main | Sanitation | Portable receptacle | Flush | 7 | 25 | no | −0.021 | [−0.633, 0.913] | 0.810 | – | no | no |
| 1 | Main | Sanitation | Pit latrine | Flush | 1598 | 25 | no | 0.073 | [−0.312, 0.634] | 0.677 | – | no | no |
| 1 | Main | Drinking water | Open water | Filtered | 2846 | 77 | yes | 0.013 | [−0.218, 0.232] | 0.972 | 0.972 | yes | no |
| 1 | Main | Drinking water | Open water | Purchased | 2846 | 265 | yes | 0.471 | [−0.139, 0.762] | 0.220 | 0.288 | no | no |
| 1 | Main | Drinking water | Filtered | Purchased | 77 | 265 | yes | 0.458 | [−0.145, 0.750] | 0.172 | 0.244 | no | no |
| 1 | Main | Lighting | No lighting | Grass/firewood | 262 | 1020 | yes | 0.073 | [−0.065, 0.219] | 0.271 | 0.329 | yes | no |
| 1 | Main | Lighting | No lighting | Lamp/candle/torch | 262 | 1794 | yes | 0.311 | [0.176, 0.450] | 0.001 | 0.002 | no | no |
| 1 | Main | Lighting | No lighting | Electricity | 262 | 100 | yes | 1.265 | [0.872, 1.500] | 0.001 | 0.002 | no | no |
| 1 | Main | Lighting | No lighting | Solar/biogas | 262 | 105 | yes | 1.046 | [0.597, 1.469] | 0.001 | 0.002 | no | no |
| 1 | Main | Lighting | Grass/firewood | Lamp/candle/torch | 1020 | 1794 | yes | 0.238 | [0.120, 0.324] | 0.001 | 0.002 | no | no |
| 1 | Main | Lighting | Grass/firewood | Electricity | 1020 | 100 | yes | 1.192 | [0.820, 1.440] | 0.001 | 0.002 | no | no |
| 1 | Main | Lighting | Grass/firewood | Solar/biogas | 1020 | 105 | yes | 0.973 | [0.577, 1.343] | 0.001 | 0.002 | no | no |
| 1 | Main | Lighting | Lamp/candle/torch | Electricity | 1794 | 100 | yes | 0.954 | [0.644, 1.174] | 0.001 | 0.002 | no | no |
| 1 | Main | Lighting | Lamp/candle/torch | Solar/biogas | 1794 | 105 | yes | 0.735 | [0.381, 1.081] | 0.001 | 0.002 | no | no |
| 1 | Main | Lighting | Electricity | Solar/biogas | 100 | 105 | yes | −0.219 | [−0.601, 0.252] | 0.457 | 0.518 | no | no |
| 1 | Main | Cooking fuel | Grass | Firewood | 45 | 2280 | yes | −0.078 | [−0.270, 0.164] | 0.564 | 0.599 | no | no |
| 1 | Main | Cooking fuel | Grass | Charcoal | 45 | 947 | yes | 0.436 | [0.194, 0.660] | 0.001 | 0.002 | no | no |
| 1 | Main | Cooking fuel | Firewood | Charcoal | 2280 | 947 | yes | 0.514 | [0.314, 0.678] | 0.001 | 0.002 | no | no |
| 2 | Main | Dwelling structure | Tent | Tukul-sticks | 9 | 232 | no | 0.132 | [−0.770, 0.697] | 0.759 | – | no | no |
| 2 | Main | Dwelling structure | Tent | Tukul-mud | 9 | 538 | no | 0.249 | [−0.656, 0.785] | 0.599 | – | no | no |
| 2 | Main | Dwelling structure | Tent | Incomplete | 9 | 8 | no | 0.165 | [−0.828, 0.770] | 0.872 | – | no | no |
| 2 | Main | Dwelling structure | Tent | House-mud | 9 | 210 | no | 0.390 | [−0.493, 0.916] | 0.383 | – | no | no |
| 2 | Main | Dwelling structure | Tent | Villa | 9 | 2 | no | 0.599 | [−0.477, 1.575] | 0.251 | – | no | no |
| 2 | Main | Dwelling structure | Tent | Flat/apt | 9 | 31 | no | 1.544 | [0.331, 2.472] | 0.007 | – | no | no |
| 2 | Main | Dwelling structure | Tent | House-brick | 9 | 138 | no | 0.764 | [−0.161, 1.350] | 0.120 | – | no | no |
| 2 | Main | Dwelling structure | Tent | House-wood | 9 | 9 | no | 0.394 | [−0.753, 1.100] | 0.569 | – | no | no |
| 2 | Main | Dwelling structure | Tukul-sticks | Tukul-mud | 232 | 538 | yes | 0.117 | [−0.074, 0.280] | 0.270 | 0.295 | no | no |
| 2 | Main | Dwelling structure | Tukul-sticks | Incomplete | 232 | 8 | no | 0.033 | [−0.596, 0.323] | 0.946 | – | no | no |
| 2 | Main | Dwelling structure | Tukul-sticks | House-mud | 232 | 210 | yes | 0.258 | [0.050, 0.430] | 0.010 | 0.014 | no | no |
| 2 | Main | Dwelling structure | Tukul-sticks | Villa | 232 | 2 | no | 0.467 | [−0.027, 1.217] | 0.082 | – | no | no |
| 2 | Main | Dwelling structure | Tukul-sticks | Flat/apt | 232 | 31 | yes | 1.412 | [0.677, 2.200] | 0.001 | 0.002 | no | no |
| 2 | Main | Dwelling structure | Tukul-sticks | House-brick | 232 | 138 | yes | 0.631 | [0.350, 0.903] | 0.001 | 0.002 | no | no |
| 2 | Main | Dwelling structure | Tukul-sticks | House-wood | 232 | 9 | no | 0.261 | [−0.416, 0.613] | 0.357 | – | no | no |
| 2 | Main | Dwelling structure | Tukul-mud | Incomplete | 538 | 8 | no | −0.084 | [−0.687, 0.202] | 0.546 | – | no | no |
| 2 | Main | Dwelling structure | Tukul-mud | House-mud | 538 | 210 | yes | 0.141 | [0.010, 0.272] | 0.039 | 0.050 | no | no |
| 2 | Main | Dwelling structure | Tukul-mud | Villa | 538 | 2 | no | 0.350 | [−0.103, 1.092] | 0.204 | – | no | no |
| 2 | Main | Dwelling structure | Tukul-mud | Flat/apt | 538 | 31 | yes | 1.295 | [0.584, 2.130] | 0.001 | 0.002 | no | no |
| 2 | Main | Dwelling structure | Tukul-mud | House-brick | 538 | 138 | yes | 0.514 | [0.285, 0.737] | 0.001 | 0.002 | no | no |
| 2 | Main | Dwelling structure | Tukul-mud | House-wood | 538 | 9 | no | 0.144 | [−0.496, 0.476] | 0.579 | – | no | no |
| 2 | Main | Dwelling structure | Incomplete | House-mud | 8 | 210 | no | 0.225 | [−0.094, 0.801] | 0.160 | – | no | no |
| 2 | Main | Dwelling structure | Incomplete | Villa | 8 | 2 | no | 0.434 | [−0.118, 1.407] | 0.151 | – | no | no |
| 2 | Main | Dwelling structure | Incomplete | Flat/apt | 8 | 31 | no | 1.379 | [0.649, 2.350] | 0.001 | – | no | no |
| 2 | Main | Dwelling structure | Incomplete | House-brick | 8 | 138 | no | 0.598 | [0.269, 1.208] | 0.003 | – | no | no |
| 2 | Main | Dwelling structure | Incomplete | House-wood | 8 | 9 | no | 0.228 | [−0.436, 0.826] | 0.365 | – | no | no |
| 2 | Main | Dwelling structure | House-mud | Villa | 210 | 2 | no | 0.209 | [−0.270, 0.965] | 0.543 | – | no | no |
| 2 | Main | Dwelling structure | House-mud | Flat/apt | 210 | 31 | yes | 1.154 | [0.450, 1.973] | 0.001 | 0.002 | no | no |
| 2 | Main | Dwelling structure | House-mud | House-brick | 210 | 138 | yes | 0.374 | [0.097, 0.646] | 0.012 | 0.016 | no | no |
| 2 | Main | Dwelling structure | House-mud | House-wood | 210 | 9 | no | 0.004 | [−0.631, 0.353] | 0.965 | – | no | no |
| 2 | Main | Dwelling structure | Villa | Flat/apt | 2 | 31 | no | 0.945 | [−0.272, 1.905] | 0.136 | – | no | no |
| 2 | Main | Dwelling structure | Villa | House-brick | 2 | 138 | no | 0.165 | [−0.643, 0.683] | 0.717 | – | no | no |
| 2 | Main | Dwelling structure | Villa | House-wood | 2 | 9 | no | −0.205 | [−1.241, 0.395] | 0.516 | – | no | no |
| 2 | Main | Dwelling structure | Flat/apt | House-brick | 31 | 138 | yes | −0.780 | [−1.751, 0.068] | 0.083 | 0.100 | no | no |
| 2 | Main | Dwelling structure | Flat/apt | House-wood | 31 | 9 | no | −1.150 | [−2.111, −0.378] | 0.003 | – | no | no |
| 2 | Main | Dwelling structure | House-brick | House-wood | 138 | 9 | no | −0.370 | [−1.067, 0.019] | 0.072 | – | no | no |
| 2 | Main | Sanitation | Bush | Portable receptacle | 295 | 1 | no | 1.143 | [0.903, 1.310] | 0.002 | – | no | no |
| 2 | Main | Sanitation | Bush | Pit latrine | 295 | 856 | yes | 0.621 | [0.416, 0.740] | 0.001 | 0.002 | no | no |
| 2 | Main | Sanitation | Bush | Flush | 295 | 3 | no | 1.375 | [0.255, 3.938] | 0.001 | – | no | no |
| 2 | Main | Sanitation | Portable receptacle | Pit latrine | 1 | 856 | no | −0.522 | [−0.740, −0.289] | 0.002 | – | no | no |
| 2 | Main | Sanitation | Portable receptacle | Flush | 1 | 3 | no | 0.232 | [−0.892, 2.895] | 0.851 | – | no | no |
| 2 | Main | Sanitation | Pit latrine | Flush | 856 | 3 | no | 0.754 | [−0.301, 3.358] | 0.567 | – | no | no |
| 2 | Main | Drinking water | Open water | Filtered | 1004 | 9 | no | 0.216 | [−0.402, 1.182] | 0.546 | – | no | no |
| 2 | Main | Drinking water | Open water | Purchased | 1004 | 86 | yes | 0.188 | [−0.212, 0.579] | 0.402 | 0.402 | no | no |
| 2 | Main | Drinking water | Filtered | Purchased | 9 | 86 | no | −0.028 | [−1.068, 0.684] | 0.865 | – | no | no |
| 2 | Main | Lighting | No lighting | Grass/firewood | 122 | 86 | yes | −0.588 | [−0.825, −0.224] | 0.001 | 0.002 | no | yes |
| 2 | Main | Lighting | No lighting | Lamp/candle/torch | 122 | 868 | yes | 0.165 | [−0.034, 0.404] | 0.111 | 0.128 | no | no |
| 2 | Main | Lighting | No lighting | Electricity | 122 | 44 | yes | 1.309 | [0.866, 1.751] | 0.001 | 0.002 | no | no |
| 2 | Main | Lighting | No lighting | Solar/biogas | 122 | 55 | yes | 0.979 | [0.654, 1.320] | 0.001 | 0.002 | no | no |
| 2 | Main | Lighting | Grass/firewood | Lamp/candle/torch | 86 | 868 | yes | 0.753 | [0.419, 1.002] | 0.001 | 0.002 | no | no |
| 2 | Main | Lighting | Grass/firewood | Electricity | 86 | 44 | yes | 1.897 | [1.197, 2.436] | 0.001 | 0.002 | no | no |
| 2 | Main | Lighting | Grass/firewood | Solar/biogas | 86 | 55 | yes | 1.567 | [1.102, 1.914] | 0.001 | 0.002 | no | no |
| 2 | Main | Lighting | Lamp/candle/torch | Electricity | 868 | 44 | yes | 1.144 | [0.579, 1.741] | 0.001 | 0.002 | no | no |
| 2 | Main | Lighting | Lamp/candle/torch | Solar/biogas | 868 | 55 | yes | 0.814 | [0.518, 1.108] | 0.001 | 0.002 | no | no |
| 2 | Main | Lighting | Electricity | Solar/biogas | 44 | 55 | yes | −0.330 | [−0.876, 0.220] | 0.282 | 0.295 | no | no |
| 2 | Main | Cooking fuel | Grass | Firewood | 3 | 493 | no | 0.130 | [−0.392, 0.425] | 0.432 | – | no | no |
| 2 | Main | Cooking fuel | Grass | Charcoal | 3 | 665 | no | 0.827 | [0.263, 1.102] | 0.001 | – | no | no |
| 2 | Main | Cooking fuel | Grass | Electricity | 3 | 2 | no | 3.344 | [1.397, 4.020] | 0.001 | – | no | no |
| 2 | Main | Cooking fuel | Grass | Biogas | 3 | 1 | no | 0.239 | [0.021, 0.369] | 0.031 | – | no | no |
| 2 | Main | Cooking fuel | Firewood | Charcoal | 493 | 665 | yes | 0.697 | [0.515, 0.801] | 0.001 | 0.002 | no | no |
| 2 | Main | Cooking fuel | Firewood | Electricity | 493 | 2 | no | 3.214 | [1.346, 3.853] | 0.001 | – | no | no |
| 2 | Main | Cooking fuel | Firewood | Biogas | 493 | 1 | no | 0.109 | [−0.116, 0.257] | 0.382 | – | no | no |
| 2 | Main | Cooking fuel | Charcoal | Electricity | 665 | 2 | no | 2.517 | [0.728, 3.112] | 0.001 | – | no | no |
| 2 | Main | Cooking fuel | Charcoal | Biogas | 665 | 1 | no | −0.588 | [−0.791, −0.374] | 0.002 | – | no | no |
| 2 | Main | Cooking fuel | Electricity | Biogas | 2 | 1 | no | −3.105 | [−3.741, −1.291] | 0.002 | – | no | no |
