# Supplementary material for: Wealth-anchored category validation: A diagnostic for categorical orderings in fragile-state household surveys
Source: PLoS One. 2026 Oct 29;21(10):e0360752. doi: 10.1371/journal.pone.0360752 (PMC13718975; doi:10.1371/journal.pone.0360752)
Supplement: Supplementary file 4 [file pone.0360752.s004.docx]

**S4 Table. Principal-components anchor, Wave 3: eigenvalues, variance explained and first-component loadings, by anchor pool.**

Each column is one survey-weighted principal-components extraction on the analysis sample the corresponding test uses (Section 3.1). The full pool is shown for reference only; every ordering and cut-point test removes the indicators derived from the variable under test, so the anchors actually used are the five leave-one-out (LOO) pools. Loadings are the elements of the first eigenvector of the weighted correlation matrix, oriented so that the component correlates positively with the durable-asset count; a dot marks an indicator that is not in that pool. In the pre-revision pool the original housing binary (dwelling types 1 to 3, tents and stick tukuls) is the only indicator that loads negatively, and the conventional clean-fuel binary, which marks one household, loads at −0.02. Room density is the survey's categorised count of household members per room. Produced by pca_report.py in the deposited code (version 1.0.2).

| **Statistic** | **Full pool** | **LOO: dwelling** | **LOO: sanitation** | **LOO: water** | **LOO: lighting** | **LOO: cooking** | **Asset-only** | **Pre-revision** |
| --- | --- | --- | --- | --- | --- | --- | --- | --- |
| Households in analysis sample | 1839 | 1838 | 1828 | 1839 | 1838 | 1839 | 1839 | 1839 |
| Indicators in pool | 8 | 7 | 7 | 7 | 7 | 7 | 3 | 8 |
| Eigenvalue, first component | 2.72 | 2.48 | 2.29 | 2.63 | 2.50 | 2.32 | 1.44 | 2.21 |
| Eigenvalue, second component | 1.21 | 1.19 | 1.14 | 1.08 | 1.16 | 1.16 | 0.87 | 1.19 |
| Variance explained, first component (%) | 34.0 | 35.4 | 32.7 | 37.6 | 35.7 | 33.1 | 48.1 | 27.6 |
| Variance explained, second component (%) | 15.2 | 17.0 | 16.3 | 15.4 | 16.6 | 16.5 | 28.9 | 14.9 |
| Loading: Improved housing (revised coding) | 0.35 | · | 0.40 | 0.36 | 0.36 | 0.38 | · | · |
| Loading: Improved housing (original analytical coding) | · | · | · | · | · | · | · | −0.28 |
| Loading: Improved drinking water | 0.22 | 0.23 | 0.28 | · | 0.22 | 0.19 | · | 0.15 |
| Loading: Improved sanitation | 0.45 | 0.48 | · | 0.47 | 0.50 | 0.50 | · | 0.52 |
| Loading: Electricity for lighting | 0.34 | 0.35 | 0.42 | 0.34 | · | 0.36 | · | 0.33 |
| Loading: Cooking-fuel cut at charcoal | 0.44 | 0.45 | 0.49 | 0.43 | 0.45 | · | · | · |
| Loading: Clean cooking fuel (conventional definition) | · | · | · | · | · | · | · | −0.02 |
| Loading: Durable asset types owned (count) | 0.42 | 0.46 | 0.46 | 0.43 | 0.44 | 0.47 | 0.64 | 0.49 |
| Loading: Room density | 0.28 | 0.31 | 0.28 | 0.30 | 0.30 | 0.35 | 0.57 | 0.39 |
| Loading: Land ownership | 0.25 | 0.29 | 0.22 | 0.27 | 0.29 | 0.32 | 0.52 | 0.36 |
