# Supplementary material for: Wealth-anchored category validation: A diagnostic for categorical orderings in fragile-state household surveys
Source: PLoS One. 2026 Oct 29;21(10):e0360752. doi: 10.1371/journal.pone.0360752 (PMC13718975; doi:10.1371/journal.pone.0360752)
Supplement: Supplementary file 5 [file pone.0360752.s006.docx]

**REVIEWER PLOS ONE**

| **Manuscript Number** | PONE-D-26-32266 |
| --- | --- |
| **Title** | Wealth-Anchored Category Validation: Detecting Coding Errors in Fragile-State Household Surveys |
| **Recommendation** | Minor Revision |

**Comments to the Author**

The manuscript addresses an important methodological issue in household survey research and proposes a potentially useful procedure, Wealth-Anchored Category Validation (WACV), for identifying category-coding errors. The application to the 2016 South Sudan High Frequency Survey is relevant and clearly demonstrates the practical implications of miscoding, particularly for housing type and cooking fuel. The leave-one-out wealth anchor, location adjustment, cluster-bootstrap inference, and robustness exercises strengthen the methodological contribution. The manuscript is generally well structured and suitable for consideration in PLOS ONE. However, several minor revisions are recommended to improve methodological transparency, interpretation, reproducibility, and presentation.

**Specific Minor Revisions Required**

**1. Clarify the novelty of WACV.** The Introduction and literature review should state more explicitly how WACV differs from existing asset-index validation, sensitivity analysis, and category-scoring approaches. A concise statement explaining the specific methodological advancement would strengthen the contribution.

**2. Provide stronger justification for the wealth-based validation criterion.** The authors should clarify that WACV validates categories against relative household wealth rather than independently establishing whether a service is technically, environmentally, or health-wise “improved.” This distinction is particularly important for cooking fuel.

**3. Clarify the terminology surrounding cooking fuel.** Table 3 labels the WACV classification as an “Improved” cooking share although charcoal is identified because of its association with relative wealth. Consider terminology such as “wealth-validated higher-status cooking category” rather than “improved” or “clean,” unless supported by an external health or environmental standard.

**4. Explain the choice of the zero/sample-mean cut-point.** Section 3.3 defines the validated improved set as categories with adjusted mean wealth above zero. Please provide a clearer theoretical or empirical justification for this threshold. A brief sensitivity analysis using alternative thresholds would further strengthen the approach.

**5. Expand reporting of the PCA wealth anchor.** The Methods section should provide basic information on PCA construction, including the variance explained by the first component and, preferably, principal component loadings or a supplementary table. This would improve transparency and reproducibility.

**6. Clarify the bootstrap procedure.** Please briefly specify how survey weights, strata, and enumeration-area clustering were handled during bootstrap resampling, particularly given the complex survey design.

**7. Address very small category sizes more explicitly.** Some categories contain very few observations, including multi-storey houses, tents, villas, and wooden houses. Although the manuscript acknowledges this limitation and uses bootstrap intervals, the authors should state whether a minimum category-size rule is advisable for future WACV applications.

**8. Strengthen the external validity statement.** Because the empirical application is based on one wave of one fragile-state survey, the Discussion should emphasize that the findings demonstrate the method rather than establish the prevalence of coding errors across fragile states. Validation using additional survey waves or countries should be identified as an important future research direction.

**9. Improve reproducibility and data/code availability.** The submission states that the analysis code will be deposited in OSF/Zenodo upon acceptance, whereas the manuscript states that it is available from the author. These statements should be made consistent. Providing a public repository and persistent identifier during revision, where possible, would strengthen reproducibility.

**10. Undertake a final editorial check.** Please ensure consistent terminology throughout the manuscript, particularly for “miscoding,” “mis-ranking,” “improved,” “clean,” and “wealth-validated.” Figure captions should also be fully self-explanatory, including definitions of marker colours and confidence intervals.

**Overall Recommendation: Minor Revision**

The manuscript presents a useful and practically relevant methodological contribution and is generally well executed. The requested revisions do not require fundamental changes to the study design or principal analyses. They primarily concern conceptual clarification, methodological transparency, cautious interpretation of wealth-based classifications, reproducibility, and presentation. Subject to satisfactory attention to these points, the manuscript would be suitable for further consideration for publication in PLOS ONE.
