# Supplementary material for: Wealth-anchored category validation: A diagnostic for categorical orderings in fragile-state household surveys
Source: PLoS One. 2026 Oct 29;21(10):e0360752. doi: 10.1371/journal.pone.0360752 (PMC13718975; doi:10.1371/journal.pone.0360752)
Supplement: Supplementary file 6 [file pone.0360752.s007.docx]

Manuscript	[Click here to access/download;Manuscript;Manuscript.docx](https://www2.cloud.editorialmanager.com/pone/download.aspx?id=45845121&guid=440876d9-66de-436c-9a7a-471f6ec49b37&scheme=1)[<https://www2.cloud.editorialmanager.com/pone/download.aspx?id=45845121&guid=440876d9-66de-436c-9a7a-471f6ec49b37&scheme=1>](https://www2.cloud.editorialmanager.com/pone/download.aspx?id=45845121&guid=440876d9-66de-436c-9a7a-471f6ec49b37&scheme=1)

1	**Wealth-Anchored Category Validation: A Diagnostic for Categorical**

2	**Orderings in Fragile-State Household Surveys**

3	*Short title: A diagnostic for categorical orderings in fragile-state surveys*

4	Gudi V. Chandra Sekhar

5	Department of Economics, College of Business and Economics, Gambella University, Gambella, Ethiopia

6	* Corresponding author. E-mail: chandragudi@gmu.edu.et

7	**Abstract**

8	Asset-based wealth indices and binary "improved" or "clean" service indicators both assume that the

9	response options of a categorical survey variable are coded in the wealth order they imply. The

10	assumption is rarely tested. I set out a procedure, Wealth-Anchored Category Validation (WACV), that

11	tests it, and I am careful about what a failed test means. WACV ranks a variable's categories by their

12	average position on a wealth index built from the other indicators, so no variable is judged against itself,

13	and attaches stratified cluster-bootstrap intervals in which the whole estimation chain, including the index

14	itself, is re-estimated in every replication. It reports two things: whether the categories sit on the local

15	wealth gradient in the order the coding assumes, and whether a binary someone has already built from the

16	variable falls where that gradient actually steps. Divergence on either is a diagnostic signal with several

17	possible explanations: a coding fault, an analytical recoding fault, a context-dependent classification, or

18	noise. The procedure does not adjudicate between them from wealth data alone. Applied to five ordinal

19	variables in three waves of the South Sudan High Frequency Survey, the ordering diagnostic finds no

20	robust inversion in the main specification; equivalence tests and a simulation locate what that null can and

21	cannot rule out. The cut-point diagnostic finds one severe anomaly: an improved-housing binary used in

22	earlier analysis of these data places the two poorest dwelling types on the improved side, and sits 0.26

23	standard deviations below the households it excludes. That anomaly replicates in a survey wave not used

24	to develop the method. A codebook audit shows the binary originates in analysis rather than in the survey

25	instrument, which makes it an instructive case of analytical recoding rather than a defect in the data.

26	**1. Introduction**

27	Two instruments carry much of the weight in welfare measurement where consumption data are missing

28	or distrusted. One is the asset-based wealth index, which compresses a set of ownership and amenity

29	indicators into a single ranking of households [1–7]. The other is the binary service indicator, such as

30	improved water, improved sanitation or clean cooking fuel, which collapses a categorical survey question

31	into an adequate/inadequate split [8–10]. Both rest on a premise that is almost never examined on its own

32	terms: that the response options of the underlying categorical variable have been coded in an order that

33	corresponds to the welfare ordering the analyst intends to capture.

34	This paper takes that premise as an empirical question. The procedure I describe, Wealth-Anchored

35	Category Validation (WACV), ranks the categories of a variable by their average position on a wealth

36	index constructed from the other indicators, so that no variable is ever judged against itself. Where the

37	resulting order diverges from the order the coding assumes, or where a binary drawn from the variable

38	turns out to fall somewhere the wealth gradient does not step, the procedure flags the variable for

39	investigation.

40	What follows from a flag is the part of this paper that has changed most in revision, and it is worth stating

41	plainly at the outset. A divergence between an assigned ordering and the local wealth ordering is not

42	proof that the assigned ordering is wrong. Internationally standardised categories such as "clean cooking

43	fuel" and "improved sanitation" describe technical, health and infrastructural properties. They were never

44	claims about relative prosperity, and a category can sit high on a local wealth gradient without meeting

45	them. Charcoal is the clearest instance: in urban South Sudan it is plausibly the fuel of comparatively

46	well-off households, and it is not a clean fuel by any combustion or health standard. A wealth anomaly is

47	a reason to look at a variable. It is not a verdict on it.

48	WACV therefore reports two diagnostics and adjudicates neither. The ordering diagnostic asks whether

49	categories sit on the wealth gradient in the order the coding assumes. The cut-point diagnostic asks

50	whether a binary indicator that someone has already constructed falls where the gradient actually steps.

51	The second evaluates a cut; it never proposes one. No "validated improved set" is constructed anywhere

52	in this paper, and no threshold relative to the sample mean is used.

53	The empirical application is to the South Sudan High Frequency Survey, a stratified two-stage survey of a

54	fragile, largely rural, overwhelmingly poor population. Three results follow. First, in the main

55	specification the ordering diagnostic finds no robust inversion in any of five ordinal variables (housing

56	type, sanitation, drinking water, lighting and cooking fuel) once the design is honoured, the anchor's own

57	uncertainty is propagated, multiplicity is controlled, and categories too thin to support inference are set

58	aside. A simulation study says what that null can and cannot rule out. Second, the cut-point diagnostic

59	finds one severe anomaly: an improved-housing binary used in earlier analysis of these data assigns the

60	improved label to tents and stick-walled tukuls, and the households it labels improved sit 0.26 standard

61	deviations below those it excludes. That anomaly replicates, at almost identical magnitude, in a survey

62	wave that played no part in developing the method. Third, an audit of the survey instrument and its

63	codebook shows that this binary has no basis in the survey's own coding; it was created during analysis.

64	The case is therefore an example of analytical recoding gone wrong, not of a defective survey, and that

65	distinction is one the paper now draws explicitly.

66	The contribution is methodological. WACV is cheap, requires no data beyond the survey itself, and

67	belongs as a screening step in the wealth-index and multidimensional-poverty pipelines, where

68	categorical orderings and binary cut-points are routinely imported from one survey context into another.

69	The substantive lesson is narrower than the one the first version of this paper drew, and more defensible:

70	an imported cut-point can run backwards against the local wealth gradient, and whether it does is an

71	empirical matter worth checking before the indicator is trusted.

72	**Materials and methods**

73	**2. What a divergence can mean**

74	Because the interpretation of a flag is the crux of the method, this section fixes terminology before any

75	estimation is described. Four distinct things can produce a divergence between an assigned categorical

76	ordering and the local wealth ordering, and the literature on measurement error in asset indices does not

77	consistently distinguish them [11–14]. Table 1 sets the four out.

78	**Table 1. Four sources of divergence between an assigned ordering and the local wealth ordering.**

| **Source** | **What has gone wrong** | **Where it lives** | **Can wealth data alone identify it?** |
| --- | --- | --- | --- |
| Coding error | The survey instrument or its codebook assigns response options to categories incorrectly, or the recorded codes do not match the documented  ones. | The survey | No; requires the questionnaire and codebook |
| Recoding error | The instrument is sound, but a derived variable built by the analyst maps the raw categories wrongly. | The analysis | No; requires the analysis code and its documentation |
| Context-  dependent  classification | Both instrument and derived variable are correct on their own terms; the global standard simply does not track prosperity in this population. | Neither; the standard is doing a different job | No; requires the technical or health definition |
| Sampling noise | Nothing is wrong; the apparent divergence is within the range the design's uncertainty permits. | Nowhere | Yes; this is what the inference is for |

79	Note. WACV separates the fourth from the first three by inference. Separating the first three from one another requires the survey

80	documentation, the analysis code, or the technical standard, and the paper does that separation explicitly for the case it examines.

81	The practical consequence is that the procedure's output is a flag plus a shortlist of explanations, and that

82	resolving the flag is documentary work, not statistical work. In the application below, the housing

83	anomaly is resolved as a recoding error by reading the questionnaire and the codebook; the cooking-fuel

84	anomaly is resolved as a context-dependent classification by observing what the clean-fuel standard is for;

85	and three variables produce no flag at all, which the inference section is careful not to call confirmation.

86	This also fixes the scope of the paper's claims. The evidence here comes from one survey programme in

87	one country, across three waves. It is not a comprehensive validation of any indicator definition, and

88	nothing in it licenses a general claim about how well international service standards track wealth

89	elsewhere.

90	**3. The procedure**

91	*3.1 The anchor*

92	Let a household i carry a vector of wealth indicators. For a categorical variable under test, every indicator

93	derived from that variable is removed from the pool, and a survey-weighted first principal component is

94	extracted from what remains [1,15]. The component is standardised to weighted mean zero and unit

95	weighted standard deviation and oriented so that it correlates positively with the count of durable asset

96	types owned, which fixes the sign of the index rather than leaving it to the arbitrary sign convention of the

97	eigen-decomposition.

98	The weighting matters and was not handled consistently in the first version of this work. Means, standard

99	deviations and the correlation matrix from which the component is extracted are all computed with survey

100	weights, as are the category means that later use the index. An unweighted anchor feeding weighted

101	category means treats the design as relevant to one step and irrelevant to the previous one.

102	Leave-one-out removes mechanical circularity: a variable cannot help build the index against which it is

103	judged. It does not make the exercise independent of the dataset on which the method was developed, and

104	the paper does not claim that it does. Independence of that stronger kind is what Sections 6 and 7 are for.

105	*3.2 Location adjustment*

106	Wealth in this setting varies sharply between states and between urban and rural areas, and the

107	composition of a category differs across those cells. The index is therefore residualised on urban/rural

108	status and state by survey-weighted least squares, and category positions are computed on the residual.

109	Four alternative specifications (state only, urban only, the full stratum interaction, and no adjustment) are

110	reported in Section 8.

111	*3.3 The ordering diagnostic*

112	For each category k, the weighted mean of the location-adjusted index is computed among households in

113	that category. The categories are then compared pairwise. For every pair the difference in means is taken

114	directly from the bootstrap distribution described below, and a confidence interval is reported for the

115	difference itself. The non-overlap of two separate marginal intervals is not used as a test anywhere in this

116	paper; it is not one.

117	A pair is recorded as a robust inversion when the category the coding places higher sits significantly

118	lower on the wealth gradient, judged against the difference distribution. Because five variables generate a

119	large family of pairwise comparisons, the false discovery rate across the entire family is controlled by the

120	Benjamini–Hochberg procedure at q = 0.05 [16].

121	Two summary statistics accompany the pairwise results. The headline is a frequency-weighted

122	discordance ratio: the share of category pairs, weighted by the product of the two categories' population

123	shares, whose wealth order contradicts the assumed order. This is reported alongside the weighted share

124	of households sitting in a category involved in at least one robust inversion. Spearman's rank correlation

125	between the assumed and observed orderings is retained but demoted to a secondary statistic, because it

126	weights a two-household category as heavily as a nine-hundred-household one and can therefore stay high

127	while a populous inversion is present.

128	*3.4 The cut-point diagnostic*

129	Binary indicators are the form in which categorical variables usually reach applied work, and a binary can

130	be misplaced even when the ordering behind it is sound. For a binary defined by a set S of categories, the

131	diagnostic computes the weighted difference in location-adjusted wealth between households inside S and

132	those outside it, with a bootstrap interval. A negative difference means the indicator is upside down: the

133	households it labels adequate sit lower on the wealth gradient than the ones it excludes.

134	This diagnostic evaluates a cut that someone else has drawn. It does not propose a replacement, and it

135	involves no threshold defined relative to the sample. That is a deliberate change from the first version of

136	this work, in which categories whose adjusted mean exceeded the sample mean of zero were assembled

137	into a "validated improved set". That rule is indefensible in a population where roughly four households

138	in five live below the international extreme-poverty line: being above the local average is not evidence of

139	adequacy in any technical sense, and in these data the rule would have classified an open-water drinking

140	source as improved. It has been removed rather than repaired.

141	*3.5 The precision floor*

142	Several categories in surveys ofthis kind contain a handful of households. A category with one household

143	yields a bootstrap interval whose endpoints coincide. That is a point, not an interval, and it is the opposite

144	of the signal that thin evidence should produce. A single rule is therefore fixed in advance and applied to

145	every variable, every wave and every robustness run: a category is always reported, but is classified, and 146	admitted to the pairwise family, only if it contains at least 30 households in at least 5 enumeration areas.

147	Section 8 reports what happens at three alternative floors.

148	*3.6 Inference*

149	Uncertainty comes from a stratified cluster bootstrap that mirrors the survey design [17]. Within each of

150	the design strata, that stratum's enumeration areas are resampled with replacement; households travel with

151	their area. Two features of the implementation deserve emphasis because both were wrong in the first

152	version of this work.

153	First, enumeration areas are identified by the pair (stratum, area), not by the raw area identifier. In these

154	files the area identifier is unique only within stratum: each of its 26 values recurs across three to seven

155	different strata. Factorising it alone collapses a design of 156 enumeration areas into 26 pseudo-clusters, 156	each pooling households drawn from several states, and resamples those.

157	Second, the entire estimation chain is re-estimated inside every replication: the standardisation, the

158	principal component, the location residualisation and the category means. Fixing the index outside the

159	loop and resampling only pre-computed aggregates treats the anchor as known when it is estimated, and

160	understates uncertainty in every quantity that depends on it. The main results of Sections 5 and 6, and the

161	alternative anchors of Section 8.1, use 2,000 replications. The precision-floor and tolerance sensitivities

162	of Sections 8.2 and 8.3 use 1,500; the 42-run robustness suite of Section 8.1 uses 600 per run; and the

163	simulation of Section 7 uses 300 per synthetic dataset. Each count is fixed in the deposited run script, and

164	the deposited outputs reproduce every number in this paper to the last decimal when it is re-run.

165	*3.7 Reporting a negative result*

166	Where no robust inversion is found, the paper reports that no robust evidence of an inversion was

167	detected. It does not report that the coding has been cleared, confirmed or validated. The distinction is not

168	pedantic: a variable can fail to produce a flag because its coding is sound, or because the categories

169	concerned are too thin or the design too coarse for any inversion to have shown up.

170	Two devices are used to give the negative results content. Adjacent pairs are subjected to two one-sided

171	tests [18] against a tolerance of 0.20 standard deviations, so that a pair whose difference interval lies

172	wholly inside that band can be described as equivalent within tolerance rather than merely undetected.

173	And the simulation in Section 7 supplies power curves, which state what size of inversion the design

174	would have found at each category frequency. A negative result accompanied by neither of these is

175	reported as inconclusive, and several are.

176	**4. Data**

177	*4.1 The survey and its design*

178	The South Sudan High Frequency Survey was conducted by the World Bank with South Sudan's National

179	Bureau of Statistics, funded by the UK Department for International Development [19,20]. The main

180	analysis uses Wave 3, collected between September 2016 and March 2017 across seven of the country's

181	ten former states: Western Equatoria, Central Equatoria, Eastern Equatoria, Northern Bahr-el-Ghazal,

182	Western Bahr-el-Ghazal, Warrap and Lakes. The remaining three states could not be surveyed for security

183	reasons.

184	The design is stratified and two-stage. Strata are formed by state crossed with urban/rural status, giving 14

185	strata. Within each stratum, enumeration areas are the primary sampling units and were drawn with

186	probability proportional to size, estimated from the 2008 Census; within each selected area a listing was

187	conducted and 12 households drawn at random. Sampling weights are the inverse probability of selection, 188	decomposed into the area's selection probability and the household's selection probability within the area, 189	and scaled to the number of households per stratum from the Census. Enumeration areas were replaced

190	where security made fieldwork unfeasible, and households were replaced after three unsuccessful visits.

191	The analysis sample contains 1,828 to 1,839 households depending on the variable under test, spread over

192	156 enumeration areas in 14 strata.

193	Waves 1 and 2 are used for out-of-sample validation in Section 6. Wave 1 was collected between

194	February and September 2015 and contains 3,550 households in 300 enumeration areas across 12 strata.

195	Wave 2 was collected between February and June 2016, revisited the urban households of Wave 1 and

196	added areas in four states, and contains 1,189 households in 101 enumeration areas.

197	*4.2 Variables*

198	Five ordinal variables are tested: dwelling structure, toilet facility, main drinking-water source, main

199	lighting source and main cooking-fuel source. Four of these reach the analyst through derived variables

200	supplied in the survey's own clean output, which collapse the raw questionnaire responses into ordered

201	categories. Dwelling structure is the exception: it is a plain ten-category question with no derived

202	counterpart and no improved/unimproved split of any kind in the survey materials.

203	The anchor pool holds eight indicators: improved housing, improved water, improved sanitation,

204	electricity for lighting, clean cooking, a count of durable asset types owned, room density, and land

205	ownership. Every indicator is constructed in one place in the deposited code and nowhere else. For each

206	variable under test, all indicators derived from that variable are removed from the pool.

207	*4.3 Provenance of the improved-housing binary*

208	Because the housing case is the paper's principal application, and because both referees asked where the

209	classification came from, the survey documentation was examined directly. The finding is unambiguous

210	and it changes how the case must be described.

211	The questionnaire item records the structure a household inhabits in ten labelled categories: tent, stick

212	tukul, mud tukul, flat or apartment, villa, one-floor mud house, one-floor brick or concrete house, wooden

213	house, multi-storey house, and incomplete structure. The survey defines no improved/unimproved split

214	over these categories. The World Bank's derived variables cover water, sanitation, lighting and cooking;

215	there is no derived housing variable. Both the original improved set and the corrected set used in earlier

216	analysis of these data were therefore created during analysis, not supplied by the survey.

217	One detail of the coding is worth recording because it suggests a mechanism. The category codes run 1, 3, 218	4, 5, 6, 7, 8, 9, 10, 11; there is no code 2. A rule expressed as "types 1 to 3" therefore selects tents and

219	stick tukuls, and nothing else: 478 households in the full Wave 3 file, 25.9 percent unweighted, and the

220	two poorest dwelling types in the survey. (In the analysis sample used in Section 5, which drops

221	households with a missing anchor indicator, the same set holds 475 households and a survey-weighted

222	29.2 percent.) Many household survey codebooks list the most substantial dwelling first; this one lists the

223	least substantial first. A rule imported from a survey with the opposite convention would select precisely

224	this set. Under the terminology of Section 2 this is a recoding error, located in the analysis rather than in

225	the survey, and the paper describes it that way throughout.

226	*4.4 Ethics statement*

227	This study is a secondary analysis of de-identified, publicly archived household survey microdata

228	obtained from the World Bank Microdata Library. The author collected no data from human participants

229	and had no contact with respondents. The datasets are distributed as anonymised Public Use Datasets

230	from which direct identifiers have been removed by the data producer. Because the research involved no

231	interaction with human subjects and no access to identifiable private information, no institutional review

232	board approval was required, and none was sought; Gambella University's research ethics committee does

233	not require review for secondary analysis of anonymised public-use data. Informed consent was obtained

234	from participants by the original data collectors, the World Bank and South Sudan's National Bureau of

235	Statistics, at the time of the original surveys. The author accessed the data under the World Bank

236	Microdata Library's standard terms for public use datasets, which require agreement not to attempt re-

237	identification of any respondent.

238	**Results**

239	**5. The two diagnostics in Wave 3**

240	*5.1 The ordering diagnostic*

241	Table 2 reports the ordering diagnostic for all five variables in Wave 3. No robust inversion survives in

242	any of them. Across the five variables, 21 pairwise comparisons are admitted by the precision floor, and

243	none is a significant inversion after control of the false discovery rate. Every pairwise comparison,

244	whether or not it clears the floor, is listed with its difference, interval and adjusted p-value in S2 Table.

245	**Table 2. Ordering diagnostic, Wave 3, main anchor.**

| **Variable** |   **N** | **Categories** | **Classifiable** | **Pairs**  **tested** | **Robust inversions** | **Weighted discordance** | **Spearman ρ** |
| --- | --- | --- | --- | --- | --- | --- | --- |
| Dwelling  structure | 1,838 | 10 | 4 | 6 | 0 | 0.012 | 0.84 |
| Sanitation | 1,828 | 4 | 2 | 1 | 0 | 0.003 | 0.80 |
| Drinking water | 1,839 | 4 | 3 | 3 | 0 | 0.016 | 0.80 |
| Lighting | 1,838 | 5 | 5 | 10 | 0 | 0.290 | 0.90 |
| Cooking fuel | 1,839 | 4 | 2 | 1 | 0 | 0.002 | −0.20 |

246	Note. 2,000 stratified cluster-bootstrap replications; 156 enumeration areas within 14 strata; the full estimation chain re-estimated

247	in every replication; Benjamini–Hochberg control at q = 0.05 across the whole family of pairwise comparisons. Spearman's ρ is

248	reported for comparability with earlier work and is not used to draw any conclusion.

249	Fig 1 shows the same result category by category. The point to take from it is the colour: in dwelling

250	structure, every category the design can classify falls in ascending order, and every category out of order

251	sits below the precision floor.

252	Fig 1. Location-adjusted wealth position of each response category, by variable, Wave 3. Markers give the weighted mean

253	position of each category on the anchor, in standard-deviation units, with 95 percent stratified cluster-bootstrap intervals

254	from 2,000 replications in which the entire estimation chain is re-estimated. Categories are ordered by observed wealth

255	position, not by their assigned codes. Dark markers denote categories that clear the pre-specified precision floor of 30

256	households in at least 5 enumeration areas and are therefore admitted to the pairwise comparisons; pale markers denote

257	categories that are reported but never classified. N = 1,828 to 1,839 depending on the variable.

258	Two features of this table need comment. First, the dwelling-structure ordering is monotonic among the

259	categories large enough to classify: stick tukul at −0.18, mud tukul at −0.02, mud house at +0.23, brick

260	house at +0.78. The apparent disorder in the full ten-category ranking is carried entirely by categories

261	holding between two and twenty-two households (flat or apartment, villa, wooden house, multi-storey

262	house, tent and incomplete structure), for which the design supports no conclusion. Second, cooking fuel

263	returns a Spearman ρ of −0.20, which looks alarming and means very little: it is computed over four

264	categories, two of which contain seventeen and one households respectively. The frequency-weighted

265	discordance ratio for the same variable is 0.002. This is exactly the contrast that motivates demoting ρ .

266	*5.2 The cut-point diagnostic*

267	Table 3 turns to the binaries. Six of the seven cuts examined behave as intended. One does not, and one

268	cannot be estimated at all.

269	**Table 3. Cut-point diagnostic, Wave 3, main anchor.**

| **Binary indicator** | **n inside** | **Weighted share** | **Wealth gap (SD)** | **95% CI** | **Verdict** |
| --- | --- | --- | --- | --- | --- |
| Improved housing, original analytical coding (types 1–3) | 475 | 29.2% | −0.263 | [−0.33, −0.17] | Inversion: the labelled side sits lower |
| Improved housing, corrected coding (types 5, 8, 9, 10) | 156 | 4.4% | +0.881 | [+0.60, +1.14] | Ordered as intended |
| Clean cooking fuel, conventional definition (gas, electricity, biogas) | 1 | 0.0% | — | — | Not estimable |
| Cooking-fuel cut placed one category lower, at charcoal | 385 | 10.4% | +0.749 | [+0.53, +0.91] | Ordered as intended |
| Improved drinking water (derived variable) | 307 | 11.0% | +0.324 | [+0.22, +0.42] | Ordered as intended |
| Improved sanitation (derived variable) | 783 | 27.2% | +0.345 | [+0.24, +0.43] | Ordered as intended |

| **Binary indicator** | **n inside** | **Weighted share** | **Wealth gap (SD)** | **95% CI** | **Verdict** |
| --- | --- | --- | --- | --- | --- |
| Electricity for lighting (derived variable) | 82 | 2.5% | +1.453 | [+1.07, +1.84] | Ordered as intended |

270	Note. The wealth gap is the weighted difference in location-adjusted wealth between households the binary labels adequate and

271	all others, in anchor standard-deviation units. Estimates are withheld where the precision floor is not met.

272	The original improved-housing binary is inverted, decisively. The households it labels improved sit 0.26

273	standard deviations below those it excludes, with an interval nowhere near zero. The reason is visible in

274	the composition: the labelled set consists oftents and stick tukuls. The corrected coding used in later

275	analysis of these data points the right way, at +0.88, which is what one would expect of a set consisting of

276	flats, brick houses, wooden houses and multi-storey houses. The diagnostic, however, certifies that set no

277	more than it certifies any other, and Section 9 returns to what it does and does not license.

278	The conventional clean-fuel binary is the more instructive case. In Wave 3 it captures a single household.

279	It is not that the indicator marks a small share of the population; it is that no estimate of its position is

280	possible at the precision this paper requires of itself, and the honest report is that the cut cannot be

281	evaluated. Meanwhile the one large step in the cooking-fuel variable sits a category lower. Moving the cut

282	to charcoal produces a gap of +0.75 standard deviations, precisely estimated.

283	It bears repeating that this is not an argument that charcoal is a clean fuel. It is not, and the clean-fuel

284	standard is not attempting to measure prosperity. The finding is that in this population the cooking-fuel

285	variable's information about relative wealth is concentrated at a boundary the clean-fuel indicator does not

286	use, so an analyst who reaches for that indicator as a proxy for household living standards will capture

287	almost nothing. Under the terminology of Section 2 this is a context-dependent classification, and the

288	appropriate response is to stop using the indicator as a wealth proxy, not to redefine clean cooking.

289	*5.3 Where the gradient steps*

290	The adjacent-step profile makes the point in Table 3 general. Along the assumed ordering of each

291	variable, most adjacent steps are indistinguishable from zero and a few are large. In lighting, the step from

292	grass and firewood to lamp, candle or torch is +0.39 and the step from there to electricity is +1.07, while

293	the step from electricity to solar or biogas (+0.23) cannot be distinguished from zero and the step from no

294	lighting to grass and firewood (−0.05) is equivalent within tolerance. In cooking fuel, the single step from

295	firewood to charcoal is +0.75 and carries the variable's entire wealth signal. In drinking water, the step

296	from purchased to piped supply is −0.02 and is simply inconclusive.

297	This is a useful output in its own right. An analyst choosing where to cut a categorical variable for a

298	wealth-related purpose can read the steps directly, rather than importing a boundary drawn for a different

299	purpose in a different setting.

300	**6. Out-of-sample application**

301	The method was developed after an anomaly was noticed in Wave 3, and that same anomaly cannot then

302	serve as confirmation that the method works. The estimator was therefore applied, without modification, 303	to two waves that played no part in its development.

304	Two constraints are recorded rather than worked around. Wave 1 does not contain the dwelling-structure

305	question, so the housing tests run on Wave 2 only. Wave 2's public file carries neither a stratum variable

306	nor an urban indicator, so its bootstrap is stratified by state and its location adjustment uses state alone.

307	Table 4 reports the cut-point diagnostic for every binary in both waves.

308	**Table 4. Cut-point diagnostic out of sample.**

| **Wave** |   **Binary indicator** | **n inside** | **Wealth gap (SD)** | **95% CI** | **Verdict** |
| --- | --- | --- | --- | --- | --- |
| 2 | Improved housing, original analytical  coding | 241 | −0.277 | [−0.41, −0.10] | Inversion: the labelled side sits lower |
| 2 | Improved housing, corrected coding | 178 | +0.713 | [+0.54, +0.87] | Ordered as intended |
| 1 | Clean cooking fuel, conventional definition | 0 | — | — | Not estimable |
| 2 | Clean cooking fuel, conventional definition | 3 | — | — | Not estimable |
| 1 | Cooking-fuel cut at charcoal | 947 | +0.512 | [+0.31, +0.68] | Ordered as intended |
| 2 | Cooking-fuel cut at charcoal | 668 | +0.663 | [+0.49, +0.77] | Ordered as intended |
| 1 | Improved sanitation | 1,623 | +0.404 | [+0.26, +0.52] | Ordered as intended |

| **Wave** |   **Binary indicator** | **n inside** | **Wealth gap (SD)** | **95% CI** | **Verdict** |
| --- | --- | --- | --- | --- | --- |
| 2 | Improved sanitation | 859 | +0.620 | [+0.42, +0.74] | Ordered as intended |
| 1 | Electricity for lighting | 205 | +0.983 | [+0.71, +1.19] | Ordered as intended |
| 2 | Electricity for lighting | 99 | +1.033 | [+0.69, +1.39] | Ordered as intended |
| 1 | Improved drinking water | 342 | +0.394 | [−0.13, +0.66] | Inconclusive |
| 2 | Improved drinking water | 95 | +0.189 | [−0.20, +0.56] | Inconclusive |

309	Note. Wave 1 does not contain the dwelling-structure question, so the two housing rows appear for Wave 2 only.

310	The housing inversion replicates. Wave 2 returns −0.277 standard deviations, against −0.263 in Wave 3:

311	the same sign and effectively the same magnitude, in a wave the method never saw. The failure of the

312	conventional clean-fuel binary replicates too, and more starkly: it captures no households at all in Wave 1

313	and three in Wave 2. The water cut is inconclusive in both waves, which is a limitation of the data rather

314	than a finding about the indicator.

315	Fig 2 assembles the cut-point diagnostic for every binary, in every wave in which it can be evaluated. One

316	indicator sits alone to the left of zero, and it does so in both of its waves.

317	Fig 2. A binary whose labelled side sits left of zero is upside down. Each marker gives the weighted difference in location-

318	adjusted wealth between the households a binary indicator labels adequate and all other households, in anchor standard-

319	deviation units, with a 95 percent stratified cluster-bootstrap interval. A binary whose interval lies to the left of zero is

320	inverted: the households it labels adequate sit lower on the wealth gradient than those it excludes. The improved-housing

321	binary built during earlier analysis of these data is the only such case, and it appears in both waves in which it can be

322	evaluated. The conventional clean-fuel binary cannot be estimated in any wave, capturing between zero and three

323	households. Wave 1 does not contain the dwelling-structure question.

324	The ordering diagnostic agrees across waves everywhere except one pair (S3 Table lists every out-of-

325	sample comparison), and that exception is reported here rather than smoothed away. In Wave 2 the

326	lighting variable shows one robust inversion: households reporting no lighting at all sit above those using

327	grass or firewood (−0.20 against −0.78, adjusted p = 0.008), where the coding places them below. The

328	same pair runs in the same direction in Wave 3 (−0.12 against −0.17), but the gap there is small enough to

329	be declared equivalent within tolerance. Consistent direction across waves, differing magnitude, differing

330	verdict. Section 8 shows the same pair crossing the threshold in Wave 3 under several perturbations of the

331	survey-indicator anchor, and reversing sign under the consumption anchor. The safest reading is that these

332	data do not resolve the pair, and that a purpose-built instrument would be needed to do so.

333	**7. Simulation**

334	An out-of-sample wave establishes that the procedure behaves consistently. It cannot establish that the

335	procedure recovers an inversion that is genuinely present, because in real data the truth is unknown. For

336	that, a synthetic population was constructed in which the ordering is known by construction and an

337	inversion of known size is planted between a known pair of categories.

338	The population reproduces the survey's design features: 14 strata, clustered enumeration areas of twelve

339	households, unequal sampling weights, and anchor indicators generated as noisy functions ofa latent

340	wealth variable together with an urban location effect that the residualisation is meant to remove. The grid

341	varies the sample size, the population share of the category carrying the planted inversion, and the size of

342	the inversion in standard-deviation units. Each of the 32 cells was replicated 150 times with 300 bootstrap

343	replications, for 4,800 simulated datasets in total. Because a large planted swap can also invert that

344	category against others, those additional genuine inversions are scored as true rather than false; scoring

345	them as false positives would flatter the procedure's specificity. Table 5 reports the full grid.

346	**Table 5. Simulation: detection of planted category inversions.**

| **N** | **Category share** | **Mean**  **category n** | **False positives at Δ = 0** | **Power at Δ = 0.2** | **Δ = 0.4** | **Δ = 0.8** | **Δ at 80% power** |
| --- | --- | --- | --- | --- | --- | --- | --- |
| 1,800 | 2% | 36 | 0.045 | 0.20 | 0.52 | 0.98 | 0.65 |
| 1,800 | 5% | 90 | 0.040 | 0.31 | 0.79 | 1.00 | 0.41 |
| 1,800 | 10% | 180 | 0.020 | 0.53 | 0.97 | 1.00 | 0.32 |
| 1,800 | 25% | 450 | 0.033 | 0.61 | 0.99 | 1.00 | 0.30 |
| 3,550 | 2% | 71 | 0.020 | 0.29 | 0.79 | 1.00 | 0.41 |
| 3,550 | 5% | 177 | 0.033 | 0.57 | 0.97 | 1.00 | 0.32 |
| 3,550 | 10% | 356 | 0.027 | 0.77 | 1.00 | 1.00 | 0.23 |
| 3,550 | 25% | 886 | 0.060 | 0.91 | 1.00 | 1.00 | 0.17 |

347	Note. Δ is the size of the planted inversion in anchor standard-deviation units. The false-positive column reports how often the

348	planted pair itself is flagged when its displacement is zero. Specificity is computed over every other testable pair, whose order is

349	concordant by construction; it was 1.000 in every cell, so no such pair was flagged in any of the 4,800 datasets. The final column

350	interpolates the smallest inversion the design recovers in four runs out of five.

351	Fig 3 traces the resulting power curves.

352	Fig 3. Power to recover a planted category inversion, by sample size, category frequency and inversion size. Each point is

353	the share of 150 simulated datasets in which the procedure recovers an inversion planted between a known pair of

354	categories, after Benjamini–Hochberg control across the pairwise family. The dashed line marks 80 percent power; the

355	dotted line marks the nominal 5 percent false-positive rate, against which the points at zero inversion size should be read.

356	Specificity, computed over every testable pair other than the planted one, was 1.000 in every cell: across all 4,800

357	simulated datasets, no such pair was ever flagged.

358	Three things follow. The procedure is correctly calibrated: with no inversion planted it flags the target

359	pair between 2.0 and 6.0 percent of the time across the eight cells, against a nominal 5 percent, and the

360	pooled rate over all cells is 3.5 percent. It is highly specific over the remaining pairs: across 4,800

361	datasets it never once flagged a pair other than the planted one. And its power depends, as one would

362	expect, on how many households sit in the categories being compared. At a sample the size of Wave 3, an

363	inversion of roughly 0.30 standard deviations is recovered four times in five for a category holding a

364	quarter of households, rising to about 0.65 standard deviations for a category holding two percent.

365	This is what allows the null results of Section 5 to be stated as something more than an absence of

366	evidence. At Wave 3's size, an ordering inversion smaller than about a third of a standard deviation in the

367	common categories, or two-thirds in the thin ones, would plausibly have escaped detection. Inversions

368	larger than that would not have. The negative result is bounded, and the bound is reported rather than

369	asserted.

370	**8. Robustness and analysis choices**

371	*8.1 Anchor and specification*

372	Forty-two robustness runs were carried out and are reported in full in S1 Table: each of the eight anchor

373	indicators dropped in turn; every pair of them dropped in turn; five location-adjustment specifications;

374	and an iterated second pass in which any anchor indicator whose source variable showed a robust

375	inversion on the first pass would be rebuilt from the observed wealth ordering and the analysis re-run.

376	Thirty-seven of the forty-two reproduce every baseline verdict exactly. All eight drop-one runs pass. All

377	five location specifications pass, including no location adjustment at all. The iterated second pass is a no-

378	op, because no variable showed a robust inversion on the first pass and so no anchor indicator required

379	rebuilding; this is reported as a fact about this application rather than as a check that happened to pass.

380	The five drop-two runs that depart from the baseline all do so in the same way: the lighting pair discussed

381	in Section 6 crosses the significance threshold when the anchor is reduced to six indicators. No other

382	verdict moves anywhere in the suite. Under the consumption anchor introduced below, that same pair

383	runs the other way, with grass and firewood above no lighting by 0.23 standard deviations (adjusted p = 384	0.004), which is the order the coding assumes; this is the strongest reason not to read the Wave 2 result as

385	a feature of the population.

386	Three further anchors address the concern that a pool of service indicators drawn from the same survey

387	may share a common local structure that reinforces itself. The first uses only durable assets, room density

388	and land ownership, and so contains no normatively coded service indicator at all. The second substitutes

389	the original housing coding and the conventional clean-fuel coding for the corrected versions, so that no

390	indicator adjusted in the light of these data enters the pool. The third leaves the asset and service block

391	altogether: it is the logarithm of the survey's own per-capita daily consumption aggregate, standardised

392	and location-adjusted in the same way as the composite index, and it is the external measure of well-being

393	these files contain. Because it is a single variable rather than a composite, leave-one-out is vacuous for it;

394	and because consumption is measured with recall error over a short reference period, its correlation with

395	the durable-asset count is only 0.38, so it is a noisier yardstick than the other three.

396	All three reproduce the ordering verdicts ofTable 2: no robust inversion in any variable on any anchor.

397	Table 6 reports the cut-point diagnostic under each. The asset-only and pre-correction anchors reproduce

398	Table 3 in every row, and the housing inversion is if anything sharper under them, at −0.33 and −0.30

399	standard deviations. Under the consumption anchor every gap is attenuated, as a noisier anchor implies, 400	and the attenuation is close to uniform: the corrected housing binary, the charcoal cut, the sanitation

401	binary and the electricity binary all fall to between 43 and 52 percent of their main-anchor values. The

402	original housing binary keeps its sign, at −0.10, but its interval reaches zero, so under the reporting rule of

403	Section 3.7 it is inconclusive on this anchor rather than an inversion. The direction of the anomaly is

404	therefore consistent across all four anchors; its statistical clarity is not, and that is stated here rather than

405	left to be found.

406	**Table 6. Cut-point diagnostic under four anchors, Wave 3.**

| **Binary indicator** | **Main anchor** | **Asset-only anchor** | **Pre-correction anchor** | **Consumption anchor** |
| --- | --- | --- | --- | --- |
| Improved housing,  original analytical coding | −0.26 [−0.33, −0.17] | −0.33 [−0.46, −0.19] | −0.30 [−0.38, −0.19] | −0.10 [−0.24, +0.01] |
| Improved housing, corrected coding | +0.88 [+0.60, +1.14] | +0.58 [+0.35, +0.78] | +0.84 [+0.58, +1.09] | +0.43 [+0.30, +0.56] |
| Clean cooking fuel,  conventional definition | — | — | — | — |
| Cooking-fuel cut at charcoal | +0.75 [+0.53, +0.91] | +0.59 [+0.30, +0.81] | +0.70 [+0.46, +0.86] | +0.39 [+0.20, +0.54] |
| Improved drinking water | +0.32 [+0.22, +0.42] | +0.21 [+0.08, +0.32] | +0.25 [+0.13, +0.36] | +0.31 [+0.20, +0.41] |
| Improved sanitation | +0.35 [+0.24, +0.43] | +0.25 [+0.17, +0.32] | +0.29 [+0.20, +0.36] | +0.17 [+0.10, +0.23] |
| Electricity for lighting | +1.45 [+1.07, +1.84] | +1.25 [+0.99, +1.53] | +1.07 [+0.83, +1.30] | +0.63 [+0.44, +0.82] |

407	Note. Each cell is the weighted difference in location-adjusted wealth between households the binary labels adequate and all

408	others, in the standard-deviation units of the anchor named in the column, with a 95 percent stratified cluster-bootstrap interval

409	from 2,000 replications. The conventional clean-fuel binary captures one household and is not estimable on any anchor. The

410	consumption anchor is the standardised logarithm of per-capita daily consumption; it is available in Wave 3 only.

411	*8.2 The precision floor*

412	The floor of 30 households and 5 enumeration areas is the one analysis choice that could plausibly drive

413	the null in Table 2, since it excludes the categories in which the apparent housing disorder sits. Table 7

414	reports what happens at three alternatives.

415	**Table 7. Sensitivity to the precision floor, Wave 3, all five variables.**

| **Floor** | **Categories classifiable** | **Pairs tested** | **Robust inversions** |
| --- | --- | --- | --- |
| n ≥ 50 and 8 areas | 14 | 14 | 0 |
| n ≥ 30 and 5 areas (adopted) | 16 | 21 | 0 |
| n ≥ 20 and 3 areas | 17 | 25 | 0 |
| No floor | 27 | 73 | 4 |

416	Note. The adopted floor is the middle case. Lowering it to 20 households admits the flat-or-apartment category, which contains

417	22, and changes no verdict.

418	The null is unchanged at every floor except no floor at all. Lowering the threshold to 20 households

419	admits the flat-or-apartment category, the one a referee specifically identified, and still produces no robust

420	inversion. The four inversions that appear when the floor is removed entirely rest on categories containing

421	between one and seven households, including one containing a single household whose bootstrap interval

422	collapses to a point. The choice of floor is therefore not what produces the result.

423	*8.3 The equivalence tolerance*

424	The tolerance of 0.20 standard deviations governs which non-findings may be described as equivalent

425	rather than inconclusive. At 0.10 no pair qualifies. At 0.30 two qualify, but one of them is also

426	significantly different from zero, so that a reader would be told simultaneously that a difference exists and

427	that it is negligible. At 0.20 exactly one pair qualifies and the two verdicts remain disjoint. The tolerance

428	is the largest value that keeps the reporting coherent, and it is applied uniformly.

429	**9. Discussion**

430	**9.1 What the application shows**

431	In this survey the categorical orderings hold up. That is the main empirical result, and it is not a

432	disappointing one: a diagnostic that flagged everything would be worthless, and the value of the exercise

433	lies in its being able to return a null that is bounded by a power calculation rather than merely asserted.

434	The failure is at a cut-point rather than in an ordering, and it is severe. An improved-housing binary

435	constructed during analysis of these data assigns the improved label to the two poorest dwelling types in

436	the survey, and the resulting indicator points the wrong way by a quarter of a standard deviation in two

437	independent waves and under three of the four anchors tried; the fourth, consumption, keeps the sign but

438	not the precision. Any regression using it as a control, any wealth index containing it, and any deprivation

439	count built on it would be affected [9,10], and in the direction that makes housing appear to be negatively

440	associated with wealth.

441	The provenance audit determines how this should be read. Because the survey defines no improved-

442	housing split at all, the error belongs to the analysis rather than to the data: a recoding error in the terms of

443	Section 2. That makes it a smaller claim about survey quality and a larger one about analytical practice:

444	the coding was plausible, undocumented, never checked against the data, and wrong, and none of those

445	four properties is unusual.

446	**9.2 What the paper does not claim**

447	The corrected housing coding is not certified here. The cut-point diagnostic reports that it points the right

448	way; it does not establish that its boundary is the right one, and an alternative set including villa, a

449	category the corrected set omits even though it sits at +0.53 on the wealth gradient, would also point the

450	right way. Choosing between them requires a substantive criterion about dwelling construction, not a

451	wealth comparison, and this paper does not supply one.

452	Nor does the paper claim that any international service definition is incorrect. The clean-fuel result says

453	the indicator carries almost no information about relative wealth in this population, which is a statement

454	about its usefulness as a proxy, not about its validity as a measure of what it actually measures.

455	**9.3 Limitations**

456	Four are worth stating. The evidence is from one survey programme in one country; three waves make the

457	findings more than anecdotal but do not make them general. The anchor is a wealth index built from

458	indicators collected in the same instrument, so a systematic distortion affecting many indicators at once

459	would not be detected by any leave-one-out design; the asset-only anchor mitigates this, and the

460	consumption anchor steps outside the asset and service block altogether, but consumption is the noisiest

461	of the four yardsticks, and the two other external measures a reviewer suggested, education and

462	employment, were not tried, because neither is a measure of living standards in the sense the wealth index

463	is meant to capture. The precision floor means that in a survey of this size the procedure is effectively

464	silent about categories holding fewer than about thirty households, which in practice includes most of the

465	categories at the top of a dwelling-structure distribution in a very poor country. And the cut-point

466	diagnostic requires a binary to exist before it can be evaluated; it offers nothing to an analyst constructing

467	one from scratch, beyond the adjacent-step profile of Section 5.3.

468	**9.4 Practical recommendation**

469	Before a categorical variable is used to build a wealth index, or before a binary derived from one enters a

470	deprivation count, three questions are worth asking, and all three can be answered with the data already in

471	hand. Did this coding come from the instrument, from the codebook, or from an analyst? Do the

472	categories sit on the local wealth gradient in the order the coding assumes, with the design honoured and

473	the anchor's own uncertainty carried through? And does the binary's boundary fall where the gradient

474	actually steps? The procedure set out here answers the second and third in a few minutes of computation.

475	The first, which is the one that resolved the case examined in this paper, requires only that somebody read

476	the codebook.

477	**10. Conclusion**

478	Categorical orderings and the binaries built on them are load-bearing in welfare measurement and are

479	seldom tested. Wealth-Anchored Category Validation tests them against the wealth gradient implied by

480	the rest ofa survey, and reports what it finds as a diagnostic rather than a verdict. Applied to three waves

481	of the South Sudan High Frequency Survey, it finds the orderings sound within the limits a power

482	analysis makes explicit, and finds one binary, constructed during analysis rather than supplied by the

483	survey, that points the wrong way in every wave in which it can be evaluated. The general lesson is not

484	that survey categories are unreliable. It is that a cut-point imported from one setting into another is an

485	empirical claim about that setting, and can be checked before it is trusted.

486	**Supporting information**

487	S1 Table. Robustness suite, Wave 3. Number of robust inversions per variable under the baseline and 42

488	alternative specifications: each anchor indicator dropped in turn, every pair dropped in turn, five location-

489	adjustment specifications, and the iterated second pass. (DOCX)

490	S2 Table. All pairwise category comparisons, Wave 3, under the main, asset-only, pre-correction and

491	consumption anchors. For each pair: households in each category, precision-floor status, difference in

492	location-adjusted wealth, 95 percent stratified cluster-bootstrap interval, bootstrap p-value, Benjamini–

493	Hochberg adjusted p-value, and the equivalence and robust-inversion flags. (DOCX)

494	S3 Table. All pairwise category comparisons in Waves 1 and 2, main anchor, with the same columns as

495	S2 Table. (DOCX)

496	**Data and code availability**

497	The microdata analysed in this study are third-party data owned by the World Bank Microdata Library

498	and are not redistributed by the author, because the Library's terms of use prohibit redistribution: users

499	agree that the data "will not be redistributed or sold to other individuals, institutions, or organizations

500	without the written agreement of the World Bank Microdata Library". The data are otherwise freely

501	available to any researcher. Access is obtained by registering without charge at

502	<https://microdata.worldbank.org>, agreeing to the standard public-use terms, and downloading the SPSS

503	archives from the catalogue entries below. The author obtained the data by this route and received no

504	special access privileges.

505	South Sudan High Frequency Survey 2015, Wave 1 (SSD_2015_HFS-W1_v02_M), catalogue entry

506	2778, DOI 10.48529/bn2b-8q88. South Sudan High Frequency Survey 2016, Wave 2 (SSD_2016_HFS-

507	W2_v02_M), catalogue entry 2777, DOI 10.48529/xz60-7w58. South Sudan High Frequency Survey

508	2016, Wave 3 (SSD_2016_HFS-W3_v03_M), catalogue entry 2914, DOI 10.48529/8wvp-4g14. Wave 3

509	is the main analysis sample; Waves 1 and 2 are the out-of-sample validation.

510	All author-generated code is archived without restriction at Zenodo under an MIT licence, version 1.0.1, 511	DOI 10.5281/zenodo.22243589. The repository contains the estimator, the wave-harmonisation scripts, 512	the out-of-sample and robustness runners, the simulation, a verification test suite, and a single script that

513	reproduces every number in this paper from the source archives. It includes the exact category codes, the

514	construction of every binary indicator, the survey-weighted principal-components step, the location

515	residualisation, the complete bootstrap loop, and the rare-category rule. No absolute paths are used and

516	the analysis depends only on numpy, pandas and, for reading the SPSS archives, pyreadstat.

517	**References**

518	1. Filmer D, Pritchett LH. Estimating wealth effects without expenditure data—or tears: an application to

519	educational enrollments in states of India. Demography. 2001;38(1):115–32.

520	2. Rutstein SO, Johnson K. The DHS Wealth Index. DHS Comparative Reports No. 6. Calverton (MD): ORC

521	Macro; 2004.

522	3. Sahn DE, Stifel DC. Poverty comparisons over time and across countries in Africa. World Dev.

523	2000;28(12):2123–55.

524	4. Sahn DE, Stifel D. Exploring alternative measures of welfare in the absence of expenditure data. Rev Income

525	Wealth. 2003;49(4):463–89.

526	5. McKenzie DJ. Measuring inequality with asset indicators. J Popul Econ. 2005;18(2):229–60.

527	6. Vyas S, Kumaranayake L. Constructing socio-economic status indices: how to use principal components analysis.

528	Health Policy Plan. 2006;21(6):459–68.

529	7. Smits J, Steendijk R. The International Wealth Index (IWI). Soc Indic Res. 2015;122(1):65–85.

530	8. WHO, UNICEF. Progress on drinking water, sanitation and hygiene: 2017 update and SDG baselines. Geneva:

531	World Health Organization and UNICEF; 2017.

532	9. Alkire S, Foster JE, Seth S, Santos ME, Roche JM, Ballon P. Multidimensional poverty measurement and

533	analysis. Oxford: Oxford University Press; 2015.

534	10. Gallardo M, Santos ME, Villatoro P, Pizarro V. Measuring vulnerability to multidimensional poverty in Latin

535	America. Rev Income Wealth. 2024;70(3):661–96.

536	11. Filmer D, Scott K. Assessing asset indices. Demography. 2012;49(1):359–92.

537	12. Howe LD, Hargreaves JR, Gabrysch S, Huttly SRA. Is the wealth index a proxy for consumption expenditure? A

538	systematic review. J Epidemiol Community Health. 2009;63(11):871–7.

539	13. Montgomery MR, Gragnolati M, Burke KA, Paredes E. Measuring living standards with proxy variables.

540	Demography. 2000;37(2):155–74.

541	14. Wittenberg M, Leibbrandt M. Measuring inequality by asset indices: a general approach with application to

542	South Africa. Rev Income Wealth. 2017;63(4):706–30.

543	15. Kolenikov S, Angeles G. Socioeconomic status measurement with discrete proxy variables: is principal

544	component analysis a reliable answer? Rev Income Wealth. 2009;55(1):128–65.

545	16. Benjamini Y, Hochberg Y. Controlling the false discovery rate: a practical and powerful approach to multiple

546	testing. J R Stat Soc Series B. 1995;57(1):289–300.

547	17. Rao JNK, Wu CFJ. Resampling inference with complex survey data. J Am Stat Assoc. 1988;83(401):231–41.

548	18. Lakens D. Equivalence tests: a practical primer for t tests, correlations, and meta-analyses. Soc Psychol Personal

549	Sci. 2017;8(4):355–62.

550	19. Pape UJ. South Sudan High Frequency Survey, Wave 3 (SSD_2016_HFS-W3_v03_M) [dataset]. Washington

551	(DC): World Bank Microdata Library; 2017. doi:10.48529/8wvp-4g14.

552	20. Pape UJ, Mistiaen JA. Household expenditure and poverty measures in 60 minutes: a new approach with results

553	from Mogadishu. Policy Research Working Paper 8430. Washington (DC): World Bank; 2018.

Figure	[Click here to access/download;Figure;Fig1.tif](https://www2.cloud.editorialmanager.com/pone/download.aspx?id=45845124&guid=cfab6364-e301-4da5-aa24-fc69987746d4&scheme=1)[<https://www2.cloud.editorialmanager.com/pone/download.aspx?id=45845124&guid=cfab6364-e301-4da5-aa24-fc69987746d4&scheme=1>](https://www2.cloud.editorialmanager.com/pone/download.aspx?id=45845124&guid=cfab6364-e301-4da5-aa24-fc69987746d4&scheme=1)

Figure	[Click here to access/download;Figure;Fig2.tif](https://www2.cloud.editorialmanager.com/pone/download.aspx?id=45845126&guid=ed9fdd52-5811-45c6-a6b1-13f9e208c1ec&scheme=1)[<https://www2.cloud.editorialmanager.com/pone/download.aspx?id=45845126&guid=ed9fdd52-5811-45c6-a6b1-13f9e208c1ec&scheme=1>](https://www2.cloud.editorialmanager.com/pone/download.aspx?id=45845126&guid=ed9fdd52-5811-45c6-a6b1-13f9e208c1ec&scheme=1)

Figure	[Click here to access/download;Figure;Fig3.tif](https://www2.cloud.editorialmanager.com/pone/download.aspx?id=45845143&guid=a200d2bb-eb70-470e-8b15-947002e04049&scheme=1)[<https://www2.cloud.editorialmanager.com/pone/download.aspx?id=45845143&guid=a200d2bb-eb70-470e-8b15-947002e04049&scheme=1>](https://www2.cloud.editorialmanager.com/pone/download.aspx?id=45845143&guid=a200d2bb-eb70-470e-8b15-947002e04049&scheme=1)
