# Supplementary material for: Wealth-anchored category validation: A diagnostic for categorical orderings in fragile-state household surveys
Source: PLoS One. 2026 Oct 29;21(10):e0360752. doi: 10.1371/journal.pone.0360752 (PMC13718975; doi:10.1371/journal.pone.0360752)
Supplement: Supplementary file 7 [file pone.0360752.s008.docx]

**Response to Reviewers**

PONE-D-26-32266 · Academic Editor: Prof. Jan Vrba · Decision of 7 August 2026

Revised title: Wealth-Anchored Category Validation: A Diagnostic for Categorical Orderings in Fragile-State Household Surveys

Dear Professor Vrba and Reviewers,

Thank you for two reports that were unusually specific and, on every substantive point, correct. Both reviewers identified the same underlying problem, namely that the paper claimed more than its analysis established, and approached it from different directions. The revision closes that gap from both ends: by strengthening the analysis where it was weak, and by withdrawing the claims that survive neither.

Four changes are large enough to describe before the point-by-point responses, because they affect how the rest of the letter reads.

**1. The paper is now a diagnostic, and the title says so.**

Reviewer 1's opening point and Reviewer 2's first point are the same point, and it is decisive. A category's position on a local wealth gradient is not evidence about whether an international technical standard has been applied correctly. The method now reports divergence and enumerates the possible explanations; it does not adjudicate between them from wealth data alone. The title has changed from "Detecting Coding Errors" to "A Diagnostic for Categorical Orderings", and the offending vocabulary no longer describes anything the method does: "miscoding" and "wealth-validated" are gone entirely; "validated improved set" survives only inside quotation marks, twice, as the name of the withdrawn construction; "coding error" survives only as one of the four defined terms in the new Section 2 taxonomy; "corrected" survives only as the historical label of the recoded housing binary the paper now evaluates as an artefact; and "cleared" appears exactly once, in the Section 3.7 sentence that disclaims it. I list the residue this precisely so that it can be checked.

**2. The above-sample-mean threshold has been removed, not defended.**

Reviewer 2's third point is unanswerable as the method stood, and I have not attempted to answer it. No threshold relative to the sample is used anywhere in the revised paper, and the method constructs no "improved" set of its own. What replaces it is a diagnostic that evaluates a binary somebody has already constructed, asking whether its boundary falls where the wealth gradient actually steps. This evaluates a cut; it never proposes one.

**3. The statistical implementation was rebuilt, and Reviewer 2's suspicions about it were right.**

Every conjecture in Reviewer 2's fifth point turned out to describe the shipped code accurately. The principal-components anchor was unweighted while the category means were weighted; enumeration areas were not resampled within strata; and the standardisation, the anchor, the residualisation and the category means were all estimated once outside the bootstrap loop, so the anchor's own uncertainty was invisible. All are corrected. I also found a further defect neither reviewer could have seen from the manuscript, described immediately below.

**4. I am disclosing a discrepancy that Reviewer 2 came close to and that I should have caught before submission.**

Two problems came to light while preparing this revision, and it is better that they come from me.

The first concerns the improved-housing set. The submitted manuscript stated a rule for forming it: categories whose adjusted mean wealth exceeds the sample mean of zero. The reported results do not follow that rule. Applied to housing, the stated rule selects seven categories covering 73.8 percent of households; the paper reported 8.4 percent, corresponding to dwelling types 5, 8, 9 and 10. The same divergence affected drinking water and lighting. No decision rule in the analysis code produces the reported sets. Reviewer 2 detected the edge of this in noting that villa and flat or apartment also carry positive wealth values; the problem was larger than that. Since the threshold rule has now been removed from the method entirely, and no improved set is constructed anywhere, the discrepancy no longer has anything to attach to. But it existed, and I would rather record it than let it be discovered.

The second concerns the cluster bootstrap. The enumeration-area identifier in these files is unique only within stratum: each of its 26 values recurs across three to seven different strata. The submitted analysis factorised that column alone, which collapsed a design of 156 enumeration areas into 26 pseudo-clusters, each pooling households drawn from several states, and resampled those. Clusters are now identified by the pair (stratum, area), which recovers the 156 areas the design contains. This was the single largest inference defect in the original submission and neither reviewer could have seen it from the manuscript.

One consequence of the rebuild is worth flagging because it runs against expectation. Re-estimating the chain inside the bootstrap loop widens intervals, as Reviewer 2 anticipated; identifying the clusters correctly narrows them, because the original code was resampling a few large heterogeneous pseudo-clusters. For categories above the precision floor the second effect dominates and intervals are about a third narrower than before; for thin categories the first dominates and they are around 40 percent wider. No point estimate moved by more than 0.32 standard deviations.

**Reviewer 1**

I am grateful for a review that separated what the method does from what the manuscript claimed for it. Responses follow the order of the report.

**1.1 The method shows wealth association, not that international definitions are factually incorrect**

*“The method shows which categories within the sample under investigation are associated with higher wealth. It does not, however, prove that internationally defined categories such as 'clean cooking' or 'improved sanitation' are factually incorrect. Charcoal can be a local indicator of prosperity without necessarily being regarded as a clean fuel. The interpretation must therefore be strictly limited to the examination of local wealth gradients.”*

Accepted in full, and it is the change around which the revision is organised. The central claim now states that WACV detects divergence between an assigned categorical ordering and the local wealth ordering, and that divergence is a diagnostic signal with four possible explanations. Charcoal is used in the manuscript as the worked illustration of exactly your point: the revised text states that charcoal is not a clean fuel, that the clean-fuel standard is not attempting to measure prosperity, and that the finding concerns the indicator's usefulness as a wealth proxy in this population rather than its validity as a measure of what it measures.

**Where:** Sections 1, 2, 3.4, 5.2 and 9.2; title and abstract.

**1.2 Document the origin of the alleged housing error**

*“Furthermore, the origin of the alleged housing error must be clearly documented. It must be clear whether the original classification stems from the dataset, the codebook or the authors' own recoding. Otherwise, it remains unclear whether a survey error is actually being corrected or merely an unsuitable analytical operationalisation.”*

I examined the questionnaire and the codebook directly and the answer is unambiguous: it is my own analytical recoding. The survey records dwelling structure as a plain ten-category question and defines no improved/unimproved split over it. The World Bank's derived variables cover water, sanitation, lighting and cooking; there is no derived housing variable. Both the original set and the corrected set were created during analysis. The manuscript now says so plainly and describes the case as an analytical recoding error rather than a survey error.

The audit also produced a likely mechanism, which I think improves the paper. The dwelling codes run 1, 3, 4, 5, 6, 7, 8, 9, 10, 11; there is no code 2. A rule expressed as "types 1 to 3" therefore selects tents and stick tukuls and nothing else, which is 25.9 percent of the sample and the two poorest dwelling types. Many codebooks list the most substantial dwelling first; this survey lists the least substantial first. A rule imported from a survey with the opposite convention selects precisely this set.

**Where:** New Section 4.3; Section 9.1.

**1.3 Distinguish coding errors, recoding errors and context-dependent classification**

*“...the terms 'coding errors', 'recoding errors' and 'context-dependent classification' should be clearly distinguished from one another.”*

A new Section 2 does nothing else. It sets out four sources of divergence: coding error in the instrument, recoding error in the analysis, context-dependent classification where the global standard is right but does not track local prosperity, and sampling noise. A table states for each where it lives and whether wealth data alone can identify it. The answer for the first three is no, which is the point. The terms are then used consistently, and the paper resolves its own two cases explicitly: housing as a recoding error, cooking fuel as a context-dependent classification.

**Where:** New Section 2 and Table 1.

**1.4 Bootstrap confidence intervals for pairwise differences; non-overlap is not a test**

*“The statistical inference must also be clarified. Non-overlapping individual intervals do not replace a direct test of differences between categories. Bootstrap confidence intervals for pairwise mean differences are required...”*

Corrected. Differences between category means are now taken directly from the bootstrap distribution and confidence intervals are reported for the differences themselves. The non-overlap of two marginal intervals is not used as a test anywhere in the revised paper, and a sentence in Section 3.3 says so explicitly.

**Where:** Section 3.3; every pairwise result, with its difference interval and adjusted p-value, in S2 Table (Wave 3) and S3 Table (Waves 1 and 2).

**1.5 Account for multiple comparisons**

*“...as is an account of multiple comparisons...”*

Five variables generate a large family of pairwise comparisons. The false discovery rate is now controlled across the entire family, not variable by variable, by the Benjamini–Hochberg procedure at q = 0.05. The simulation in Section 7 confirms the control is working: across 4,800 simulated datasets with known ground truth, no genuinely concordant pair was ever flagged.

**Where:** Section 3.3; Section 7 and Table 5.

**1.6 Fuller description of the stratified sampling design**

*“...and a more detailed description of the stratified sampling design.”*

Section 4.1 now describes the design in full: 14 strata formed by state crossed with urban/rural status; enumeration areas as primary sampling units drawn with probability proportional to size from the 2008 Census; 12 households listed and drawn within each selected area; weights as the inverse probability of selection, decomposed into area and within-area components and scaled to stratum household counts; and the replacement rules for areas and households. The bootstrap now honours that design by resampling areas within strata.

**Where:** Section 4.1; Section 3.6.

**1.7 Limit the scope of the claims**

*“The scope of the study's claims must also be limited, as the method is tested on only one wave of data collection in a single country. It is therefore not yet possible to speak of comprehensive validation.”*

All language implying comprehensive validation has been removed, and Section 2 closes by stating the scope explicitly: one survey programme, one country, three waves, and no licence for general claims about how international standards track wealth elsewhere. I have also widened the evidence base rather than only narrowing the claim: the method is now applied to Waves 1 and 2 as well, which is the subject of Section 6.

**Where:** End of Section 2; Sections 6 and 9.3.

**1.8 Complete replication code in a public repository with a DOI**

*“Finally, the complete replication code must be deposited in a public repository with a permanent link or DOI prior to acceptance.”*

Agreed, and this was a fair objection to the original data availability statement, which said only that materials were available from the author. The full repository is deposited at Zenodo under an MIT licence (version 1.0.1, DOI 10.5281/zenodo.22243589) and is cited in the manuscript and in the data availability statement. It holds the estimator, the wave-harmonisation scripts, the out-of-sample and robustness runners, the simulation, a verification test suite, and a single script that reproduces every number in the paper from the source archives, together with everything Reviewer 2 listed: the exact category codes, the construction of every binary, the weighted principal-components step, the residualisation, the complete bootstrap loop and the rare-category rule. The absolute path hard-coded in the original script has been removed, and the code depends only on numpy, pandas and pyreadstat. As a check on the deposit itself, the Wave 3 analysis was re-run from the archived code before this letter was finalised and reproduced every value in the deposited output files exactly; the 248 numbers in Tables 2 to 7 were then verified against those outputs by the test script included in the repository.

I should also record that the original manuscript's Section 6 claimed four robustness exercises for which no code existed. All four have now been written and are reported in Section 8; had they not been, I would have withdrawn the claims.

**Where:** Data and code availability section; Section 8.

**1.9 Better justify the binary reclassification threshold**

*“In addition, the threshold for binary reclassification should be better justified...”*

The threshold has been removed. See the response to Reviewer 2's third point.

**Where:** Section 3.4.

**1.10 Remove informal phrasing**

*“...informal phrasing should be removed...”*

Done throughout. The passages you are most likely to have had in mind, among them "the cooking-fuel result is the one with teeth", "the fixes are not cosmetic" and "the ability to clear, not just flag, is what makes it credible", are gone; the last of them went along with the word it depended on.

**Where:** Throughout.

**Reviewer 2**

This report changed the paper more than any other single input, in particular by asking questions about the implementation whose answers turned out to be defects rather than misunderstandings. I am grateful for the precision of it.

**2.1 Confusion between prosperity indicators and substantive service quality**

*“WACV checks whether categories correspond to a relative prosperity index. However, this does not automatically imply that a category that deviates from this index is incorrectly coded... The method should therefore either be explicitly described as a diagnosis of a deviation from the local prosperity order or be validated by external technical or health-related criteria. The current terms 'miscoding', 'corrected', and 'wealth-validated improved set' go beyond what the analysis actually demonstrates.”*

I have taken the first of the two options you offer. The method is now described exactly as a diagnosis of deviation from the local prosperity order, and the three terms you name no longer describe anything the method does; the opening section of this letter states precisely what residue of that vocabulary remains and why. The charcoal example is retained and reframed: the revised text states that the cooking-fuel variable's information about relative wealth is concentrated at a boundary the clean-fuel indicator does not use, so an analyst using that indicator as a wealth proxy will capture almost nothing, and that the appropriate response is to stop using it as a proxy, not to redefine clean cooking.

**Where:** Sections 1, 2, 3.4, 5.2, 9.2; title and abstract.

**2.2 No independent validation of the method**

*“The method was developed after the author observed an unexpected negative association for 'improved housing' in the same dataset and subsequently examined the housing categories. The rediscovery of this case, which had already been used in the development of the method, is then presented as independent confirmation... For a publication on methodology, at least one of the following additions should be included: Application to a survey wave not previously examined or to another country, a simulation study with known correct categories and intentionally swapped categories, external validation based on the questionnaire, the codebook, or documented field information.”*

You asked for at least one of the three. All three are now in the paper, because each answers a different question.

Out-of-sample application (Section 6). The estimator was applied without modification to Waves 1 and 2, neither of which played any part in developing the method. The housing cut-point inversion replicates: Wave 2 returns −0.277 standard deviations against Wave 3's −0.263, the same sign and effectively the same magnitude. The failure of the conventional clean-fuel binary replicates more starkly still, capturing no households at all in Wave 1 and three in Wave 2. Two constraints are recorded rather than worked around: Wave 1 does not contain the dwelling-structure question, so housing runs on Wave 2 only, and Wave 2's public file carries neither stratum nor an urban flag, so its bootstrap is stratified by state.

Simulation (Section 7). A synthetic population with a known ordering, a planted inversion of known size, and the survey's design features. 32 cells, 150 replications each, 4,800 datasets. The procedure is correctly calibrated and conservative (2.0 to 6.0 percent false positives against a nominal 5 percent), never flagged a genuinely concordant pair, and recovers an inversion of about 0.30 standard deviations four times in five for a category holding a quarter of households.

Documentary validation (Section 4.3). The codebook audit described in response to Reviewer 1's second point.

I also want to accept the narrower form of your criticism. Leave-one-out removes mechanical circularity but does not make the exercise independent of the dataset the method came from, and the revised Section 3.1 says so in those words rather than implying otherwise.

**Where:** Sections 3.1, 4.3, 6 and 7; Tables 4 and 5.

**2.3 The above-sample-mean threshold is not substantively justified, and the housing implementation is inconsistent**

*“This threshold is purely relative and depends directly on the composition of the sample in question. A category may be above average in a very poor population and yet still be objectively inadequate... In addition, the specific implementation regarding housing types appears inconsistent. Types 5, 8, 9, and 10 are listed as corrected categories. At the same time, the table also shows positive wealth values for 'Villa' and, in particular, 'Flat or apartment'... If categories were excluded due to a small number of cases or other rules, this decision-making rule must be clearly defined in advance and applied consistently.”*

The threshold is removed. Your reductio was if anything understated: under the stated rule an open-water drinking source, at +0.008 with 1,532 households, would have been classified as improved. No threshold relative to the sample appears anywhere in the revised method.

One small correction, offered only so the record is accurate: dwelling type 5 is flat or apartment, and it is inside the set {5, 8, 9, 10} rather than outside it. Your substantive point stands entirely, and it stands on villa, which is type 6, sits at +0.53, and is excluded from that set with no stated justification. As explained in the opening section of this letter, the reported sets did not follow the stated rule at all, and I am disclosing that rather than reconstructing a rationale for it.

On the request for a decision rule fixed in advance and applied consistently: there is now exactly one, stated in Section 3.5 and applied to every variable, every wave and every robustness run. A category is always reported, but is classified and admitted to the pairwise family only if it holds at least 30 households in at least 5 enumeration areas. This also fixes a defect you did not see: the original analysis had no rare-category rule at all, so the biogas category, with one household, returned a bootstrap interval whose endpoints coincided, a point rather than an interval, while Section 6.3 of the submitted manuscript claimed that wide intervals were how the method signalled indecision.

Because this floor is the one analysis choice that could plausibly manufacture the null result, Section 8.2 reports what happens at three alternatives. The answer is nothing: at a floor of 20 households, which admits the flat-or-apartment category you identified, there is still no robust inversion, and the same holds at 50. Only with no floor at all do inversions appear, and those rest on categories holding between one and seven households.

**Where:** Sections 3.4, 3.5 and 8.2; Table 7.

**2.4 'Cleared' is too strong a statistical term**

*“The absence of a significant or 'robust' inversion signal does not constitute proof that the original coding is correct. The wording should therefore be changed to 'no robust evidence of an inversion was detected'. Actual validation or confirmation would require an equivalence procedure, predefined tolerance limits, and information on statistical power. Otherwise, a variable may have 'passed' simply because the precision was too low.”*

Adopted, including your wording, which the manuscript now uses verbatim. The verdict "cleared" has been retired throughout; the estimator now emits "no robust evidence of an inversion", and the word's only remaining occurrence is the sentence in Section 3.7 that disclaims it.

I have supplied all three of the things you say confirmation would require. Adjacent pairs are subjected to two one-sided tests against a tolerance of 0.20 standard deviations fixed in advance, so a pair whose difference interval lies wholly inside that band is described as equivalent within tolerance. Section 8.3 reports why 0.20 and not 0.10 or 0.30. And the simulation supplies power curves, so the paper can state what it could have detected and did not: at Wave 3's size, inversions smaller than roughly a third of a standard deviation in the common categories, or two-thirds in the thin ones, would plausibly have escaped. Results that meet neither test are labelled inconclusive, and several are.

**Where:** Sections 3.7, 7 and 8.3; Table 5.

**2.5 The statistical inference must be described more precisely and completely**

*“...it remains unclear whether the PCA was conducted using survey weights, whether the stratified two-stage sampling design was fully accounted for, whether enumeration areas within the respective strata were resampled, and whether standardization, PCA, residualization, and category means were fully re-estimated in each bootstrap replication... If the Wealth Index was fixed outside the bootstrap loop, the uncertainty of the estimated anchor is underestimated. Spearman's ρ is also of limited suitability as a key metric.”*

I checked each question against the shipped code rather than answering from memory. Your inference was right in every case, and the answers were, in order: no, no, no, and no.

The principal-components anchor used unweighted means, standard deviations and correlations while the category means that consumed it were survey-weighted; the residualisation was likewise unweighted. All are now survey-weighted. Enumeration areas were resampled from the full set without regard to stratum; they are now resampled within each of the 14 strata. And the standardisation, the anchor, the residualisation and the category means were all computed once outside the loop, with only pre-computed area aggregates resampled; exactly as you say, the anchor's uncertainty was invisible. The entire chain is now re-estimated inside every one of 2,000 replications.

Your point about Spearman's ρ was also well taken, and the housing figure of 0.84 was indeed working against the paper's own argument. The headline is now a frequency-weighted discordance ratio, reported alongside the weighted share of households sitting in a category involved in a robust inversion. ρ is retained for comparability and demoted; the manuscript notes that for cooking fuel ρ is −0.20 while the frequency-weighted discordance is 0.002, which is precisely the contrast that justifies the demotion.

**Where:** Sections 3.1, 3.2, 3.3 and 3.6; Table 2.

**2.6 The anchor is leave-one-out, but not truly independent**

*“However, the anchor still consists of closely related variables from the same survey and, in some cases, indicators that have already been corrected based on the data... The robustness analysis should therefore use at least one anchor consisting exclusively of less normatively coded characteristics, such as durable assets.”*

This concern was sharper than the manuscript let you see. The anchor pool contained both the corrected housing indicator and the corrected cooking indicator, so for sanitation, water and lighting the anchor carried two variables that had themselves been adjusted using these data. I should have said so.

Three additional anchors are now reported, and Table 6 sets the cut-point diagnostic under all four side by side. The first uses only durable assets, room density and land ownership, and so contains no normatively coded service indicator at all; this is the anchor you asked for. The second substitutes the original housing coding and the conventional clean-fuel coding, so that no indicator adjusted in the light of these data enters the pool. Both reproduce every verdict in the main specification, and the housing inversion is if anything sharper under them (−0.33 and −0.30 standard deviations against −0.26).

The third takes up your suggestion of an external measure of well-being. Consumption is available in these files, and I have used the logarithm of per-capita daily consumption, standardised and location-adjusted in the same way, as a fourth anchor. It is a noisier yardstick than the composite: its correlation with the durable-asset count is 0.38. The result is reported in full rather than selectively. Every ordering verdict is unchanged. Every cut-point gap is attenuated by roughly half, uniformly, as a noisier anchor implies. The original housing binary keeps its sign, at −0.10, but its interval reaches zero, so under the paper's own reporting rule it is inconclusive on this anchor rather than an inversion. The manuscript says so in Section 8.1 and again in Section 9.1: the direction of the anomaly is consistent across all four anchors, its statistical clarity is not. Education and employment were not tried, because neither is a measure of living standards in the sense the wealth index is meant to capture; Section 9.3 records that.

Section 8.1 adds a fuller robustness suite: each anchor indicator dropped in turn, every pair dropped in turn, five location-adjustment specifications, and an iterated second pass. Thirty-seven of forty-two runs reproduce every baseline verdict exactly. The five that do not all concern a single lighting pair discussed below, and no other verdict moves anywhere in the suite.

**Where:** Sections 3.1 and 8.1; Table 6; S1 Table.

**2.7 Reproducibility**

*“The dataset is generally accessible. However, the analysis code will not be made publicly available until after acceptance and is currently available only from the author... In particular, the following are required: the exact category codes, the creation of all binary variables, the weighted PCA, the residualization, the complete bootstrap loop, and the rules for rare categories. Overall, WACV could be useful as an exploratory tool for identifying unusual prosperity orders. However, the current interpretation of it as a general procedure for detecting and correcting coding errors, as well as for redefining "improved" or "clean" categories, is too broad.”*

The code is deposited with this revision, not after acceptance: Zenodo, MIT licence, version 1.0.1, DOI 10.5281/zenodo.22243589. Each item on your list has a single, named location in the repository. The exact category codes and labels are the SPECS dictionary in wacv_core.py; every binary indicator is constructed in one function, build_indicators, and nowhere else; the survey-weighted principal-components step is _wpca1 and the weighted residualisation is _wresid; the complete bootstrap loop, including the within-stratum cluster resampling and the re-estimation of the whole chain in each replication, is the draw_weights and group_means_batch pair; and the rare-category rule is the two constants MIN_N and MIN_CLUSTERS, applied in one place. A single script, run_all.sh, reproduces every number in the paper from the World Bank archives, and a test suite checks the estimator's equivalences before it runs.

On your closing sentence: I agree with it, and the paper now says what you say. WACV is described as a diagnostic that identifies unusual positions on the local prosperity order and enumerates their possible explanations. It is no longer described as a procedure for detecting or correcting coding errors, and it redefines no "improved" or "clean" category anywhere.

**Where:** Data and code availability section; Sections 1 and 3.4; the deposited repository.

**2.8 A result I am reporting because it disagrees with itself**

One finding sits awkwardly and I would rather present it than let it emerge later. In the lighting variable, households reporting no lighting at all sit above those using grass or firewood, where the coding places them below. In Wave 2 this is a robust inversion (adjusted p = 0.008). In Wave 3 the same pair runs in the same direction but is small enough to be declared equivalent within tolerance, and it crosses the significance threshold in five of the twenty-eight drop-two robustness runs. Consistent direction, inconsistent verdict; and under the consumption anchor added in response to your sixth point, the same pair runs the other way, in the order the coding assumes (adjusted p = 0.004). I no longer read this as a small real effect. The manuscript now says that these data do not resolve the pair, and reports all three results rather than the one that gives the cleanest answer.

**Where:** Sections 6 and 8.1.

**Journal requirements**

**1. PLOS ONE style requirements, including file naming**

The manuscript has been rebuilt against the two PLOS ONE formatting samples: US Letter, double-spaced, continuous line numbering, page numbers, a short running title, and the main headings of the template: Introduction, Materials and methods, Results, Discussion and Conclusion, with the numbered sections beneath them; supporting-information captions follow the Conclusion as in the sample; references are numbered in order of citation in the Vancouver style; and the keywords and JEL lines of the first version, which the template does not use, have been removed. Files are named as required: Manuscript, Revised Manuscript with Track Changes, Response to Reviewers, and S1 to S3 Table.

**2. Author-generated code shared without restriction**

All author-generated code is deposited publicly at Zenodo under an MIT licence (version 1.0.1, DOI 10.5281/zenodo.22243589) and cited in the manuscript and the data availability statement. The repository includes a verification test suite and a single script that reproduces every number in the paper from the source archives.

**3. Deposit data before acceptance**

I am unable to comply, and the reason is a permitted one. The microdata are third-party data owned by the World Bank Microdata Library, whose terms of use state that the data "will not be redistributed or sold to other individuals, institutions, or organizations without the written agreement of the World Bank Microdata Library". I therefore cannot deposit them. They are, however, freely available to any researcher: registration is free, access is automatic for research use, and the data availability statement now gives the exact route and the catalogue entry and DOI for each wave. I received no special access privileges. I would be glad to have the editor's view on whether this satisfies the policy's exemption for third-party data.

**4. DOI or accession number for each dataset**

Added. Wave 1: SSD_2015_HFS-W1_v02_M, catalogue 2778, DOI 10.48529/bn2b-8q88. Wave 2: SSD_2016_HFS-W2_v02_M, catalogue 2777, DOI 10.48529/xz60-7w58. Wave 3: SSD_2016_HFS-W3_v03_M, catalogue 2914, DOI 10.48529/8wvp-4g14. The code repository is at Zenodo, DOI 10.5281/zenodo.22243589.

**5. Full ethics statement in the Methods section**

Added as Section 4.4. It states that the study is a secondary analysis of de-identified, publicly archived microdata; that the author collected no data from human participants and had no contact with respondents; that no institutional review board approval was required and none was sought, because Gambella University's research ethics committee does not require review for secondary analysis of anonymised public-use data; and that informed consent was obtained from participants by the original collectors, the World Bank and South Sudan's National Bureau of Statistics.

**6. Works recommended by reviewers**

Neither reviewer recommended specific publications, so no citations have been added on this ground. I have added references where the revision introduced new methods: Benjamini and Hochberg on false discovery rate control, Lakens on equivalence testing, and Rao and Wu on resampling with complex survey data. The reference list has also been reduced to the twenty works the revised text actually cites, and two entries whose bibliographic details were wrong in the first version have been corrected.

**Summary of new material**

New Section 2 on the four sources of divergence, with Table 1. New Section 4.3 on the provenance of the improved-housing binary. New Section 4.4, the ethics statement. New Section 6, the out-of-sample application to Waves 1 and 2, with Table 4. New Section 7, the simulation study, with Table 5. New Section 8, robustness and analysis choices, with Tables 6 and 7 and S1 Table. S2 and S3 Tables list every pairwise comparison in every wave. Sections 3, 5 and 9 substantially rewritten. New title and abstract. The improved-share headline of 25.9 to 8.4 percent, the 20.8 percent charcoal figure and the description of three variables as cleared have all been withdrawn.

The paper is shorter on claims than the version you read and longer on evidence. I think that is the right trade, and it is one both reports pointed towards.

With thanks for the care both reviewers took,

**Gudi V. Chandra Sekhar**

Department of Economics, Gambella University, Ethiopia
