# Supplementary material for: Wealth-anchored category validation: A diagnostic for categorical orderings in fragile-state household surveys
Source: PLoS One. 2026 Oct 29;21(10):e0360752. doi: 10.1371/journal.pone.0360752 (PMC13718975; doi:10.1371/journal.pone.0360752)
Supplement: Supplementary file 8 [file pone.0360752.s009.docx]

**Response to Reviewers**

PONE-D-26-32266R1 · Academic Editor: Prof. Jan Vrba · Decision of 14 September 2026 (minor revision)

Wealth-Anchored Category Validation: A Diagnostic for Categorical Orderings in Fragile-State Household Surveys

Dear Professor Vrba and Reviewers,

Thank you for the second round of reports. All three reviewers judged the first revision to have resolved the major conceptual and methodological concerns, and the points raised now concern interpretative precision, terminology, transparency of reporting and presentation. I have addressed every one of them. No estimate, table value or figure value has changed; the analysis code is unchanged apart from one added reporting script. The changes are to text, to the labelling of one figure and three tables, and to the supporting information, and they are set out point by point below.

Because the three reports overlap, it may help to list the changes once before the responses. (1) A new paragraph in the Introduction states how WACV differs from existing asset-index validation, category-scoring and cut-off robustness approaches. (2) Section 3.1 now reports the principal-components anchor: eigenvalues, variance explained and loadings, with a new S4 Table giving them for every anchor pool. (3) Section 3.6 states explicitly how the sampling weights travel through the bootstrap. (4) Section 3.7 gives a substantive justification for the 0.20 SD tolerance, states that it was fixed before the revised estimates were produced, and frames the equivalence tests as a complementary sensitivity analysis; Section 8.3 is reworded to match. (5) "Corrected coding" becomes "revised coding" throughout, including Fig 2 and Tables 3, 4 and 6, and the "pre-correction anchor" becomes the "pre-revision anchor". (6) The verdict column of Tables 3 and 4 no longer says "ordered as intended"; it records where the labelled side sits, and a note states that the verdict certifies no boundary. (7) Section 5.3 states that adjacent wealth-gradient steps can inform a wealth-related cut but cannot fix a normative boundary. (8) Section 8.1 discusses whether the attenuation under the consumption anchor reflects anchor noise or sensitivity of the housing result. (9) Sections 6 and 9.4 state how a specification-dependent flag, such as the lighting pair, should be treated, and Section 9.4 now recommends a pre-specified minimum category size. (10) Section 9.3 adds that Wave 2 is not an identical replication setting and that three waves of one programme demonstrate the method without establishing prevalence. (11) The wording of the Conclusion and of Section 9.1 follows Reviewer 5's formulation. (12) The reference list gains one entry (Cohen 1988, cited for the small-effect convention) and is otherwise unchanged; the replication package is re-deposited as version 1.0.2 with the reporting script and the S4 output.

**Reviewer 3**

Thank you for a report that reads the revision closely and asks for exactly the clarifications a careful user of the method would want.

**3.1 Substantive justification of the 0.20 SD tolerance, and whether it was specified in advance**

*“I would nevertheless welcome a brief substantive justification for why 0.20 SD should be interpreted as a meaningful threshold for negligible differences. If this value was not independently specified prior to examining the results, this could simply be stated transparently and the equivalence analysis framed primarily as a complementary sensitivity analysis.”*

Both parts are now in the paper. On the substance: no external standard says what counts as a negligible difference in position on a wealth index, so Section 3.7 gives two reasons for 0.20. It is the conventional boundary below which a standardised difference is described as small (Cohen 1988, now reference 21). And it is close to the resolution of the design in these data: the smallest adjacent step between categories above the precision floor that the main specification resolves as different from zero is 0.16 SD, between stick-walled and mud-walled tukuls, so a tolerance much wider than 0.20 would begin to declare negligible differences that the design itself reports as real. That is precisely what Section 8.3 shows happening at 0.30, where one pair is simultaneously significant and "equivalent".

On provenance: the value was fixed as a constant in the analysis code before the revised estimates were produced, and it was not derived from them. It was not, however, registered outside this study, and the comparison with 0.10 and 0.30 in Section 8.3 was made after the results were known. The paper now says all of this in those terms, and it takes your suggested framing: the equivalence tests are offered as a complementary sensitivity analysis, and the primary bound on the null result is the simulation's power curve. Section 8.3 has been reworded so that it no longer reads as though the coherence check were the ground on which the value was chosen; the sentence that said the tolerance "is the largest value that keeps the reporting coherent" now says that the pre-specified value also happens to be that largest value.

**Where:** Section 3.7 (expanded); Section 8.3 (reworded); reference 21.

**3.2 Wave 2 is not an identical replication setting**

*“Wave 2 lacks both the stratum variable and the urban indicator, meaning that its bootstrap and location adjustment are not fully identical to those used for Wave 3. The manuscript already reports this clearly; I would only suggest briefly emphasizing this point in the limitations section so that cross-wave consistency is not interpreted as a completely identical replication setting.”*

Added, in the limitations and at the point of use. Section 9.3 now states that the out-of-sample waves are not an identical replication setting: Wave 2's public file carries neither a stratum variable nor an urban indicator, so its bootstrap is stratified by state alone and its location adjustment uses state alone, and Wave 1 lacks the dwelling-structure question altogether; cross-wave consistency should be read with those differences in mind. The same caveat is repeated in the note to Table 4, where the Wave 2 housing estimates appear, so that a reader who goes to the table first sees it there.

**Where:** Section 9.3; note to Table 4.

**3.3 Keep the distinction between diagnosing divergence and establishing validity in the practical recommendations**

*“I encourage the author to maintain this distinction consistently in the practical recommendations, particularly where adjacent wealth-gradient "steps" are discussed as potentially informative for selecting a cut-point. Such information may be highly useful when constructing an indicator intended to represent relative wealth, but it cannot by itself establish a normative boundary such as "adequate," "improved," or "clean."”*

Agreed, and the passage you point to was the one place the distinction had slipped. Section 5.3 now says that the adjacent-step profile is useful within a stated limit: it can guide the placement of a cut for a wealth-related purpose, such as an input to a relative wealth index, but it cannot fix a normative boundary. The example is the one you would expect: that a large step separates charcoal from firewood says where the cooking-fuel variable carries its wealth information and nothing about whether charcoal is clean, and an indicator meant to measure adequacy, improvement or safety must take its boundary from the technical or health standard that defines those terms. Section 9.2 now makes the same point for every binary in Table 3, and the verdict column of Tables 3 and 4 has been reworded so that it no longer says "ordered as intended", which implied an endorsement the diagnostic cannot give; it now records only where the labelled side sits, and the table note says that the verdict certifies no boundary.

**Where:** Section 5.3 (second paragraph); Section 9.2; Tables 3 and 4 and their notes; Section 9.4.

**Reviewer 4**

Thank you for a report whose ten points form a useful checklist. Several of them refer to features of the originally submitted manuscript, such as the earlier title, an "Improved cooking share" row in Table 3, a validated improved set defined in Section 3.3, and a statement that the code would be deposited on acceptance, all of which the first revision removed or replaced. Where that is the case I say so and point to where the first revision addressed the point; where the point can be taken further, I have taken it further. I respond to the ten numbered points of the attached report, which contain the summary given in the decision letter.

**4.1 Clarify the novelty of WACV**

*“The Introduction and literature review should state more explicitly how WACV differs from existing asset-index validation, sensitivity analysis, and category-scoring approaches. A concise statement explaining the specific methodological advancement would strengthen the contribution.”*

A new passage in the Introduction does this. It distinguishes three bodies of existing work and states what each takes as given. Asset-index validation asks whether the household ranking an index produces is robust to how the index is built and whether it tracks consumption expenditure (Filmer and Scott; Howe et al.); it takes the coding of each input variable as given. Category-scoring approaches, from the dummy-variable convention of the DHS wealth index to polychoric principal components for ordinal inputs (Kolenikov and Angeles), estimate a score for each response option, but the polychoric variant assumes the assigned ordering is correct, the dummy variant leaves it untested, and in both the variable contributes to the very index against which its own categories would have to be judged. Robustness analysis of deprivation indicators varies the cut-off and reports how the headline rate moves (Alkire et al.); it does not ask whether the categories on either side of the cut sit where the wealth gradient says they should. WACV asks that prior question and differs in three respects: it tests the ordering of categories within a variable rather than the ranking of households across variables; it removes the variable under test from the anchor; and it attaches design-based uncertainty to every pairwise comparison and every cut, with the anchor re-estimated in every replication.

**Where:** Section 1, final paragraph.

**4.2 WACV validates against relative wealth, not technical, environmental or health standards**

*“The authors should clarify that WACV validates categories against relative household wealth rather than independently establishing whether a service is technically, environmentally, or health-wise "improved." This distinction is particularly important for cooking fuel.”*

This is the central change of the first revision and is now the organising principle of the paper: the title, the abstract, Section 1, the taxonomy of Section 2 and Table 1, Section 3.4, Section 5.2 and Section 9.2 all state that a divergence from the local wealth ordering is a diagnostic signal with several possible explanations and not a verdict on a technical standard, and charcoal is used throughout as the worked illustration. In this revision I have added a sentence to Section 9.2 that makes the point in your terms and extends it to every binary in Table 3: WACV locates a category on the local wealth gradient and nothing more; it does not establish, and cannot, whether a category is technically, environmentally or health-wise improved, which are questions for the standard that defines the term. The note to Table 3 now says the same of the verdict column.

**Where:** Sections 1, 2, 3.4, 5.2 and 9.2; note to Table 3.

**4.3 Terminology surrounding cooking fuel in Table 3**

*“Table 3 labels the WACV classification as an "Improved" cooking share although charcoal is identified because of its association with relative wealth. Consider terminology such as "wealth-validated higher-status cooking category" rather than "improved" or "clean," unless supported by an external health or environmental standard.”*

The "Improved cooking share" row, and the improved-share construction behind it, were withdrawn in the first revision; the method no longer classifies any cooking category as improved, and Table 3 now reports the cut-point diagnostic. The charcoal row is labelled "Cooking-fuel cut placed one category lower, at charcoal", which describes the cut being evaluated and attaches no status to it. I have not adopted "wealth-validated", because Reviewers 1 and 2 objected to that term in the first round and it was removed on their advice; the neutral description of the cut serves the same purpose. What remained of the old vocabulary was the verdict "ordered as intended" in Tables 3 and 4, and this revision replaces it with "labelled side sits higher" or "inversion: labelled side sits lower", with a note that the verdict records the sign and precision of the gap and certifies no boundary.

**Where:** Table 3 and its note; Table 4; Section 5.2.

**4.4 Justification of the zero/sample-mean cut-point, and sensitivity to alternatives**

*“Section 3.3 defines the validated improved set as categories with adjusted mean wealth above zero. Please provide a clearer theoretical or empirical justification for this threshold. A brief sensitivity analysis using alternative thresholds would further strengthen the approach.”*

The threshold no longer exists. The first revision removed it rather than defending it, for the reason Section 3.4 gives: in a population where roughly four households in five live below the extreme-poverty line, being above the local average is not evidence of adequacy in any technical sense, and in these data the rule would have classified an open-water drinking source as improved. Because no threshold relative to the sample is used anywhere, there is no alternative threshold to vary; the cut-point diagnostic of Section 3.4 evaluates a binary somebody has already constructed and proposes none. The adjacent-step profile of Section 5.3 reports where the gradient steps, which is the information a threshold sensitivity analysis would have been reaching for, and Section 5.3 now states explicitly what that information can and cannot be used for.

**Where:** Sections 3.4 and 5.3.

**4.5 Reporting of the principal-components anchor**

*“The Methods section should provide basic information on PCA construction, including the variance explained by the first component and, preferably, principal component loadings or a supplementary table.”*

Added. Section 3.1 now reports that in Wave 3 the first component of the full eight-indicator pool has an eigenvalue of 2.72 and explains 34.0 percent of the total variance, against 15.2 percent for the second; that across the five leave-one-out pools actually used as anchors the first component explains between 32.7 and 37.6 percent; and that every loading is positive, from 0.22 for improved water to 0.45 for improved sanitation, with the durable-asset count at 0.42, so the component is a general wealth factor rather than a contrast between subsets of indicators. A new S4 Table gives eigenvalues, variance shares and first-component loadings for the full pool, for each of the five leave-one-out pools, for the asset-only pool and for the pre-revision pool. One entry in that table is worth pointing out: in the pre-revision pool, which contains the original housing binary, that binary loads at −0.28, the only loading of any size below zero in any pool (the conventional clean-fuel binary, which marks a single household, loads at −0.02), which is the housing anomaly of Section 5.2 seen from the other side. The statistics are produced by a short reporting script, pca_report.py, added to the deposited code as version 1.0.2; it re-runs the same weighted standardisation, weighted correlation matrix and eigen-decomposition as the estimator on the same analysis samples, and the verification script now checks the figures quoted in Section 3.1 against its output.

**Where:** Section 3.1 (new second paragraph); S4 Table; deposited code v1.0.2.

**4.6 How weights, strata and enumeration-area clustering enter the bootstrap**

*“Please briefly specify how survey weights, strata, and enumeration-area clustering were handled during bootstrap resampling, particularly given the complex survey design.”*

Strata and clustering were described in the first revision (Section 3.6: enumeration areas identified by the pair (stratum, area) and resampled with replacement within each of the 14 design strata, with households travelling with their area; Section 4.1 describes the design and the construction of the weights). What was missing was a sentence on the weights, and it is now there. Sampling weights are carried through the resampling as multipliers: in each replication a household's survey weight is multiplied by the number of times its enumeration area was drawn, so an area drawn twice counts twice and an area not drawn counts for nothing, and the weighted standardisation, principal component, residualisation and category means of that replication all use the product. The weights themselves are not re-estimated; the design that generated them is taken as fixed.

**Where:** Section 3.6, first paragraph; Section 4.1.

**4.7 Very small category sizes and a minimum category-size rule**

*“Some categories contain very few observations, including multi-storey houses, tents, villas, and wooden houses. Although the manuscript acknowledges this limitation and uses bootstrap intervals, the authors should state whether a minimum category-size rule is advisable for future WACV applications.”*

A minimum category-size rule is already part of the method: Section 3.5 fixes a precision floor of 30 households in at least 5 enumeration areas in advance and applies it to every variable, every wave and every robustness run, so that the categories you list are reported but never classified, and Section 8.2 with Table 7 reports the results at three alternative floors. What the paper did not do was say in so many words that future applications should adopt such a rule, and Section 9.4 now does: a minimum category size should be fixed before the procedure is run, applied to every variable and every specification, and reported with the results; the floor used here is a reasonable default for a survey of this size; and the sensitivity to it should be reported as in Section 8.2.

**Where:** Sections 3.5, 8.2 and 9.4.

**4.8 External validity: demonstration of the method, not prevalence**

*“Because the empirical application is based on one wave of one fragile-state survey, the Discussion should emphasize that the findings demonstrate the method rather than establish the prevalence of coding errors across fragile states. Validation using additional survey waves or countries should be identified as an important future research direction.”*

The first revision extended the application to three waves and limited the scope of the claims at the end of Section 2 and in Section 9.3. This revision sharpens the limitation in the terms you use: Section 9.3 now states that three waves show that the procedure can be applied and that its principal finding replicates, but cannot establish how common recoding errors of this kind are across fragile-state surveys, and that application to other survey programmes and countries is the natural next step.

**Where:** Section 9.3, first limitation; end of Section 2.

**4.9 Consistency of the data and code availability statements; public repository**

*“The submission states that the analysis code will be deposited in OSF/Zenodo upon acceptance, whereas the manuscript states that it is available from the author. These statements should be made consistent. Providing a public repository and persistent identifier during revision, where possible, would strengthen reproducibility.”*

Both statements you quote belong to the original submission and were replaced in the first revision, when the full code was deposited at Zenodo under an MIT licence and cited by DOI in the manuscript and in the data availability statement. With this revision the package is re-deposited as version 1.0.2, which adds the PCA reporting script and its output (point 4.5) and the relabelled figure source; the estimator and every published estimate are unchanged. The manuscript's data and code availability section, the cover letter and the submission form's data availability statement all cite the same version and DOI.

**Where:** Data and code availability section; cover letter; submission form.

**4.10 Final editorial check: terminology and self-explanatory figure captions**

*“Please ensure consistent terminology throughout the manuscript, particularly for "miscoding," "mis-ranking," "improved," "clean," and "wealth-validated." Figure captions should also be fully self-explanatory, including definitions of marker colours and confidence intervals.”*

Done. "Miscoding", "mis-ranking" and "wealth-validated" do not occur in the manuscript. "Improved" and "clean" are used only as the names of the indicators under test, as in "improved sanitation" and "clean cooking fuel", never as a status the method assigns. "Corrected coding" has become "revised coding" throughout, including in Fig 2 and Tables 3, 4 and 6, at Reviewer 5's suggestion. Every figure caption defines its markers and intervals: the Fig 1 caption defines the dark and pale markers (above and below the precision floor) and the 95 percent stratified cluster-bootstrap intervals; the Fig 2 caption defines the marker as the weighted wealth gap across the cut, the interval, and now also the shaded region left of zero and the three wave symbols, which the legend showed but the caption had not defined; the Fig 3 caption defines the points, the dashed 80 percent power line and the dotted 5 percent false-positive line. The whole manuscript was re-read for typographical and formatting faults; the changes are visible in the tracked-changes file.

**Where:** Throughout; captions to Figs 1 to 3.

**Reviewer 5**

Thank you for a report that checked the manuscript's wording against its own methodological position and found the places where the two had drifted apart.

**5.1 Conclusion wording and the interpretation of negative findings**

*“The statement that WACV "finds the orderings sound within the limits a power analysis makes explicit" appears stronger than the methodological position established elsewhere in the manuscript. Failure to detect a robust inversion should not be interpreted as affirmative validation of an ordering. I recommend wording such as: "WACV finds no robust evidence of ordering inversions in the main specification, within the limits made explicit by the equivalence tests and power analysis." Please check the manuscript for similar wording to ensure consistent interpretation.”*

Adopted, in your words. The Conclusion now reads that WACV "finds no robust evidence of ordering inversions in the main specification, within the limits made explicit by the equivalence tests and power analysis". The check for similar wording turned up two further instances. The opening sentence of Section 9.1, "In this survey the categorical orderings hold up", now reads "In this survey no robust evidence of an ordering inversion is found in any of the five variables tested, within the limits the equivalence tests and the power analysis make explicit". And the opening of Section 5.2, "Six of the seven cuts examined behave as intended", now says that five place their labelled side higher on the wealth gradient than the households they exclude (the first revision said "six of the seven", which was an arithmetic slip: Table 3 has five such rows, one inversion and one non-estimable cut); the corresponding verdict "ordered as intended" in Tables 3 and 4 has been replaced, as described under point 4.3. The abstract and Section 1 already used the "no robust inversion" formulation and are unchanged.

**Where:** Section 10; Sections 9.1 and 5.2; Tables 3 and 4.

**5.2 Consumption-anchor sensitivity of the housing inversion**

*“Under the consumption anchor, the housing inversion becomes less precisely estimated and the confidence interval reaches zero. This result is reported transparently. A brief clarification of whether the attenuation is plausibly attributable to the greater measurement noise of the consumption anchor, or whether it indicates sensitivity of the housing result to anchor construction, would strengthen the robustness discussion.”*

Section 8.1 now weighs the two readings and says which the pattern favours. If consumption were simply a noisier measure of the same latent wealth, every gap measured against it should shrink by a similar proportion; if instead the housing result were sensitive to how the anchor is built, the housing gaps would move while the others held. Table 6 shows the first pattern. The revised housing binary, the charcoal cut, the sanitation binary and the electricity binary fall to between 43 and 52 percent of their main-anchor values, and the original housing binary, at 39 percent of its main-anchor value, sits at the lower edge of that band rather than apart from it. The consumption anchor's correlation of only 0.38 with the durable-asset count points the same way. Measurement noise in the anchor is therefore the more plausible explanation. The paragraph also says what it cannot say: with one consumption measure the two explanations cannot be separated cleanly, and the precision of the housing inversion under this anchor is lost either way. In rewriting the paragraph I also corrected a small overstatement: it had said that "every gap" was attenuated under the consumption anchor, whereas the drinking-water gap is essentially unchanged (0.31 against 0.32), and the text now says so.

**Where:** Section 8.1, paragraph following Table 6's introduction; Table 6.

**5.3 The lighting result and specification-dependent findings**

*“The inconsistent result for the no-lighting versus grass/firewood comparison across waves and alternative anchors is appropriately reported as unresolved. A brief statement explaining how such specification-dependent findings should be interpreted by users of WACV would strengthen the practical guidance provided by the manuscript.”*

Added in two places. At the end of Section 6, immediately after the lighting case, the paper now states the rule it implies: a flag that appears under some anchors, waves or specifications and not others is a candidate for investigation, not an inversion; the report should list the specifications under which it appears and those under which it does not; it licenses no recoding; and only a flag that survives the pre-specified main specification and the anchor perturbations of Section 8 is reported as a robust inversion, which in these data none does. Section 9.4 repeats the rule among the practical recommendations, so that a reader who goes to that section for guidance finds it there.

**Where:** Section 6, final paragraph; Section 9.4.

**5.4 Terminology for the housing recoding**

*“Because WACV does not itself establish that an alternative housing classification is substantively correct, terminology such as "corrected coding" should be used cautiously. Where appropriate, a more neutral expression such as "revised coding" or "alternative coding" would better reflect the diagnostic scope of the method.”*

Adopted throughout. "Corrected coding" and "the corrected set" are now "revised coding" and "the revised set" in Sections 4.3, 5.2, 8.1 and 9.2, in Tables 3, 4 and 6, and in Fig 2, which has been regenerated with the new label. The anchor previously called the "pre-correction anchor", which substitutes the original codings for the revised ones, is now the "pre-revision anchor" in the text, in Table 6 and in S2 Table; because the deposited code keys that pool as precorrection, the note to Table 6 and the caption inside the S2 Table file say so, so that a reader moving between the paper and the code is not misled. The word "corrected" no longer appears in the manuscript.

**Where:** Sections 4.3, 5.2, 8.1, 9.2; Tables 3, 4, 6; Fig 2; S2 and S4 Tables.

**5.5 Final consistency and editorial check**

*“Please ensure that the final manuscript, figures, tables, captions, supplementary materials, and any tracked-changes version consistently reflect the final diagnostic framing of WACV and contain no obsolete wording suggesting that WACV independently "repairs," "validates," "certifies," or "clears" classifications. Please also correct the minor typographical and formatting issues identified during final proofreading.”*

The final text was searched for each of the words you list, and for "corrected", "as intended", "confirmed" and "hold up". What remains are disclaimers only: "cleared" occurs once, in the Section 3.7 sentence that says the paper does not report a coding as cleared, confirmed or validated; "certifies" occurs in Section 5.2 and "certified" in Section 9.2, in sentences stating that the diagnostic certifies no set, and "certify" in the new note to Table 3 saying the same; "repaired" occurs once, in Section 3.4, of the withdrawn threshold rule ("removed rather than repaired"); "validation" survives in the method's name and in the phrase "validated improved set", which appears in quotation marks as the name of the withdrawn construction. The figures, tables, captions and the four supporting-information tables use the same vocabulary as the text. One further inconsistency was found and removed: Section 4.1 and the data availability statement described Waves 1 and 2 as the "out-of-sample validation" while Section 6 is titled "Out-of-sample application"; both now say application. Proofreading also found and corrected the following. Section 5.2 said that six of the seven cuts pointed the intended way when Table 3 shows five (point 5.1). The new Introduction paragraph cited Kolenikov and Angeles before Montgomery et al. and Wittenberg and Leibbrandt, and the new Cohen citation falls before the survey references, so the reference list has been renumbered in order of first appearance (13 to 15 and 19 to 21 change number; no work is added or removed beyond Cohen). Four sentences that referred to the review process ("both referees asked", "the one a referee specifically identified", "a reviewer suggested", "has changed most in revision") are reworded for the published record without changing their substance. The corresponding-author asterisk is now attached to the author name. "Inversions larger than that would not have [escaped detection]" in Section 7 now reads "would most likely not have", since 80 percent power leaves one in five undetected. Two formatting faults are corrected: S2 and S3 Tables had been generated with the page dimensions and orientation flag at odds with one another, which could push the widest columns beyond the right margin, and both are rebuilt on a true landscape page; and the placeholder used for non-estimable cells in Tables 3, 4 and 6 has been standardised. The tracked-changes file is a genuine Word revision-marked document produced from the same source as the clean manuscript, so the two cannot differ in content.

**Where:** Throughout; S1 to S4 Tables; the tracked-changes file.

**Journal requirements**

**Works recommended by reviewers, and the reference list**

No reviewer recommended a specific publication in this round. One reference has been added on my own account: Cohen J, Statistical power analysis for the behavioral sciences (2nd ed., 1988), cited once in Section 3.7 for the convention that a standardised difference of 0.2 is described as small. It is reference 21. No other reference has been added or removed, and none of the works cited has been retracted. Because the new Introduction paragraph cites Kolenikov and Angeles earlier than before and the Cohen citation falls in Section 3.7, the list has been renumbered in order of first appearance: Kolenikov and Angeles is now 13, Montgomery et al. 14, Wittenberg and Leibbrandt 15, Cohen 19, and the two Pape references 20 and 21. Every in-text citation has been checked against the new numbering.

**Files uploaded with this revision**

Manuscript (clean); Revised Manuscript with Track Changes (a Word revision-marked file: every insertion and deletion relative to the version reviewed is recorded and can be accepted or rejected individually); this Response to Reviewers; a cover letter; Figs 1 to 3 (Fig 2 regenerated with the relabelled housing row; Figs 1 and 3 unchanged); and S1 to S4 Tables, of which S4 is new and S2 and S3 are rebuilt as described under point 5.5. The replication package is deposited at Zenodo as version 1.0.2, and the data availability statement in the submission form has been updated to cite it.

With thanks to all three reviewers,

**Gudi V. Chandra Sekhar**

Department of Economics, Gambella University, Ethiopia
