# Supplementary material for: Wealth-anchored category validation: A diagnostic for categorical orderings in fragile-state household surveys
Source: PLoS One. 2026 Oct 29;21(10):e0360752. doi: 10.1371/journal.pone.0360752 (PMC13718975; doi:10.1371/journal.pone.0360752)
Supplement: Peer review history

# Peer review: Decision Letter 0

**DOI:** 10.1371/journal.pone.0360752.r001

**Authors:** Jan Vrba

7 Aug 2026

PONE-D-26-32266 Wealth-Anchored Category Validation: Detecting Coding Errors in Fragile-State Household Surveys PLOS One

Dear Dr. V. Chandra Sekhar,

Thank you for submitting your manuscript to PLOS ONE. After careful consideration, we feel that it has merit but does not fully meet PLOS ONE’s publication criteria as it currently stands. Therefore, we invite you to submit a revised version of the manuscript that addresses the points raised during the review process.

Please submit your revised manuscript by Oct 06 2026 11:59PM. If you will need more time than this to complete your revisions, please reply to this message or contact the journal office at plosone@plos.org. When you're ready to submit your revision, log on to https://www.editorialmanager.com/pone/ and select the 'Submissions Needing Revision' folder to locate your manuscript file.

Please include the following items when submitting your revised manuscript:

- A letter that responds to each point raised by the academic editor and reviewer(s). You should upload this letter as a separate file labeled 'Response to Reviewers'.
- A marked-up copy of your manuscript that highlights changes made to the original version. You should upload this as a separate file labeled 'Revised Manuscript with Track Changes'.
- An unmarked version of your revised paper without tracked changes. You should upload this as a separate file labeled 'Manuscript'.

If you would like to make changes to your financial disclosure, please include your updated statement in your cover letter. Guidelines for resubmitting your figure files are available below the reviewer comments at the end of this letter.

If applicable, we recommend that you deposit your laboratory protocols in protocols.io to enhance the reproducibility of your results. Protocols.io assigns your protocol its own identifier (DOI) so that it can be cited independently in the future. For instructions see: https://journals.plos.org/plosone/s/submission-guidelines#loc-laboratory-protocols. Additionally, PLOS ONE offers an option for publishing peer-reviewed Lab Protocol articles, which describe protocols hosted on protocols.io. Read more information on sharing protocols at https://plos.org/protocols?utm_medium=editorial-email&utm_source=authorletters&utm_campaign=protocols.

As the corresponding author, your ORCID iD is verified in the submission system and will appear in the published article. PLOS supports the use of ORCID, and we encourage all coauthors to register for an ORCID iD and use it as well. Please encourage your coauthors to verify their ORCID iD within the submission system before final acceptance, as unverified ORCID iDs will not appear in the published article. *Only* the individual author can complete the verification step; PLOS staff *cannot* verify ORCID iDs on behalf of authors.

We look forward to receiving your revised manuscript.

Kind regards,

Jan Vrba, prof., Dr, Ph.D., PhDr, MBA., Mgr., Bc.

Academic Editor

PLOS One

Journal Requirements:

When submitting your revision, we need you to address these additional requirements.

1. Please ensure that your manuscript meets PLOS ONE's style requirements, including those for file naming. The PLOS ONE style templates can be found at

https://journals.plos.org/plosone/s/file?id=wjVg/PLOSOne_formatting_sample_main_body.pdf and

https://journals.plos.org/plosone/s/file?id=ba62/PLOSOne_formatting_sample_title_authors_affiliations.pdf

2. Please note that PLOS One has specific guidelines on code sharing for submissions in which author-generated code underpins the findings in the manuscript. In these cases, we expect all author-generated code to be made available without restrictions upon publication of the work. Please review our guidelines at https://journals.plos.org/plosone/s/materials-and-software-sharing#loc-sharing-code and ensure that your code is shared in a way that follows best practice and facilitates reproducibility and reuse.

3. In the online submission form, you indicated that your data will be submitted to a repository upon acceptance. We strongly recommend all authors deposit their data before acceptance, as the process can be lengthy and hold up publication timelines. Please note that, though access restrictions are acceptable now, your entire minimal dataset will need to be made freely accessible if your manuscript is accepted for publication. This policy applies to all data except where public deposition would breach compliance with the protocol approved by your research ethics board. If you are unable to adhere to our open data policy, please kindly revise your statement to explain your reasoning and we will seek the editor's input on an exemption.

4. Please note that your Data Availability Statement is currently missing the DOI/accession number of each dataset OR a direct link to access each database. If your manuscript is accepted for publication, you will be asked to provide these details on a very short timeline. We therefore suggest that you provide this information now, though we will not hold up the peer review process if you are unable.

5. Please include your full ethics statement in the ‘Methods’ section of your manuscript file. In your statement, please include the full name of the IRB or ethics committee who approved or waived your study, as well as whether or not you obtained informed written or verbal consent. If consent was waived for your study, please include this information in your statement as well.

6. If the reviewer comments include a recommendation to cite specific previously published works, please review and evaluate these publications to determine whether they are relevant and should be cited. There is no requirement to cite these works unless the editor has indicated otherwise.

[Note: HTML markup is below. Please do not edit.]

Reviewers' comments:

Reviewer's Responses to Questions

**Comments to the Author**

1. Is the manuscript technically sound, and do the data support the conclusions?

The manuscript must describe a technically sound piece of scientific research with data that supports the conclusions. Experiments must have been conducted rigorously, with appropriate controls, replication, and sample sizes. The conclusions must be drawn appropriately based on the data presented.

Reviewer #1: Partly

Reviewer #2: No

**********

2. Has the statistical analysis been performed appropriately and rigorously?

Reviewer #1: No

Reviewer #2: No

**********

3. Have the authors made all data underlying the findings in their manuscript fully available?

The PLOS Data policy requires authors to make all data underlying the findings described in their manuscript fully available without restriction, with rare exception (please refer to the Data Availability Statement in the manuscript PDF file). The data should be provided as part of the manuscript or its supporting information, or deposited to a public repository. For example, in addition to summary statistics, the data points behind means, medians and variance measures should be available. If there are restrictions on publicly sharing data—e.g. participant privacy or use of data from a third party—those must be specified.

Reviewer #1: No

Reviewer #2: Yes

**********

4. Is the manuscript presented in an intelligible fashion and written in standard English?

PLOS ONE does not copyedit accepted manuscripts, so the language in submitted articles must be clear, correct, and unambiguous. Any typographical or grammatical errors should be corrected at revision, so please note any specific errors here.

Reviewer #1: Yes

Reviewer #2: Yes

**********

5. Review Comments to the Author

Please use the space provided to explain your answers to the questions above. You may also include additional comments for the author, including concerns about dual publication, research ethics, or publication ethics. (Please upload your review as an attachment if it exceeds 20,000 characters)

Reviewer #1: The manuscript addresses a relevant issue in the measurement of household income and poverty and develops a methodologically interesting approach known as ‘Wealth-Anchored Category Validation’. Particular strengths include the ‘leave-one-out’ wealth anchor, the adjustment for location, the cluster bootstrap, and the application to multiple variables. However, substantial revisions are required prior to publication. The method shows which categories within the sample under investigation are associated with higher wealth. It does not, however, prove that internationally defined categories such as ‘clean cooking’ or ‘improved sanitation’ are factually incorrect. Charcoal can be a local indicator of prosperity without necessarily being regarded as a clean fuel. The interpretation must therefore be strictly limited to the examination of local wealth gradients. Furthermore, the origin of the alleged housing error must be clearly documented. It must be clear whether the original classification stems from the dataset, the codebook or the authors’ own recoding. Otherwise, it remains unclear whether a survey error is actually being corrected or merely an unsuitable analytical operationalisation. The statistical inference must also be clarified. Non-overlapping individual intervals do not replace a direct test of differences between categories. Bootstrap confidence intervals for pairwise mean differences are required, as is an account of multiple comparisons and a more detailed description of the stratified sampling design. The scope of the study’s claims must also be limited, as the method is tested on only one wave of data collection in a single country. It is therefore not yet possible to speak of comprehensive validation. Finally, the complete replication code must be deposited in a public repository with a permanent link or DOI prior to acceptance. In addition, the threshold for binary reclassification should be better justified, informal phrasing should be removed, and the terms ‘coding errors’, ‘recoding errors’ and ‘context-dependent classification’ should be clearly distinguished from one another. Overall, the manuscript has potential but requires a fundamental conceptual and statistical revision.

Reviewer #2: The manuscript addresses a relevant problem and is generally well written. However, in its current version, the empirical analysis does not yet sufficiently support the central claim that WACV can be used to identify “coding errors” and correct “improved” or “clean” categories. The following points, in particular, should be fundamentally revised:

1. Confusion between prosperity indicators and substantive service quality

WACV checks whether categories correspond to a relative prosperity index. However, this does not automatically imply that a category that deviates from this index is incorrectly coded. In particular, terms such as “clean cooking,” “improved sanitation,” or “improved water” describe technical, health-related, or infrastructural characteristics and do not primarily reflect a household’s relative wealth.

The problem becomes particularly clear in the example of charcoal: In the sample studied, charcoal can be a characteristic of relatively affluent urban households without necessarily being a “clean” or health-improving fuel. Nevertheless, the manuscript interprets the positive wealth status associated with charcoal as evidence of an error in the coding of the clean fuel indicator.

The method should therefore either be explicitly described as a diagnosis of a deviation from the local prosperity order or be validated by external technical or health-related criteria. The current terms “miscoding,” “corrected,” and “wealth-validated improved set” go beyond what the analysis actually demonstrates.

2. No Independent Validation of the Method

The method was developed after the author observed an unexpected negative association for “improved housing” in the same dataset and subsequently examined the housing categories. The rediscovery of this case, which had already been used in the development of the method, is then presented as independent confirmation.

Although the leave-one-out design prevents the tested variable from being immediately included in the wealth index, it does not make the validation independent of the dataset or the previous development process. For a publication on methodology, at least one of the following additions should be included:

Application to a survey wave not previously examined or to another country,

a simulation study with known correct categories and intentionally swapped categories,

external validation based on the questionnaire, the codebook, or documented field information.

Furthermore, it must be clearly documented whether the original classification of housing types 1–3 actually originates from official survey coding or was created by the author during the analysis. Otherwise, this could be an analytical recoding error rather than a survey error.

3. The threshold “above the sample mean” is not substantively justified

The manuscript defines categories as “validated improved” if their mean residualized wealth score lies above the sample mean of zero.

This threshold is purely relative and depends directly on the composition of the sample in question. A category may be above average in a very poor population and yet still be objectively inadequate, a health hazard, or not “improved” in terms of infrastructure.

Consequently, the reported adjusted percentages of 8.4 percent for housing and 20.8 percent for cooking fuels are not externally validated improvement rates, but rather classifications relative to the sample. The threshold value would either need to be objectively justified and externally calibrated or removed from the method used to assess the ranking.

In addition, the specific implementation regarding housing types appears inconsistent. Types 5, 8, 9, and 10 are listed as corrected categories. At the same time, the table also shows positive wealth values for “Villa” and, in particular, “Flat or apartment”; for flats and apartments, the interval is even significantly above zero.

According to the rule described above, these categories should also be taken into account. If categories were excluded due to a small number of cases or other rules, this decision-making rule must be clearly defined in advance and applied consistently.

4. “Cleared” is too strong a statistical term

For water, sanitation, and lighting, the result is designated as “cleared.” In some cases, however, these decisions are based on few observations, wide confidence intervals, or only weak discriminatory power.

The absence of a significant or “robust” inversion signal does not constitute proof that the original coding is correct.

The wording should therefore be changed to “no robust evidence of an inversion was detected.” Actual validation or confirmation would require an equivalence procedure, predefined tolerance limits, and information on statistical power. Otherwise, a variable may have “passed” simply because the precision was too low.

5. The statistical inference must be described more precisely and completely

The decision regarding a robust inversion is based on the non-overlap of separate 95% confidence intervals.

This is not a direct test of the pairwise difference. Instead, bootstrap distributions of the respective differences between two categories should be calculated, and corresponding confidence intervals reported. In addition, numerous comparisons between categories and variables arise; a strategy for multiple testing is lacking.

Furthermore, it remains unclear

whether the PCA was conducted using survey weights,

whether the stratified two-stage sampling design was fully accounted for,

whether enumeration areas within the respective strata were resampled,

and whether standardization, PCA, residualization, and category means were fully re-estimated in each bootstrap replication.

The manuscript mentions only a cluster bootstrap across 156 enumeration areas.

If the Wealth Index was fixed outside the bootstrap loop, the uncertainty of the estimated anchor is underestimated.

Spearman’s ρ is also of limited suitability as a key metric. For housing, ρ is still 0.84 despite the allegedly significant miscoding.

An inversion ratio weighted by category frequencies or a direct ratio of the affected households would be more meaningful here.

6. The anchor is “leave-one-out,” but not truly independent

Excluding the variable under test in each instance eliminates direct, mechanical circularity. However, the anchor still consists of closely related variables from the same survey and, in some cases, indicators that have already been corrected based on the data.

This allows a shared local prosperity structure to reinforce itself without the need to verify the factual accuracy of the categories.

The robustness analysis should therefore use at least one anchor consisting exclusively of less normatively coded characteristics, such as durable assets. Where available, consumer spending, education, employment, or other external measures of well-being would also be useful as comparative anchors.

7. Reproducibility

The dataset is generally accessible. However, the analysis code will not be made publicly available until after acceptance and is currently available only from the author.

Since several key decisions from the manuscript cannot be fully reconstructed, the code should be made available as part of the revision. In particular, the following are required: the exact category codes, the creation of all binary variables, the weighted PCA, the residualization, the complete bootstrap loop, and the rules for rare categories.

Overall, WACV could be useful as an exploratory tool for identifying unusual prosperity orders. However, the current interpretation of it as a general procedure for detecting and correcting coding errors, as well as for redefining “improved” or “clean” categories, is too broad.

**********

6. PLOS authors have the option to publish the peer review history of their article (what does this mean?). If published, this will include your full peer review and any attached files.

If you choose “no”, your identity will remain anonymous but your review may still be made public.

**Do you want your identity to be public for this peer review?** For information about this choice, including consent withdrawal, please see our Privacy Policy.

Reviewer #1: No

Reviewer #2: No

**********

[NOTE: If reviewer comments were submitted as an attachment file, they will be attached to this email and accessible via the submission site. Please log into your account, locate the manuscript record, and check for the action link "View Attachments". If this link does not appear, there are no attachment files.]

To ensure your figures meet our technical requirements, please review our figure guidelines: https://journals.plos.org/plosone/s/figures

You may also use PLOS’s free figure tool, NAAS, to help you prepare publication quality figures: https://journals.plos.org/plosone/s/figures#loc-tools-for-figure-preparation.

NAAS will assess whether your figures meet our technical requirements by comparing each figure against our figure specifications.

# Author comment: Author response to Decision Letter 1

**DOI:** 10.1371/journal.pone.0360752.r002

2 Sep 2026

A full point-by-point response to the Academic Editor, Reviewer 1 and Reviewer 2 is uploaded as the separate file "Response to Reviewers". In summary: (1) the paper is reframed as a diagnostic of divergence from the local wealth ordering, not a procedure for detecting or correcting coding errors, and the title, abstract and vocabulary now say so; (2) the above-sample-mean threshold and the "validated improved set" are removed rather than defended; (3) the statistical implementation is rebuilt — survey-weighted PCA and residualisation, enumeration areas resampled within strata, the whole estimation chain re-estimated in every bootstrap replication, pairwise difference intervals, Benjamini–Hochberg control, a pre-specified precision floor, equivalence tests and simulation-based power curves; (4) independent validation is added on all three routes Reviewer 2 offered — out-of-sample application to Waves 1 and 2, a planted-swap simulation, and a codebook audit that shows the housing binary was an analytical recoding rather than a survey error; (5) three alternative anchors are reported, including an asset-only anchor and a consumption anchor, with the less favourable consumption result stated in full; (6) two defects in the originally submitted analysis that neither reviewer could see from the manuscript — a rule discrepancy in the improved set and a cluster-identification error that collapsed 156 enumeration areas to 26 — are disclosed and corrected; (7) the complete replication code is deposited at Zenodo with this revision, DOI 10.5281/zenodo.22243589. All six journal requirements are addressed, including the ethics statement in the Methods section and dataset DOIs in the Data Availability Statement. Supporting information S1–S3 Tables are new.

Attachment Submitted filename: Response_to_Reviewers.docx

# Peer review: Decision Letter 1

**DOI:** 10.1371/journal.pone.0360752.r003

**Authors:** Jan Vrba

14 Sep 2026

PONE-D-26-32266R1Wealth-Anchored Category Validation: A Diagnostic for Categorical Orderings in Fragile-State Household SurveysPLOS One

Dear Dr. V. Chandra Sekhar,

Thank you for submitting your manuscript to PLOS One. After careful consideration, we feel that it has merit but does not fully meet PLOS One’s publication criteria as it currently stands. Therefore, we invite you to submit a revised version of the manuscript that addresses the points raised during the review process.

Please submit your revised manuscript by Oct 14 2026 11:59PM. If you will need more time than this to complete your revisions, please reply to this message or contact the journal office at plosone@plos.org. When you're ready to submit your revision, log on to https://www.editorialmanager.com/pone/ and select the 'Submissions Needing Revision' folder to locate your manuscript file.

Please include the following items when submitting your revised manuscript:

- A letter that responds to each point raised by the academic editor and reviewer(s). You should upload this letter as a separate file labeled 'Response to Reviewers'.
- A marked-up copy of your manuscript that highlights changes made to the original version. You should upload this as a separate file labeled 'Revised Manuscript with Track Changes'.
- An unmarked version of your revised paper without tracked changes. You should upload this as a separate file labeled 'Manuscript'.

If you would like to make changes to your financial disclosure, please include your updated statement in your cover letter. Guidelines for resubmitting your figure files are available below the reviewer comments at the end of this letter.

If applicable, we recommend that you deposit your laboratory protocols in protocols.io to enhance the reproducibility of your results. Protocols.io assigns your protocol its own identifier (DOI) so that it can be cited independently in the future. For instructions see: https://journals.plos.org/plosone/s/submission-guidelines#loc-laboratory-protocols. Additionally, PLOS One offers an option for publishing peer-reviewed Lab Protocol articles, which describe protocols hosted on protocols.io. Read more information on sharing protocols at https://plos.org/protocols?utm_medium=editorial-email&utm_source=authorletters&utm_campaign=protocols.

As the corresponding author, your ORCID iD is verified in the submission system and will appear in the published article. PLOS supports the use of ORCID, and we encourage all coauthors to register for an ORCID iD and use it as well. Please encourage your coauthors to verify their ORCID iD within the submission system before final acceptance, as unverified ORCID iDs will not appear in the published article. *Only* the individual author can complete the verification step; PLOS staff *cannot* verify ORCID iDs on behalf of authors.

We look forward to receiving your revised manuscript.

Kind regards,

Jan Vrba, prof., Dr, Ph.D., PhDr, MBA., Mgr., Bc.

Academic Editor

PLOS One

Journal Requirements:

If the reviewer comments include a recommendation to cite specific previously published works, please review and evaluate these publications to determine whether they are relevant and should be cited. There is no requirement to cite these works unless the editor has indicated otherwise.

Please review your reference list to ensure that it is complete and correct. If you have cited papers that have been retracted, please include the rationale for doing so in the manuscript text, or remove these references and replace them with relevant current references. Any changes to the reference list should be mentioned in the rebuttal letter that accompanies your revised manuscript. If you need to cite a retracted article, indicate the article’s retracted status in the References list and also include a citation and full reference for the retraction notice.

[Note: HTML markup is below. Please do not edit.]

Reviewers' comments:

Reviewer's Responses to Questions

**Comments to the Author**

1. If the authors have adequately addressed your comments raised in a previous round of review and you feel that this manuscript is now acceptable for publication, you may indicate that here to bypass the “Comments to the Author” section, enter your conflict of interest statement in the “Confidential to Editor” section, and submit your "Accept" recommendation.

Reviewer #3: (No Response)

Reviewer #4: All comments have been addressed

Reviewer #5: All comments have been addressed

**********

2. Is the manuscript technically sound, and do the data support the conclusions?

The manuscript must describe a technically sound piece of scientific research with data that supports the conclusions. Experiments must have been conducted rigorously, with appropriate controls, replication, and sample sizes. The conclusions must be drawn appropriately based on the data presented.

Reviewer #3: Yes

Reviewer #4: Yes

Reviewer #5: Yes

**********

3. Has the statistical analysis been performed appropriately and rigorously?

Reviewer #3: Yes

Reviewer #4: Yes

Reviewer #5: Yes

**********

4. Have the authors made all data underlying the findings in their manuscript fully available?

The PLOS Data policy requires authors to make all data underlying the findings described in their manuscript fully available without restriction, with rare exception (please refer to the Data Availability Statement in the manuscript PDF file). The data should be provided as part of the manuscript or its supporting information, or deposited to a public repository. For example, in addition to summary statistics, the data points behind means, medians and variance measures should be available. If there are restrictions on publicly sharing data—e.g. participant privacy or use of data from a third party—those must be specified.

Reviewer #3: Yes

Reviewer #4: Yes

Reviewer #5: Yes

**********

5. Is the manuscript presented in an intelligible fashion and written in standard English?

PLOS ONE does not copyedit accepted manuscripts, so the language in submitted articles must be clear, correct, and unambiguous. Any typographical or grammatical errors should be corrected at revision, so please note any specific errors here.

Reviewer #3: Yes

Reviewer #4: Yes

Reviewer #5: Yes

**********

6. Review Comments to the Author

Please use the space provided to explain your answers to the questions above. You may also include additional comments for the author, including concerns about dual publication, research ethics, or publication ethics. (Please upload your review as an attachment if it exceeds 20,000 characters)

Reviewer #3: The revised manuscript addresses an important and often overlooked issue in the construction of welfare measures from categorical household-survey data. I consider the revision to be substantial and successful. In particular, I appreciate that Wealth-Anchored Category Validation is now presented as a diagnostic procedure rather than as a mechanism that can determine from wealth data alone whether a substantive classification is correct or incorrect. The distinction between survey coding error, analytical recoding error, context-dependent classification, and sampling noise considerably improves the conceptual clarity of the manuscript.

The manuscript has also been strengthened methodologically. I particularly appreciate the treatment of the survey design, re-estimation of the full estimation chain within the bootstrap procedure, multiplicity control, the precision rule for sparse categories, out-of-sample application to additional survey waves, simulation-based assessment, and the use of alternative anchors. I also value the transparency with which the author reports and corrects problems identified in the original analysis.

I have only a few relatively minor suggestions:

The manuscript adopts a tolerance of 0.20 standard deviations for equivalence testing and provides a sensitivity discussion showing why values of 0.10 and 0.30 are less useful for reporting. I would nevertheless welcome a brief substantive justification for why 0.20 SD should be interpreted as a meaningful threshold for negligible differences. If this value was not independently specified prior to examining the results, this could simply be stated transparently and the equivalence analysis framed primarily as a complementary sensitivity analysis.

I appreciate the use of Waves 1 and 2 as an additional check on the main findings. However, Wave 2 lacks both the stratum variable and the urban indicator, meaning that its bootstrap and location adjustment are not fully identical to those used for Wave 3. The manuscript already reports this clearly; I would only suggest briefly emphasizing this point in the limitations section so that cross-wave consistency is not interpreted as a completely identical replication setting.

The revised manuscript now makes an important distinction between diagnosing divergence from a local wealth gradient and establishing the substantive or technical validity of a category definition. I encourage the author to maintain this distinction consistently in the practical recommendations, particularly where adjacent wealth-gradient “steps” are discussed as potentially informative for selecting a cut-point. Such information may be highly useful when constructing an indicator intended to represent relative wealth, but it cannot by itself establish a normative boundary such as “adequate,” “improved,” or “clean.”

These points are minor and do not alter my overall positive assessment. In my view, the revision has satisfactorily addressed the major conceptual and methodological concerns raised about the earlier version. The manuscript now presents a transparent and practically useful diagnostic framework while appropriately limiting the interpretation of its findings. Subject to these minor clarifications, I consider the manuscript suitable for publication in PLOS ONE.

Reviewer #4: The manuscript “Wealth-Anchored Category Validation: Detecting Coding Errors in Fragile-State Household Surveys” addresses an important and underexplored methodological issue in household survey research. The proposed Wealth-Anchored Category Validation (WACV) procedure is practically relevant, and its application to the 2016 South Sudan High Frequency Survey provides a useful demonstration of how category miscoding can influence welfare measurement. The leave-one-out wealth anchor, location adjustment, cluster-bootstrap inference, and robustness checks are notable strengths of the study. The manuscript is generally well organized, methodologically sound, and clearly written. However, a few minor revisions are recommended before publication. The authors should more explicitly clarify the methodological novelty of WACV compared with existing asset-index validation and category-scoring approaches. The manuscript should also emphasize that WACV validates categories according to their association with relative household wealth and does not independently establish whether a category is technically, environmentally, or health-wise “improved.” This distinction is particularly important for the cooking-fuel analysis, where charcoal functions as a local wealth marker but should not be interpreted as a clean or improved fuel on that basis alone. The terminology used in Table 3 should therefore be reconsidered. The authors should provide additional justification for using the sample mean/zero as the cut-point for defining the validated improved set and, if feasible, briefly report sensitivity to alternative thresholds. The PCA methodology would benefit from additional reporting, particularly the variance explained by the first component and relevant component loadings. The bootstrap procedure should also clearly explain how sampling weights, strata, and enumeration-area clustering were handled. Given the very small sample sizes for some response categories, the authors should discuss whether a minimum category-size criterion is advisable when applying WACV. The Discussion should more clearly acknowledge that evidence from a single survey wave demonstrates the applicability of the method but cannot establish the prevalence of coding errors across fragile-state surveys. Finally, the data and code availability statements should be made consistent, preferably with the analysis code deposited in a public repository, and the manuscript should undergo a final editorial check for consistent terminology and self-explanatory figure captions. These issues can be addressed without substantial changes to the study design or main analyses. Recommendation: Minor Revision.

Reviewer #5: Dear Authors,

Thank you for submitting your manuscript, “Wealth-Anchored Category Validation: A Diagnostic for Categorical Orderings in Fragile-State Household Surveys.”

Overall, the manuscript presents a rigorous and transparent methodological contribution to the assessment of categorical orderings in household-survey research. A particular strength is the careful positioning of Wealth-Anchored Category Validation (WACV) as a diagnostic of divergence from the local wealth ordering rather than as a procedure that independently establishes whether a substantive classification is correct.

The statistical methodology is well developed. Important strengths include the leave-one-out anchor construction, survey-weighted PCA and residualisation, stratified cluster bootstrap, re-estimation of the complete estimation chain within each bootstrap replication, multiplicity control, a pre-specified precision floor, equivalence testing, simulation-based power assessment, alternative-anchor analyses, and out-of-sample application to additional survey waves. The provenance audit of the housing binary is also valuable because it distinguishes an analytical recoding issue from a defect in the original survey instrument.

The manuscript is close to being suitable for publication. I recommend that the authors address the following minor issues before acceptance:

1. Conclusion wording and interpretation of negative findings.

The statement that WACV “finds the orderings sound within the limits a power analysis makes explicit” appears stronger than the methodological position established elsewhere in the manuscript. Failure to detect a robust inversion should not be interpreted as affirmative validation of an ordering. I recommend wording such as: “WACV finds no robust evidence of ordering inversions in the main specification, within the limits made explicit by the equivalence tests and power analysis.” Please check the manuscript for similar wording to ensure consistent interpretation.

2. Consumption-anchor sensitivity.

Under the consumption anchor, the housing inversion becomes less precisely estimated and the confidence interval reaches zero. This result is reported transparently. A brief clarification of whether the attenuation is plausibly attributable to the greater measurement noise of the consumption anchor, or whether it indicates sensitivity of the housing result to anchor construction, would strengthen the robustness discussion.

3. Lighting result.

The inconsistent result for the no-lighting versus grass/firewood comparison across waves and alternative anchors is appropriately reported as unresolved. A brief statement explaining how such specification-dependent findings should be interpreted by users of WACV would strengthen the practical guidance provided by the manuscript.

4. Terminology concerning the housing recoding.

Because WACV does not itself establish that an alternative housing classification is substantively correct, terminology such as “corrected coding” should be used cautiously. Where appropriate, a more neutral expression such as “revised coding” or “alternative coding” would better reflect the diagnostic scope of the method.

5. Final consistency and editorial check.

Please ensure that the final manuscript, figures, tables, captions, supplementary materials, and any tracked-changes version consistently reflect the final diagnostic framing of WACV and contain no obsolete wording suggesting that WACV independently “repairs,” “validates,” “certifies,” or “clears” classifications. Please also correct the minor typographical and formatting issues identified during final proofreading.

These points do not require fundamental changes to the study design or statistical analysis. They primarily concern interpretative precision, robustness discussion, terminology, and presentation. Subject to satisfactory attention to these minor matters, I consider the manuscript suitable for publication in PLOS ONE.

**********

7. PLOS authors have the option to publish the peer review history of their article (what does this mean?). If published, this will include your full peer review and any attached files.

If you choose “no”, your identity will remain anonymous but your review may still be made public.

**Do you want your identity to be public for this peer review?** For information about this choice, including consent withdrawal, please see our Privacy Policy.

Reviewer #3: **Yes:** Daniela Spiesová

Reviewer #4: **Yes:** Anjel Raj Y

Reviewer #5: No

**********

[NOTE: If reviewer comments were submitted as an attachment file, they will be attached to this email and accessible via the submission site. Please log into your account, locate the manuscript record, and check for the action link "View Attachments". If this link does not appear, there are no attachment files.]

To ensure your figures meet our technical requirements, please review our figure guidelines: https://journals.plos.org/plosone/s/figures

You may also use PLOS’s free figure tool, NAAS, to help you prepare publication quality figures: https://journals.plos.org/plosone/s/figures#loc-tools-for-figure-preparation.

NAAS will assess whether your figures meet our technical requirements by comparing each figure against our figure specifications.

Attachment Submitted filename: review.docx

Attachment Submitted filename: PONE-8-R1.docx

# Author comment: Author response to Decision Letter 2

**DOI:** 10.1371/journal.pone.0360752.r004

21 Sep 2026

A full point-by-point Response to Reviewers is uploaded as a separate file. In summary: all three reviewers judged the first revision to have resolved the major concerns, and every remaining point has been addressed. No estimate, table value or figure value has changed. The changes are: (1) a new Introduction paragraph on how WACV differs from asset-index validation, category-scoring and cut-off robustness approaches (Reviewer 4); (2) Section 3.1 now reports the principal-components anchor, with eigenvalues, variance explained and loadings, and a new S4 Table (Reviewer 4); (3) Section 3.6 states how sampling weights travel through the bootstrap (Reviewer 4); (4) Section 3.7 justifies the 0.20 SD equivalence tolerance, states that it was fixed before the revised estimates were produced, and frames the equivalence tests as a complementary sensitivity analysis, with Section 8.3 reworded to match (Reviewer 3); (5) "corrected coding" becomes "revised coding" throughout, including Fig 2 and Tables 3, 4 and 6, and the "pre-correction anchor" becomes the "pre-revision anchor" (Reviewer 5); (6) the verdict column of Tables 3 and 4 no longer says "ordered as intended", and a note states that the verdict certifies no boundary (Reviewers 4 and 5); (7) Section 5.3 states that adjacent wealth-gradient steps can inform a wealth-related cut but cannot fix a normative boundary (Reviewer 3); (8) Section 8.1 discusses whether the attenuation under the consumption anchor reflects anchor noise or sensitivity of the housing result (Reviewer 5); (9) Sections 6 and 9.4 state how a specification-dependent flag such as the lighting pair should be treated, and Section 9.4 recommends a pre-specified minimum category size (Reviewers 5 and 4); (10) Section 9.3 adds that Wave 2 is not an identical replication setting and that three waves of one programme demonstrate the method without establishing prevalence (Reviewers 3 and 4); (11) the Conclusion and Section 9.1 adopt Reviewer 5's wording on negative findings; (12) one reference is added (Cohen 1988) and the list is renumbered in order of first appearance; the replication package is re-deposited as Zenodo version 1.0.2, DOI 10.5281/zenodo.22870004. Proofreading also corrected an arithmetic slip in Section 5.2 ("six of the seven cuts" is now five) and rebuilt S2 and S3 Tables on a true landscape page.

Attachment Submitted filename: Response_to_Reviewers_auresp_2.docx

# Peer review: Decision Letter 2

**DOI:** 10.1371/journal.pone.0360752.r005

**Authors:** Jan Vrba

30 Sep 2026

Wealth-Anchored Category Validation: A Diagnostic for Categorical Orderings in Fragile-State Household Surveys

PONE-D-26-32266R2

Dear Dr. V. Chandra Sekhar,

We’re pleased to inform you that your manuscript has been judged scientifically suitable for publication and will be formally accepted for publication once it meets all outstanding technical requirements.

Within one week, you’ll receive an e-mail detailing the required amendments. When these have been addressed, you’ll receive a formal acceptance letter and your manuscript will be scheduled for publication.

An invoice will be generated when your article is formally accepted. Please note, if your institution has a publishing partnership with PLOS and your article meets the relevant criteria, all or part of your publication costs will be covered. Please make sure your user information is up-to-date by logging into Editorial Manager at Editorial Manager® and clicking the ‘Update My Information' link at the top of the page. For questions related to billing, please contact billing support.

If your institution or institutions have a press office, please notify them about your upcoming paper to help maximize its impact. If they’ll be preparing press materials, please inform our press team as soon as possible -- no later than 48 hours after receiving the formal acceptance. Your manuscript will remain under strict press embargo until 2 pm Eastern Time on the date of publication. For more information, please contact onepress@plos.org.

Kind regards,

Jan Vrba, prof., Dr, Ph.D., PhDr, MBA., Mgr., Bc.

Academic Editor

PLOS One

Additional Editor Comments (optional):

Reviewers' comments:

Reviewer's Responses to Questions

**Comments to the Author**

1. If the authors have adequately addressed your comments raised in a previous round of review and you feel that this manuscript is now acceptable for publication, you may indicate that here to bypass the “Comments to the Author” section, enter your conflict of interest statement in the “Confidential to Editor” section, and submit your "Accept" recommendation.

Reviewer #1: All comments have been addressed

Reviewer #3: All comments have been addressed

Reviewer #4: All comments have been addressed

**********

2. Is the manuscript technically sound, and do the data support the conclusions?

The manuscript must describe a technically sound piece of scientific research with data that supports the conclusions. Experiments must have been conducted rigorously, with appropriate controls, replication, and sample sizes. The conclusions must be drawn appropriately based on the data presented.

Reviewer #1: Yes

Reviewer #3: Yes

Reviewer #4: Yes

**********

3. Has the statistical analysis been performed appropriately and rigorously?

Reviewer #1: Yes

Reviewer #3: Yes

Reviewer #4: Yes

**********

4. Have the authors made all data underlying the findings in their manuscript fully available?

The PLOS Data policy requires authors to make all data underlying the findings described in their manuscript fully available without restriction, with rare exception (please refer to the Data Availability Statement in the manuscript PDF file). The data should be provided as part of the manuscript or its supporting information, or deposited to a public repository. For example, in addition to summary statistics, the data points behind means, medians and variance measures should be available. If there are restrictions on publicly sharing data—e.g. participant privacy or use of data from a third party—those must be specified.

Reviewer #1: Yes

Reviewer #3: Yes

Reviewer #4: Yes

**********

5. Is the manuscript presented in an intelligible fashion and written in standard English?

PLOS ONE does not copyedit accepted manuscripts, so the language in submitted articles must be clear, correct, and unambiguous. Any typographical or grammatical errors should be corrected at revision, so please note any specific errors here.

Reviewer #1: Yes

Reviewer #3: Yes

Reviewer #4: Yes

**********

6. Review Comments to the Author

Please use the space provided to explain your answers to the questions above. You may also include additional comments for the author, including concerns about dual publication, research ethics, or publication ethics. (Please upload your review as an attachment if it exceeds 20,000 characters)

Reviewer #1: The second revision satisfactorily addresses the remaining concerns. The methodological approach, statistical procedures, and interpretation of the findings are clearly presented. The limitations are appropriately acknowledged, and data and code availability are adequately documented. The manuscript meets the scientific requirements for publication. I recommend acceptance in its present form.

Reviewer #3: Thank you for the careful and comprehensive revision. The remaining issues raised in the previous round have been addressed satisfactorily. In particular, the revised manuscript now provides a transparent justification and appropriate interpretation of the 0.20 SD equivalence tolerance, clearly acknowledges the non-identical nature of the cross-wave application, and consistently distinguishes wealth-gradient diagnostics from normative or technical classification. The terminology has also been aligned with the diagnostic scope of WACV, and the limitations and practical recommendations are now appropriately framed. I have no further substantive comments. The manuscript is suitable for publication in its present form.

Reviewer #4: The author has satisfactorily addressed the concerns raised during the previous review rounds. The revised manuscript demonstrates substantial improvement in methodological transparency, analytical interpretation, and presentation.

The clarification of the Wealth-Anchored Category Validation (WACV) procedure, particularly its diagnostic rather than confirmatory role, strengthens the methodological contribution. The additional reporting of the principal-components wealth anchor, treatment of sampling weights within the bootstrap procedure, justification of the equivalence tolerance, and revised interpretation of negative findings appropriately address the earlier concerns.

The revisions to terminology, tables, figures, and supplementary materials further improve the clarity and reproducibility of the study. The updated replication package is also a positive addition.

I have no further substantive concerns. The manuscript is suitable for publication in its revised form.

**********

7. PLOS authors have the option to publish the peer review history of their article (what does this mean?). If published, this will include your full peer review and any attached files.

If you choose “no”, your identity will remain anonymous but your review may still be made public.

**Do you want your identity to be public for this peer review?** For information about this choice, including consent withdrawal, please see our Privacy Policy.

Reviewer #1: No

Reviewer #3: **Yes:** Daniela Spiesová

Reviewer #4: **Yes:** Anjel Raj Y

**********

# Editor report: Acceptance letter

**DOI:** 10.1371/journal.pone.0360752.r006

**Authors:** Jan Vrba

PONE-D-26-32266R2

PLOS One

Dear Dr. Sekhar,

I'm pleased to inform you that your manuscript has been deemed suitable for publication in PLOS One. Congratulations! Your manuscript is now being handed over to our production team.

At this stage, our production department will prepare your paper for publication. This includes ensuring the following:

* All references, tables, and figures are properly cited

* All relevant supporting information is included in the manuscript submission,

* There are no issues that prevent the paper from being properly typeset

You will receive further instructions from the production team, including instructions on how to review your proof when it is ready. Please keep in mind that we are working through a large volume of accepted articles, so please give us a few days to review your paper and let you know the next and final steps.

Lastly, if your institution or institutions have a press office, please let them know about your upcoming paper now to help maximize its impact. If they'll be preparing press materials, please inform our press team within the next 48 hours. Your manuscript will remain under strict press embargo until 2 pm Eastern Time on the date of publication. For more information, please contact onepress@plos.org.

You will receive an invoice from PLOS for your publication fee after your manuscript has reached the completed accept phase. If you receive an email requesting payment before acceptance or for any other service, this may be a phishing scheme. Learn how to identify phishing emails and protect your accounts at https://explore.plos.org/phishing.

If we can help with anything else, please email us at customercare@plos.org.

Thank you for submitting your work to PLOS One and supporting open access.

Kind regards,

PLOS One Editorial Office Staff

on behalf of

prof. Jan Vrba

Academic Editor

PLOS One
